# Inhibition of prolyl-tRNA synthetase and efflux pumps as a dual-targeting strategy against multidrug-resistant bacteria

**DOI:** 10.1080/14756366.2026.2640718

**Published:** 2026-03-18

**Authors:** Cristiane Tambascia, Jaqueline Cristina Silva, Barbara Carvalho dos Reis, Camila Fernanda Silva Camilo, Carlos Abrunhosa Tairum Junior, Thais Hancio, Valquiria Graia Correia, Ronaldo Aloise Pilli, Andre Schützer de Godoy, Benoît Laleu, Maurício Luís Sforça, Silvana Aparecida Rocco, Celso Eduardo Benedetti, Gustavo Fernando Mercaldi

**Affiliations:** a Brazilian Biosciences National Laboratory (LNBio), Brazilian Centre for Research in Energy and Materials (CNPEM), Campinas, SP, Brazil; b Faculdade de Ciências Farmacêuticas (FCF), Universidade Estadual de Campinas (UNICAMP), Campinas, SP, Brazil; c Instituto de Química, Universidade Estadual de Campinas (UNICAMP), Campinas, SP, Brazil; d MMV Medicines for Malaria Venture, ICC, Geneva, Switzerland

**Keywords:** *Klebsiella pneumoniae*, efflux pump inhibitors, antimicrobial resistance, gram-negative bacteria, aminoacyl-tRNA synthetase inhibitors

## Abstract

Aminoacyl-tRNA synthetases have been widely exploited as targets for antiparasitic and antifungal inhibitors; however, they have received little attention as targets in multidrug-resistant (MDR) bacteria. Here we describe the biochemical characterisation of Prolyl-tRNA synthetase from *Klebsiella pneumoniae* (KpProRS), highlighting its ligase and proofreading activities. Distinct classes of ProRS inhibitors were evaluated against KpProRS but only halofuginone (HF) strongly modulated KpProRS activity. A new HF analog (Cpd-6) was developed and exhibited superior inhibitory activity against KpProRS relative to HF but low efficacy against MDR *K. pneumoniae*, despite good antimicrobial activity against *Escherichia coli* and *Staphylococcus aureus.* Further studies revealed that Cpd-6 resistance in *K. pneumonia* is mainly mediated by *AcrAB* efflux pump activity, which could be counteracted by efflux pump inhibitors. These findings therefore reinforce KpProRS as a target for antimicrobial development and highlight the therapeutic potential of combining HF analogues with efflux pump inhibitors to fight Gram-negative MDR pathogens.

## GRAPHICAL ABSTRACT

**Figure:**
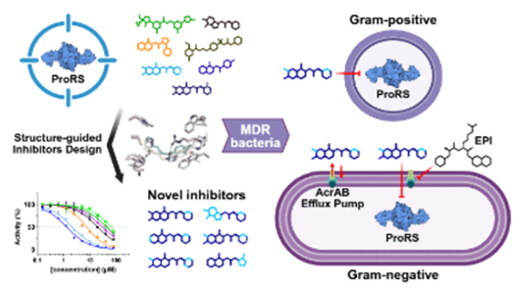

## Introduction

Aminoacyl-tRNA synthases (aaRS), also known as aminoacyl-tRNA ligases, are essential enzymes that play fundamental roles in protein synthesis in both prokaryotes and eukaryotes. These enzymes catalyse the covalent linkage of amino acids to the 3′ ends of cognate tRNAs via a two-step reaction. In the first step, the amino acid reacts with ATP to form the intermediate aminoacyl-adenylate, whereas in the second step, the aminoacyl group is transferred to the adenosine 76 (A76) of the amino acid acceptor arm of the tRNA^1^. Although aaRS are classified into distinct categories based on their three-dimensional structures, domain architecture and folding, all canonical aaRS possess the ATP, amino acid and tRNA acceptor arm binding sites.

Several aaRS have been exploited as drug targets due to their essential role in protein translation and structural differences between aaRS from human and those of parasites, bacterial and fungal pathogens^2–7^. Among them, prolyl-tRNA synthases (ProRS) have proven to be excellent drug targets in protozoans. For instance, ProRS from the human apicomplexan parasites *Plasmodium, Leishmania, Toxoplasma, Cryptosporidium* and *Eimeria* are strongly inhibited by ATP mimetics and halofuginone (HF), a halogenated derivative of the traditional anti-malarial herbal medicine febrifugine (FF)^8–10^.

Structure-based drug design of HF derivatives and ATP mimetics has further led to the development of more potent and specific inhibitors against protozoans relative to human ProRS, which also binds HF and has become a therapeutic target in some cancer and other human diseases^9–17^. On the other hand, bacterial ProRS have been poorly characterised biochemically and structurally, and have not yet been fully explored as antimicrobial targets, particularly in Gram-negative bacteria^18^^,^^19^. This limitation is further complicated in such pathogens by inherent permeability barriers and efflux that restrict compound accumulation, commonly undermining translation of enzyme inhibition to antibacterial activity^20–22^.

The *Staphylococcus aureus* ProRS (SaProRS) is, to date, the only bacterial ProRS known to be moderately inhibited by HF^23^. Despite major structural differences found between eukaryotic and prokaryotic ProRS, the crystal structure of SaProRS in complex with HF revealed that the binding mode of HF in SaProRS is virtually the same as that of the human and apicomplexan enzymes i.e., the quinazolinone and piperidine rings of HF occupy the tRNA A76 and proline sites, respectively^8^^,^^12^^,^^18^^,^^23^. Nonetheless, the existence of polymorphisms in residues of the proline, A76 and ATP sites between eukaryotic and prokaryotic ProRS suggests that HF analogs of greater selectivity for the prokaryotic enzymes can be sought for the development of novel antibiotics.

Accordingly, effective antibacterial strategies targeting intracellular enzymes must address not only target engagement but also the cellular determinants that limit compound accumulation. Here, we present the biochemical and enzymatic properties of ProRS from *Klebsiella pneumoniae* (KpProRS), one of the five leading human Gram-negative pathogens in the world responsible for thousands difficult-to-treat and fatal cases of pneumonia each year^24^^,^^25^. From an initial set of known ProRS modulators, HF proved to be the only KpProRS inhibitor with potency in the low micromolar range. By exploring the amino acid polymorphisms existing between eukaryotic and prokaryotic ProRS at the A76 and proline sites, we made substitutions in the HF structure, according to KpProRS structural models, to achieve higher affinity for the recombinant KpProRS enzyme and greater antimicrobial activity against a multidrug resistant (MDR) *K. pneumonia* and other critical bacterial pathogens. Herein, we present a novel HF analogue (Cpd-6) that displays stronger inhibitory activity upon KpProRS relative to HF and FF, and good antimicrobial activity against *Escherichia coli* and methicillin-resistant *S. aureus*, but weak activity against MDR *K. pneumoniae* and *Burkholderia cenocepacia*. Through genome-based resistome prediction, we identified RND family efflux pumps as one of the major mechanisms of antimicrobial resistance in *K. pneumoniae* and *B. cenocepacia.* Furthermore, through biochemical and genetic studies, we provide evidence that the *AcrAB* efflux pump plays a critical role in Cpd-6 resistance in *K. pneumoniae*. Our findings thus reveal KpProRS as a valid target for antimicrobial development and highlight the therapeutic potential of combining HF analogues with efflux pump inhibitors (EPIs).

## Materials and methods

### Compounds and reagents

Cpd-6, Cpd-7, d-HF and the bersiporocin analogue BSP-1 were synthesised by Bioduro-Sundia (identity and purity data provided in supplemental information, including ^1^H NMR, HPLC, UPPC and MS). d-FF and Cpd-5 were synthetised according to the procedures described in supplemental Information, that also includes identity data. FF, HF and glyburide were obtained from Sigma-Aldrich (purity ≥ 95%, as reported by supplier). Sixteen quinazolinones, having structural similarities to FF, were obtained from ChemBridge (purity ≥ 95%, as reported by supplier). L35, L36, L95, L96, and L97 were provided by Medicines for Malaria Venture (MMV) and characterised as previously described^10^. Amino acids, ATP, AMP, NAD+, resazurin, Phe-Arg beta-naphthylamide dihydrochloride (PAβN), dimethyl sulfoxide (DMSO) and other chemicals were also obtained from Merck-Sigma-Aldrich, whereas TRIzol reagent and *Klostridium kluyveri* diaphorase were acquired from Thermo-Fisher and Worthington Biochemical Corporation, respectively.

### Gene synthesis and plasmid constructs

Synthetic genes with optimised codons for *E. coli* expression were commercially obtained, as follows: the *K. pneumoniae proS* gene (NCBI accession WP_004151928.1), inserted between the *Nde*I and *Xho*I sites of pET28a-TEV vector; *EPRS1* (NCBI gene ID 2058) and *PARS2* (NCBI gene ID 25973), coding for the human cytosolic (PARS1, residues 1013–1522; here referred as HsProRS^cyt^) and mitochondrial (PARS2, residues 41–475; here referred as HsProRS^mit^) isoforms of ProRS, respectively, cloned into the *Nde*I and *Xho*I sites of pET29a; and the *Saccharomyces cerevisiae* AMP deaminase gene (*ScAMD1*, NCBI accession NM_001182392.1), inserted between the *Nde*I and *Xho*I sites of pET28-TEV, were obtained from GeneScript. The *K. pneumoniae* tRNA^Pro^ gene coding for tRNA^Pro^ with the GGC anticodon (GenBank JBAWHZ010000001.1, locus-tag WA40_01075), cloned into the *XbaI* and *XhoI* sites of pET28a, was acquired from SynBio. The DNA sequences of all genes were verified by Sanger DNA sequencing.

### Protein production and purification

HsProRS^cyt^, ScAMD1 and *Campylobacter jejuni* Inosine monophosphate dehydrogenase (CjIMPDH) were expressed and purified as previously described^12^^,^^26^^,^^27^. KpProRS was produced in *E. coli* BL21(DE3) (Invitrogen, cat. n. C600003) at 20 °C, under agitation (200 rpm) in ZYM-5052 autoinduction medium^28^ supplemented with 100 µg/mL kanamycin for 48–72 h. Cells were harvested by centrifugation (6.800 x g for 20 min at 4 °C), suspended in 50 ml of buffer A (50 mM Tris-HCl, 0.3 M NaCl, pH 8.0) containing lysozyme (0.1 mg/mL), DNase I (12.5 µg/mL) and 1 mM PMSF, and ruptured by sonication, on ice. Soluble fractions were separated from the cell debris by centrifugation (40.000 x g) for 1 h at 4 °C, clarified by passages through 0.45 µm Millipore filter devices and subjected to affinity chromatography on a HisTrap-FF 5 ml column (Cytiva), pre-equilibrated with buffer A. The column was washed with 5 column volumes of buffer A, and proteins were eluted in buffer B (50 mM Tris-HCl, 0.3 M NaCl, 0.5 M imidazole, pH 8.0) using an imidazole step gradient of 8, 40 and 100% buffer B with 5 column volumes in each step. Fractions containing KpProRS, verified by SDS-PAGE, were pooled, concentrated and loaded onto a HiLoad Superdex 200 16/600 column (Cytiva). Protein fractions of a single chromatographic peak containing KpProRS were pooled, concentrated at 5–12 mg/mL and kept at −80 °C until use.

### Size exclusion chromatography coupled to light scattering

SEC coupled to fixed Right-Angle and Small-Angle Light Scattering (SEC-RALS/LALS) detectors was performed on a Malvern Panalytical OmniSEC system using a Superdex 200 HR 10/300 GL analytical column (Cytiva) pre-equilibrated with 20 mM Tris-HCl, 0.2 M NaCl, pH 8.0. Initial runs were performed with Bovine serum albumin (BSA, 5 mg/mL) and blue dextran (1 mg/mL), followed by runs using the High Molecular Weight (HMW) gel filtration calibration kit (Cytiva) containing ferritin and conalbumin (mix 1), and ovalbumin and aldolase (mix 2) at 1 mg/mL. Aliquots of 80 µL of KpProRS (5 mg/mL), purified by affinity and size exclusion chromatography, were then loaded onto the column, and two runs with the same protein sample were performed at 18 °C with a flow rate of 0.4 ml/min. Additional runs were performed with the protein at 1 mg/mL. The data were analysed with the OmniSEC system software, considering the calibration standards as molecular weight references.

### RNA preparation

*Escherichia coli* BL21(DE3) cells transformed with pET28a carrying the *K. pneumoniae* tRNA^Pro^ gene were grown under agitation (200 rpm) in Lysogeny Broth (LB) medium supplemented with 50 µg/mL kanamycin, at 37 °C, until the optical density reached 0.6–0.8, when transcription of the tRNA^Pro^ gene was induced with 0.5 mM IPTG for 3 h. The cells were recovered by centrifugation (6,000 x g, 30 min at 4 °C), resuspended in RNase-free water, and total RNA was extracted with TRIzol Reagent, followed by isopropanol/sodium acetate precipitation, according to the manufacturer’s protocol. After centrifugation (15,000 x g, 15 min at 4 °C), RNA pellets were washed three times with 70% ethanol, dried at room temperature and suspended in RNase-free water. The quality and quantity of the RNA samples were analysed by gel electrophoresis and UV spectroscopy.

### Enzyme activity assays

Prolyl-tRNA ligase and proof-reading activities of KpProRS were measured using the enzymatic coupled system described previously, in which resorufin, formed in the presence of AMP derived from the enzymatic catalysis, is detected by fluorescence^27^. The assays were performed in reaction buffer (50 mM Tris-HCl, 0.15 M KCl, 10 mM MgCl2, 0.01% triton X-100, pH 7.5), containing the coupling system (0.25 µM ScAMPD, 200 µM NAD^+^, 0.5 µM CjIMPDH, 25 µM resazurin and 1 U/mL diaphorase). Unless otherwise indicated, 0.4 µg/µL of the bulk *E. coli* RNA containing the *K. pneumoniae* tRNA^Pro^ was added to the reaction mix when measuring the prolyl-tRNA ligase activity. Proof-reading activities were measured for distinct amino acids in absence of RNA. To obtain the kinetics parameters (*K*_M_, *V*_max_ and *k*cat values) of the enzyme, the amino acid (L-Pro or L-Ala) or the nucleotide (ATP) had their concentrations varied while maintaining the other substrate at constant, saturating concentrations. The assays were conducted at 25 °C at different time periods in black 384-well plates, and measurements were taken using a ClarioStar (BMG LabTech) microplate reader with excitation at 545–20 nm and emission at 600–40 nm. Fluorescent signals were converted to amount of AMP formed using an AMP standard curve. The data were analysed with GraphPad Prism software.

### Enzyme inhibition assays and *IC*_50_ determination

Inhibition assays with KpProRS and HsProRS^cyt^ were performed exploring the proof-reading activity of the enzymes and using the same coupling system and reaction buffer described for the enzyme activity assays. For KpProRS inhibition assays, the final concentrations of components were: KpProRS 0.3 µM; L-Ala 60 mM; ATP 2 mM. For HsProRS^cyt^ inhibition assays, the final concentrations of components were: HsProRS^cyt^ 0.25 µM; L-Ala 80 mM; ATP 0.3 mM. Positive controls (100% activity) were made without any inhibitor, but with the same amount of DMSO used in the test samples. Negative controls (0% activity) were made without L-Ala. All assays were performed in black 384-well plates, with a final volume of 25 µL, and measurements were taken on the ClarioStar microplate reader (BMG LabTech). The compounds were prepared in DMSO and transferred to assay plates using an acoustic liquid handler (Echo650, Beckman Coulter). The data obtained were normalised based on controls (positive and negative) and the *IC*_50_ values were obtained by non-linear regression of the data using GraphPad Prism software, according to the equation: Inh = 100/(1 + 10^((log^*^IC^*^50-X)*Hill-slope)^), where Inh is the percentage of inhibition, log*IC*_50_ is the logarithm of the concentration that inhibits 50% of the response, X is the logarithm of the inhibitor concentration, and Hill-slope is the Hill coefficient.

### STD-NMR spectroscopy

Saturation Transfer Difference Nuclear Magnetic Resonance (STD-NMR) were performed by preparing stock solutions (40 mM) of HF, d-HF and derivatives in deuterated DMSO-d6 and mixed with the protein solution in phosphate buffer pH 7.4 to obtain a final solution of 600 μL in deuterated water containing 250 μM of each compound and 1 μM of purified KpProRS. The NMR spectra were obtained on an Agilent DD2 500 MHz spectrometer equipped with a triple resonance cryogenic probe (HCN), operating at 298K. Each STD spectrum was acquired by subtracting the saturated spectrum (on resonance) from the reference spectrum (off-resonance). This was achieved simultaneously by the phase cycle process using a dpfgse_satxfer sequence implemented in the biopack package of the VNMRJ software. STD spectra were obtained with 8192 scans and using selective saturation of the protein signals at −0.5 ppm and 30 ppm for the off-resonance assay. A series of 40 Gaussian pulses (50 ms, with a delay between pulses of 1 ms) was used for a total saturation time of 2.04 s. A T2 filter was applied to eliminate all protein backgrounds. The reference spectrum (off-resonance) was obtained with 4096 scans. The %STD enhancement was calculated by the equation: (*I_0_ - I_sat_*)/(*I_0_*), where (*I_0_ - I_sat_*) is the signal intensity measured directly in the STD-NMR spectrum and *I_0_* is the signal intensity of the unsaturated reference spectrum (off-resonance).

### Minimum inhibitory concentration (MIC) assays

Phenotypic assays for MIC determination of compounds on the growth of *K. pneumoniae* (ATCC BAA-1705 and ATCC BAA-1706), *E. coli* (ATCC 25922 and ATCC 35218), *B. cenocepacia* (ATCC BAA-245) and *S. aureus* (NCTC 12493) were performed using broth microdilution method in sterile 96-well flat bottoms microplates. The final assay volume was 150 μL/well. Inoculums were prepared according to CLSI guidelines. The assay plates were incubated in ovens for 16–24 h under appropriate conditions (37 °C for human pathogens; 28 °C for *B. cenocepacia*; ambient atmosphere). At the end of the incubation period, absorbance at 600 nm was measured using the EnVision microplate reader (PerkinElmer). The absorbance values were normalised using positive controls (100% viability) and negative controls (0% viability), which were prepared using DMSO and tetracycline (32 μg/mL), respectively. MIC values were determined by the minimum concentration where no bacterial growth was observed.

### Checkerboard assays

Except for *B. cenocepacia*, which was tested with PAβN at 16 μg/ml due to its sensitivity above that concentration, all bacterial strains were grown with increasing amounts of Cpd-6 in combination with PAβN at 32 μg/mL - a level that did not affect bacterial growth in studied strains. Bacterial cells were cultured in 96-well plates according to the protocol for MIC determination. The assays were performed in duplicates with four replicates per biological sample. After the incubation period, absorbance at 600 nm was measured and MIC values were determined from the mean values from at least four independent measurements.

### Complementation on *K. pneumoniae ATCC 10031*

*Klebsiella pneumoniae* 10031 competent cells were prepared and electroporated with plasmid *pEcoAB*^29^, essentially as described by Ring et al., 2023^30^. Electroporated cells were plated on Tryptic Soy Agar (TSA) medium containing increasing amounts of tobramycin (0 to 16 µg/ml), and cell colonies capable of growing in 8 µg/ml tobramycin were selected for checkerboard assays.

### Genome-based resistome analyses

Bacterial genomes from NCBI or ATCC databases were uploaded to CARD Resistance Gene Identifier (https://card.mcmaster.ca/analyze/rgi) and ResFinder v.4.7.2 (https://genepi.dk/resfinder) for genotype analysis of ARGs in *K. pneumoniae*, *E. coli*, and *S. aureus* strains using default parameters (perfect, strict and loose hits) with identity of matching regions above 65%. For *B. cenocepacia*, the thresholds for percentage of identity and length of matching sequences were adjusted to 50 and 80, respectively. The ARGs were categorised by gene family and mechanism of resistance.

### Hepatocytes toxicity assays

HepG2 human hepatocarcinoma cells (ATCC HB-8065) were cultured at 37 °C with 5% CO_2_ in high-glucose DMEM (Gibco; Thermo Fisher Scientific) supplemented with 10% foetal bovine serum (Gibco; Thermo Fisher Scientific) and 1% penicillin/streptomycin (Gibco; Thermo Fisher Scientific). Cells were maintained at 70–80% confluence and counted using the trypan blue exclusion method with an automated counter (Countess, Thermo Fisher). For viability assays, passages 4 to 9 were seeded at 2 × 10^4^ cells per well in 96-well plates and incubated for 24 h. Test compounds (d-HF, Cpd-6, and HF) were added at final concentrations of 80, 25, 8, 2.5, and 0.8 μM, with a maximum of 1.6% DMSO as vehicle. Control wells included untreated cells and cells exposed to 1.6% DMSO. After 24 and 48 h of incubation, cell viability was assessed using an MTT assay (Sigma-Aldrich): 20 μL of MTT (5 mg/mL) was added per well, incubated for 4 h, then plates were centrifuged, supernatant discarded, and formazan crystals dissolved in 180 μL DMSO (Hibry-Max, Merck-Sigma-Aldrich). Absorbance was measured at 570 nm using a ClarioStar microplate reader (BMG LabTech).

### Structural modelling and dockings

Structural models of KpProRS and HsProRS^mit^ were generated using AlphaFold2^31^, and with SWISS-MODEL^32^ employing the SaProRS crystal structure (PDB 5ZNJ)^23^ as template. The models were aligned and compared to the crystal structures of human (PDB 4HVC, PDB 7Y1W), *P. falciparum* (PDB 4YDQ) and *S. aureus* (PDB 5ZNJ) ProRS using PyMOL Molecular Graphic System (Schrödinger, LLC). Molecular dockings were performed with AutoDock Vina at the SwissDock^33^^,^^34^.

## Results

### KpProRS exhibits pre-tRNA transfer proofreading and prolyl-tRNA ligase activities

To establish a biochemical baseline for subsequent inhibitor profiling, we first validated the activity and oligomeric state of recombinant KpProRS in solution. We then used tRNA‑dependent and tRNA‑independent assays to distinguish ligase activity from pre‑transfer proofreading.

**Figure 1.**
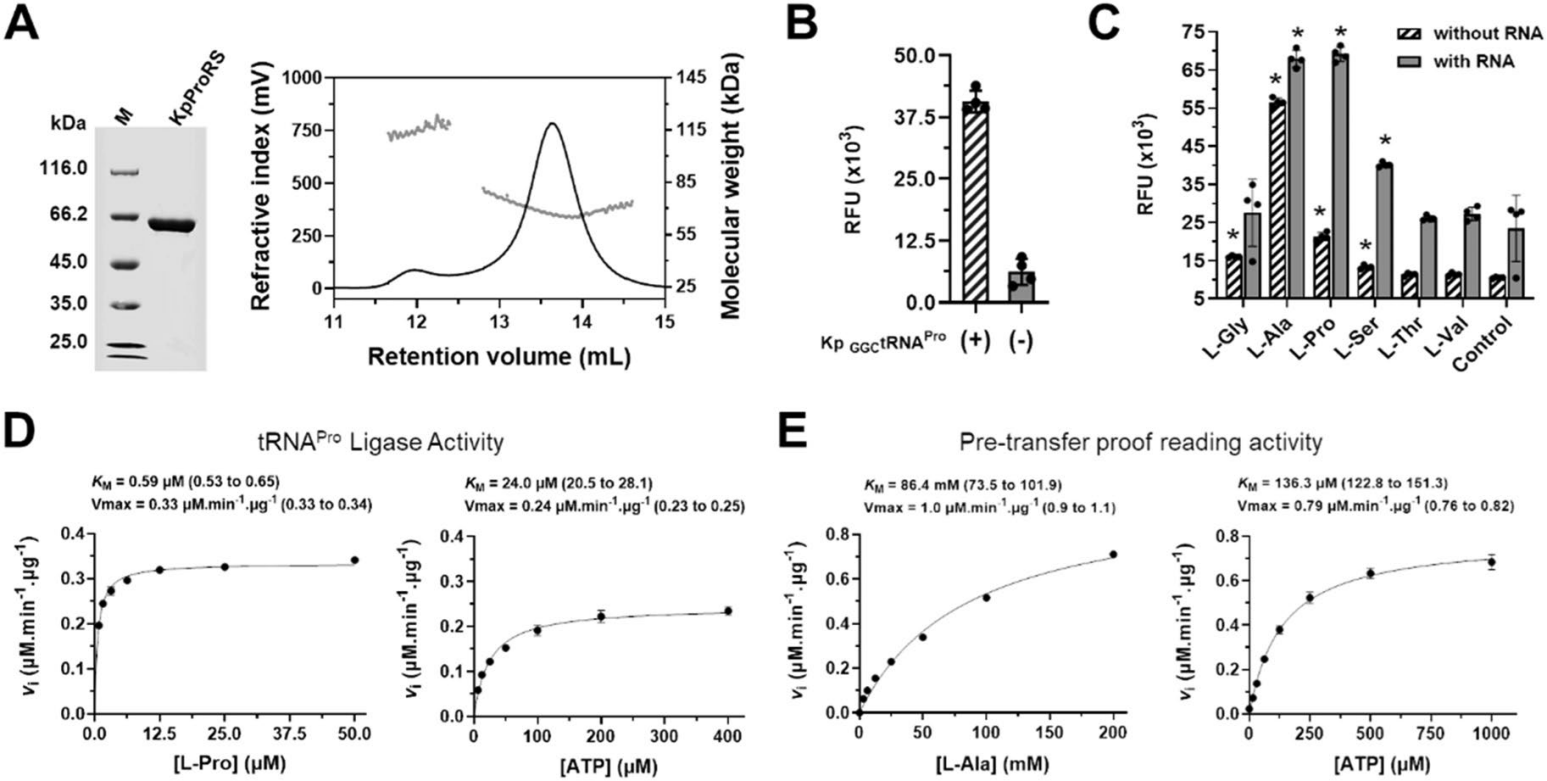
Biochemical characterisation and kinetic properties of KpProRS. (A) SDS-PAGE (left panel) and SEC-RALS/LALS (right panel) analyses of purified KpProRS, showing that recombinant KpProRS is a ∼64 kDa protein that is mainly monomeric in solution. (B) Prolyl-tRNA-ligase activity of KpProRS, as indicated by fluorescence signal after 20 min incubation, measured in the presence of bulk RNA extracted from *E. coli* cells expressing (+) or not (-) the *K. pneumoniae* tRNA^Pro^ gene. (C) tRNA-ligase and proofreading activities of KpProRS measured with or without bulk *E. coli* RNA with *K. pneumoniae* tRNA^Pro^, in the absence (no amino acid control) or presence of 20 mM of L-Gly, L-Ala, L-Pro, L-Ser, L-Thr or L-Val. Fluorescence signal was measured after 2h of incubation. Asterisks indicate statistically significant differences between the means at *p* = 0.05 relative to controls in each condition (with or without RNA). Catalytic parameters of KpProRS obtained in the presence of *K. pneumoniae* tRNA^Pro^, L-Pro and ATP (D) or in the absence of tRNA^Pro^ and presence of L-Ala and ATP (E), representing respectively the tRNA-ligase and pre-transfer proofreading activities.

Recombinant KpProRS was expressed in *E. coli* and purified to homogeneity (Figure 1A). SEC-RALS/LALS analyses revealed that KpProRS exists as both a dimer and a monomer in solution, with the latter being the predominant species in the conditions tested. The monomer had an estimated molecular mass of 64 kDa, matching its predicted molecular size (Figure 1A).

The prolyl-tRNA ligase activity of KpProRS was measured in the absence or presence of bulk RNA extracted from *E. coli* overexpressing the *K. pneumoniae* tRNA^Pro^ gene. In this assay, the AMP released during the tRNA aminoacylation reaction is coupled to resorufin formation, detected by fluorescence^27^^,^^35^. As shown in Figure 1(B), a robust prolyl-tRNA ligase activity was detected particularly when the assay was performed with bulk RNA obtained from *E. coli* cells overexpressing the *K. pneumoniae* tRNA^Pro^, thus confirming the specificity of KpProRS for tRNA^Pro^ acylation.

To evaluate the tRNA-ligase and proof-reading activities of KpProRS, the enzyme activity for six distinct amino acids (L-Gly, L-Ala, L-Pro, L-Ser, L-Thr, and L-Val) was measured in the presence or absence of tRNA^Pro^. L-Cys was not included because it interferes with the AMP detection system used in these assays. As expected, robust tRNA^Pro^-dependent ligase activity was observed with L-Pro. Additionally, in presence of tRNA^Pro^, elevated activities were detected with L-Ser and L-Ala (Figure 1C). Most notably, however, in the absence of *K. pneumoniae* tRNA^Pro^, a strong activity was observed only with L-Ala (Figure 1C). These results indicated that KpProRS exhibits a marked pre-tRNA transfer proofreading activity for L-Ala, which was not observed for L-Ser. Since KpProRS has a cis-editing domain known as insertion domain, it is expected that Ala-tRNA^Pro^ is deacylated releasing free L-Ala and tRNA^Pro^. On the other hand, editing of mischarged Ser-tRNA^Pro^ would require trans-editing factors, such as ProXp-ST1/ST2, which were not present in the assay^36^^,^^37^.

To further examine its kinetic properties, the tRNA-ligase and proofreading activities of KpProRS were measured using various concentrations of L-Pro and tRNA^Pro^ or L-Ala, respectively, in addition to ATP. For the tRNA-ligase activity, KpProRS displayed a *K_m_* of 0.6 µM for L-Pro and 24.0 µM for ATP (Figure 1D), whereas for the pre-tRNA transfer proofreading activity, KpProRS displayed a K_m_ of 86.4 mM for L-Ala and 136.3 µM for ATP (Figure 1E). Thus, the *K_m_* values for L-Pro and ATP obtained for the KpProRS ligase activity are lower than those reported for the *Pseudomonas aeruginosa*, *E. coli*, *Methanocaldococcus jannaschii* and human ProRS, which are in the range of 0.12 to 0.6 mM^19^^,^^26^^,^^38^^,^^39^. Nevertheless, the *K_m_* of KpProRS for L-Ala, as a measure of the proofreading activity, is close to that of the human cytoplasmic (PARS1, hereafter referred to as HsProRS^cyt^) enzyme^26^.

### A novel HF analog with greater potency against KpProRS

Having established robust KpProRS catalytic activity, we next asked whether known ProRS inhibitor scaffolds could inhibit the bacterial enzyme. We then used structure‑guided chemical modifications to improve potency against KpProRS.

**Figure 2.**
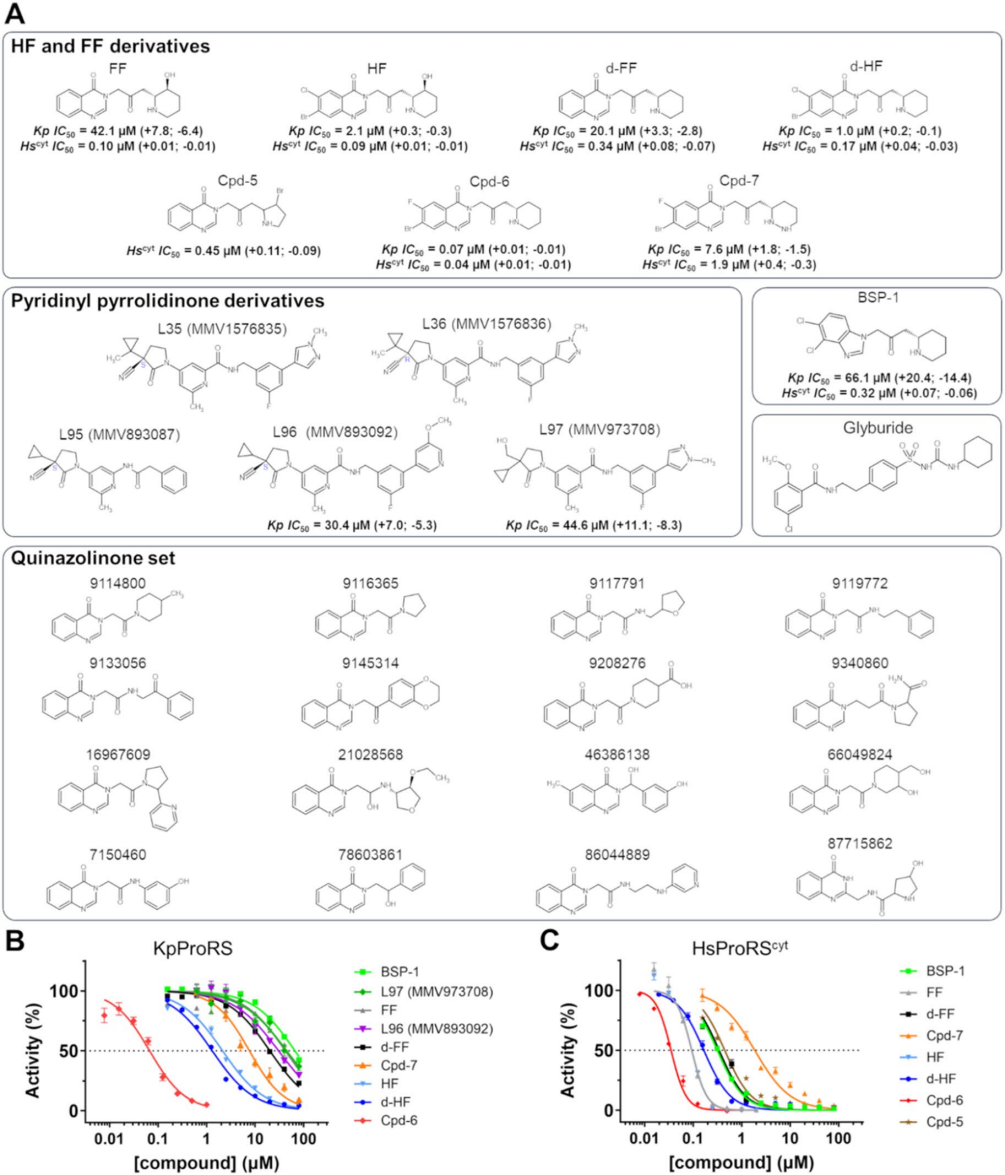
Inhibitory activity assays and potency of HF derivatives and other compounds selected as potential KpProRS inhibitors. (A) Chemical structures and corresponding *IC*_50_ values of the selected ProRS inhibitors tested against KpProRS and HsProRS^cyt^. As the *IC*_50_ values could not be determined for L35, L36, L95, glyburide, and the quinazolinone set, no data is shown. (B) Dose-response curves of compounds that inhibited KpProRS. (C) Dose-response curves of compounds that inhibited HsProRS^cyt^. In the inhibition assays the compounds were analysed at a maximum concentration of 80 µM.

An initial set of 24 molecules representative of known ProRS inhibitors was tested as potential KpProRS inhibitors. In addition to HF and FF, this set of compounds included 16 commercially available quinazolinones (Tanimoto coefficient equal to or greater than 0.8 relative to FF), five MMV ATP-mimetics centred on 1-(pyridin-4-yl) pyrrolidin-2-one scaffold (L35, L36, L95, L96, and L97) originally reported as potent inhibitors of HsProRS^cyt^ and *Toxoplasma gondii* ProRS (TgProRS), and glyburide, an allosteric inhibitor of *Plasmodium falciparum* ProRS (PfProRS) ^10^^,^^40^. From this initial group of compounds, only four demonstrated inhibitory activity against KpProRS. Specifically, FF, L96 and L97 displayed *IC*_50_ values of 42.1, 30.4 and 44.6 µM, respectively, while HF showed low micromolar *IC*_50_ values against KpProRS (Figures 2A and 2B). In contrast, none of the commercially available quinazolinones, nor glyburide, L35, L36, or L95, exhibited a significant effect on KpProRS activity. Consequently, we decided to explore novel HF derivatives as potential KpProRS inhibitors by making substitutions to the original HF structure to achieve higher potency and selectivity towards KpProRS.

An initial set of 24 molecules that are representative of known ProRS inhibitors was tested as potential KpProRS inhibitors. In addition to HF and FF, this set of compounds included 16 commercially available quinazolinones with a Tanimoto coefficient equal to or greater than 0.8 relative to FF, 5 MMV ATP-mimetics centred on 1-(pyridin-4-yl) pyrrolidin-2-one scaffold (L35, L36, L95, L96, and L97) originally reported as potent inhibitors of HsProRS^cyt^ and *Toxoplasma gondii* ProRS (TgProRS), and glyburide, an allosteric inhibitor of *Plasmodium falciparum* ProRS (PfProRS) ^10^^,^^40^. Consequently, we decided to explore novel HF derivatives as potential KpProRS inhibitors by making substitutions to the original HF structure to achieve higher potency and selectivity to KpProRS.

**Figure 3.**
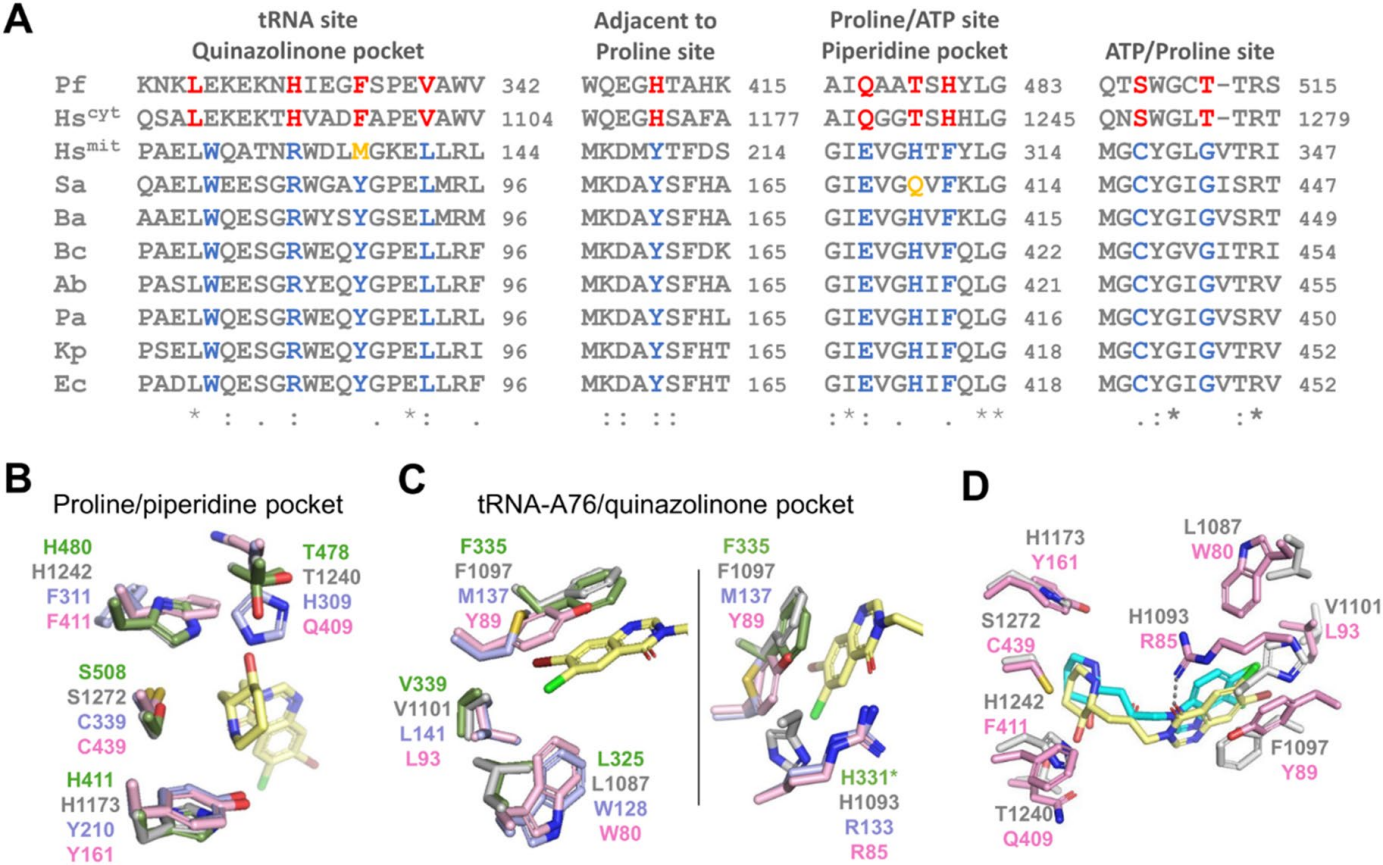
Structure-based design of HF analogs against KpProRS. Protein sequence alignment (A) and superposition of the HsProRS^cyt^ (grey), SaProRS (pink) and PfProRS (green) crystal structures, in complex with HF (yellow, from PDB 4HVC), with the HsProRs^mit^ (blue) structural model, showing amino acid polymorphisms in the piperidine (B) and HF quinazolinone pockets (C, left and right panels) between eukaryotic and prokaryotic ProRS. (D) Structural alignment of the complexes of HsProRS^cyt^ (PDB 7Y1W) and SaProRS, bound respectively to bersiporocin (cyan) and HF (yellow). In the sequence alignment, HF-interacting residues in human (Hs) and *P. falciparum* (Pf) ProRS are shown in red, while interacting residues in SaProRS (Sa) are shown in blue. Additional polymorphism in the piperidine and quinazolinone pockets are marked in orange. In panel C, right panel, H331 of PfProRS is not shown as it is positioned away from the other residues in the structure. Hs^cyt^, human cytosolic ProRS (PDB 4HVC); Pf*, P. falciparum* (PDB 4YDQ); Sa, *S. aureus* (PDB 5ZNJ); Ba, *Bacillus anthracis* (Uniprot C3P5M1); Bc, *Burkholderia cenocepacia* (Uniprot B4E5W6); Ab, *Acinetobacter baumannii* (Uniprot B2HY33); Pa, *Pseudomonas aeruginosa* (PDB 5UCM); Kp, *K. pneumoniae* (Uniprot A6T4Z7) and Ec, *E. coli* (UniProt P16659).

Protein sequence alignment showed several amino acid polymorphisms in the acceptor stem A76, proline and ATP sites between bacterial and eukaryotic ProRS, including the PfProRS, HsProRS^cyt^ and human mitochondrial (HsProRS^mit^) ProRS (Figure 3A). Considering the polymorphisms associated with the proline site (Figure 3B), we first removed the hydroxyl group from the piperidine ring of FF and HF to enhance electrostatic complementarity with F415 in KpProRS, equivalent to F411 in SaProRS, generating deoxy-febrifugine (d-FF) and deoxy-halofuginone (d-HF). Both d-FF and d-HF showed a two-fold increase in potency compared to the original hydroxylated counterparts FF and HF (Figures 2, A and B). In addition, we also replaced the piperidine ring from FF with a pyrrolidine ring with a bromine atom on carbon 3, generating Cpd-5, to investigate whether a halogen at this position would affect interactions with surrounding atoms, particularly F415 in bacterial ProRS (Figure 2A and 3B). This chemical modification nevertheless completely abolished KpProRS inhibition.

Structural comparisons further reveal that, in the A76 site, the change from leucine (L1087) to tryptophan (W80/W128) and from valine (V1101) to leucine (L93/L141) makes the hydrophobic pocket bound by the HF quinazolinone group smaller in bacterial and HsProRS^mit^, relative to HsProRS^cyt^ and PfProRS (Figure 3C). On the other hand, the polymorphism of phenylalanine (F1097) to methionine (M137) or tyrosine (Y89) between HsProRS^cyt^, HsProRS^mit^ and bacterial ProRS, respectively, distinguishes these enzyme variants, because the π-stacking interactions of M137 and Y89 with the HF quinazolinone rings are expected to be less favoured (Figure 3C). Based on these observations, we replaced the chlorine atom of d-HF with fluorine as a substituent on the quinazolinone ring, resulting in compound Cpd-6 (Figure 2A), considering that fluorine would reduce the risk of steric clash with W80 in the bacterial enzyme. Indeed, Cpd-6 displayed an *IC*_50_ of 70 nM, being 30 times more potent than HF and 600 times more potent than FF (Figure 2, A and B). Finally, we replaced the piperidine ring from Cpd-6 with a hexahydro pyridazine ring (Cpd-7) that has a second nitrogen atom (Figure 2A). This modification was designed to evaluate if a second nitrogen atom would be in position to interact with the hydroxyl group of Y161 or sulfhydryl group of C439, two additional polymorphic residues adjacent to the proline site that are present only in prokaryotic ProRS (Figure 3, A and B). Although Cpd-7 was more potent than FF and d-FF, it was much less effective against KpProRS than HF, d-HF and Cpd-6 (Figure 2, A and B), indicating that the hexahydro pyridazine ring adversely affected compound activity. Moreover, because virtual dockings with Cpd-7 show the hexahydro pyridazine ring well fitted into the proline pocket (not shown), we infer that the additional nitrogen inserted into the piperidine ring likely causes unfavourable entropic effects on the enzyme.

Another polymorphism observed in the A76 pocket involves substitution of histidine in HsProRS^cyt^ (H1093) and in PfProRS (H331) with arginine in bacterial enzymes (R85) and HsProRS^mit^ (R133) (Figure 3A). In bacterial enzymes, the guanidinium group of R85 forms a hydrogen bond with the ketone group of the quinazoline ring in HF derivatives (Figure 3C, right panel). Interestingly, the recently reported structure of HsProRS^cyt^ in complex with bersiporocin and some derivatives reveals that these compound bind in same way as HF derivatives, yet they lack a heteroatom at the same position as the ketone on the HF quinazoline ring (Figure 3D) ^16^^,^^17^. Thus, a compound (BSP-1) inspired on bersiporocin derivatives, but lacking a hydroxyl group at the piperidine ring, was planned and evaluated against KpProRS. BSP-1 proved a weak KpProRS inhibitor, with an *IC*_50_ of 66.1 µM (Figure 2A). Since BSP-1 lacks the heteroatom necessary for interaction with R85, these findings suggest that the interaction with the guanidinium group of R85 is a key determinant for binding in bacterial ProRS, directly impacting inhibitors’ potency.

To verify the selectivity of the HF derivatives and BSP-1 towards eukaryotic ProRS compared to KpProRS, we expressed HsProRS^cyt^ and HsProRS^mit^ in *E. coli* cells. However, despite multiple attempts, we could not produce HsProRS^mit^ in the soluble form. HsProRS^cyt^, on the other hand, was purified to homogeneity and inhibited by FF, d-FF, HF and d-HF, which exhibited *IC*_50_ values ranging from 100 to 400 nM (Figure 3C), consistent with previous studies^11^. Notably, d-HF and d-FF showed lower *IC*_50_ values for KpProRS, but higher *IC*_50_ values for HsProRS^cyt^ (Figure 2, B and C), relative to HF and FF, thus supporting our rationale regarding the differential interactions of the piperidine hydroxyl with polymorphic residues at the proline site. Accordingly, Cpd-6, which is distinguished from HF and d-HF by the absence of a hydroxyl group on the piperidine ring and variations in halogen atoms, exhibited greater inhibitory potency against KpProRS but also significantly inhibited HsProRS^cyt^ (*IC*_50_ of 40 ± 10 nM) (Figure 2). These findings indicate that the fluorine atom is well accommodated in the A76 site, as this pocket is larger in HsProRS^cyt^ compared to SaProRS. Finally, BSP-1 proved to be a strong HsProRS^cyt^ inhibitor with low nanomolar *IC*_50_ values (Figure 2C). To our knowledge, BSP-1 is the first bersiporocin derivative with a dehydroxylated piperidine shown to inhibit HsProRS^cyt^.

Taken together, our findings indicate that more potent HF analogs against prokaryotic ProRS can be developed through structure-based design. Nevertheless, further research is needed to develop inhibitors that are more selective for prokaryote enzymes.

### STD‑NMR binding epitope mapping

To clarify the atomic-level interactions between the most potent HF analogues and KpProRS, we employed STD-NMR to map key structural features driving binding affinity. This approach allowed us to rationalise potency differences among derivatives and to further investigate shared enzyme-binding epitopes among HF analogues.

**Figure 4.**
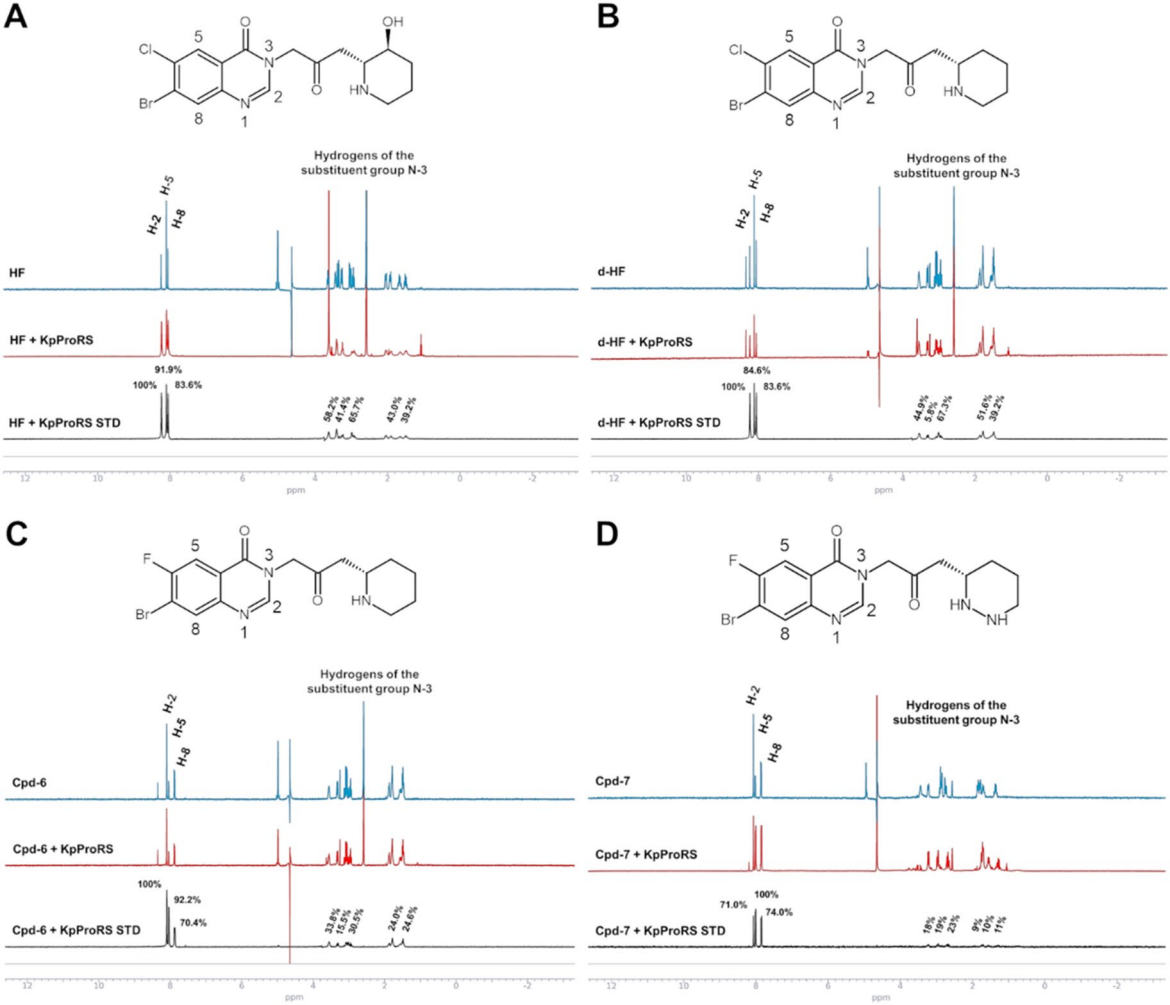
STD-NMR studies reveal that the quinazolinone moiety of HF analogues primarily contributes to the interaction with KpProRS. One-dimension ^1^H-STD NMR assay of HF (A), d-HF (B), Cpd-6 (C) and Cpd-7 (D) in the absence (blue) or presence of KpProRS showing the off-resonance (red) and on-resonance (black) lines. The saturation levels of each hydrogen atom in each of the HF analogs were mapped to their corresponding chemical structures.

We performed ^1^H-STD NMR analyses with HF, d-HF, Cpd-6 and Cpd-7. The results not only confirmed the interaction of the HF analogues with KpProRS, as they produced clear ^1^H-STD signals in the presence of the protein, but also showed that the most intense ^1^H signals correspond to hydrogens belonging to the quinazolinone ring (Figure 4). Thus, the quinazolinone group is likely the main epitope of the ligands, contributing most significantly to their interaction with KpProRS. This is consistent with the observation that chemical modifications within this moiety have a pronounced impact on compound potency. Moreover, the weaker STD signals corresponding to the hydrogens in the hexahydro-pyridazine ring of Cpd-7 (Figure 4D) suggest that nitrogen substitution in this ring substantially reduces its interaction with the enzyme, as previously noted. Under identical experimental conditions, the signal-to-noise ratio in the ^1^H STD-NMR experiments showed that the %STD enhancement for Cpd-7 was approximately 55% lower. This finding explains why Cpd-7 exhibits lower potency compared with HF, d-HF, and Cpd-6 (Figure 2).

### Cpd-6 exhibits antimicrobial activity against critical bacterial pathogens

To determine whether biochemical potency is translated into cellular activity, we evaluated halofuginone derivatives in bacterial growth inhibition assays across a panel of clinically relevant pathogens. Thus, the antimicrobial activity of the HF derivatives was tested against several human bacterial pathogens, including *K. pneumoniae*, reference strains carbapenemase (KPC) producer (ATCC BAA-1705) and carbapenem-sensitive (ATCC BAA-1706), *E. coli*, reference strains *bla^-^* (ATCC 25922) and *bla^+^* (ATCC 35218), MDR *B. cenocepacia* (ATCC BAA-245) and methicillin-resistant (MRSA) *S. aureus* (NCTC 12493). As shown in Table 1, all bacterial strains were resistant or tolerant to FF, d-FF, HF, d-HF, Cpd-5 and Cpd-7, as revealed by the minimum inhibitory concentration (MIC) values. However, *S. aureus* and *E. coli bla^-^* strains were sensitive to Cpd-6, which exhibited MIC values of 2 µg/mL. Cpd-6 also inhibited the growth of *E. coli bla^+^* (MIC 8 µg/mL) and carbapenem-sensitive *K. pneumonia* (MIC 16 µg/mL) but did not kill *K. pneumonia* KPC or MDR *B. cenocepacia* (Table 1). Given that the *IC*_50_ of KpProRS for Cpd-6 is 70 nM (Table 1), the data suggest that a resistance mechanism – such as reduced cell permeability, compound inactivation, or efflux pump activity – may operate in *K. pneumonia* and *B. cenocepacia*.

**Table 1.** Antimicrobial effects of HF derivatives on Gram-negative bacteria and *S. aureus.*

		MIC (µg/mL)					
	*IC*_50_ (µM)	*K. pneumoniae*		*E. coli*		*S. aureus*	*B. cenocepacia*
Compound	KpProRS	ATCC BAA-1705	ATCC BAA-1706	ATCC 25922	ATCC 35218	NCTC 12493	ATCC BAA-245
FF	42.1	> 32	> 32	> 32	> 32	> 32	> 32
d-FF	20.1	> 32	> 32	> 32	> 32	N/A	> 32
HF	2.1	> 32	> 32	> 32	> 32	> 32	> 32
d-HF	1.0	> 32	> 32	32	> 32	16	> 32
Cpd-5	> 80	> 32	> 32	> 32	> 32	> 32	> 32
Cpd-6	0.07	> 128	16	2	8	2	> 128
Cpd-7	7.6	> 128	> 128	> 128	> 128	> 128	> 128
IMP*	N/A	16	4	0,5	1	≤ 0.25	> 32
CFP*	N/A	32	1	≤ 0,25	≤ 0,25	8	> 32

* Imipenem (IMP) and Cefepime (CFP) were used as controls.

Genome-based resistome analyses reveal major contributions of efflux pumps in *K. pneumoniae* and *B. cenocepacia* antibiotic resistance.

Because KpProRS inhibition did not uniformly translated into activity against MDR Gram‑negative strains, we next explored genomic resistance determinants that could limit compound accumulation. We therefore used genome‑based resistome profiling to identify candidate mechanisms consistent with the phenotypic data.

The BAA-1705 strain of *K. pneumonia* KPC was isolated from human urine and is largely used as a positive control for carbapenemase production^41^, whereas *B cenocepacia* BAA-245 is a clinical specimen isolated from a cystic fibrosis patient^42^ that is used as a type-strain and quality control for pharmaceutical testing. To better understand the molecular basis of the drug resistance phenotype displayed by these pathogens, we analysed their genome-based resistome using CARD and ResFinder^43^^,^^44^.

In *K. pneumoniae*, we identified 58 antibiotic resistance genes (ARGs) and their respective resistance mechanisms. Among them, 34 genes are related to efflux pump activity, 16 are associated with drug inactivation, 5 are linked to target modification or replacement, and 3 are related to reduced cell permeability (Supplemental Table 1). In *B. cenocepacia*, we identified 38 ARGs, including 27 efflux pump genes, 9 antibiotic inactivation genes, 1 target alteration gene, and 1 porin gene (Supplemental Table 1). These analyses, therefore, highlight the preponderant role of efflux pump in the drug resistance phenotype displayed by these bacterial pathogens.

Because our genome-based resistome analyses indicated that strains BAA-1705 and BAA-245 use efflux pumps and drug inactivation systems as key antibiotic resistance mechanisms, we decided to also assess the resistome of the other bacterial strains shown in Table 1, as they responded differently to the KpProRS inhibitors. Again, using CARD and ResFinder, we found 137 ARGs distributed among the strains (Supplemental Table 1). As observed for BAA-1705, the major categories of resistance determinants found in all bacterial genomes inspected were associated with enhanced efflux pump activity (87 genes), antibiotic inactivation (35 genes), target alteration (11 genes), followed by reduced porin activity (4 genes) (Supplemental Table 1). The main efflux pumps belong to the RND (34 genes) and MFS (26 genes) protein families, whereas the main drug inactivation enzymes are beta-lactamases and transferases that inactivate or modify beta-lactams, aminoglycosides and macrolides (Supplemental Table 1).

### Efflux pump inhibitor PAβN potentiates cpd-6 efficacy against *K. pneumoniae*

To functionally assess whether RND-type efflux limits intracellular Cpd‑6 accumulation, we performed checkerboard assays in the presence of the competitive RND inhibitor PAβN.

The ARG patterns observed in Cpd-6 sensitive and resistant strains suggested that increased efflux pump activity or reduced drug permeation, rather than drug inactivation or target modification, is likely causing Cpd-6 resistance in *K. pneumoniae* BAA-1705/1706 and *B. cenocepacia* (Supplemental Table 1). For instance, the RND *adeF* and *oqxA,* and the MSF *knpGH* genes, which confer multidrug resistance in many *Enterobacteriaceae*^45–48^, were particularly found in the *K. pneumoniae* and *B. cenocepacia* strains (Supplemental Table 1).

**Figure 5.**
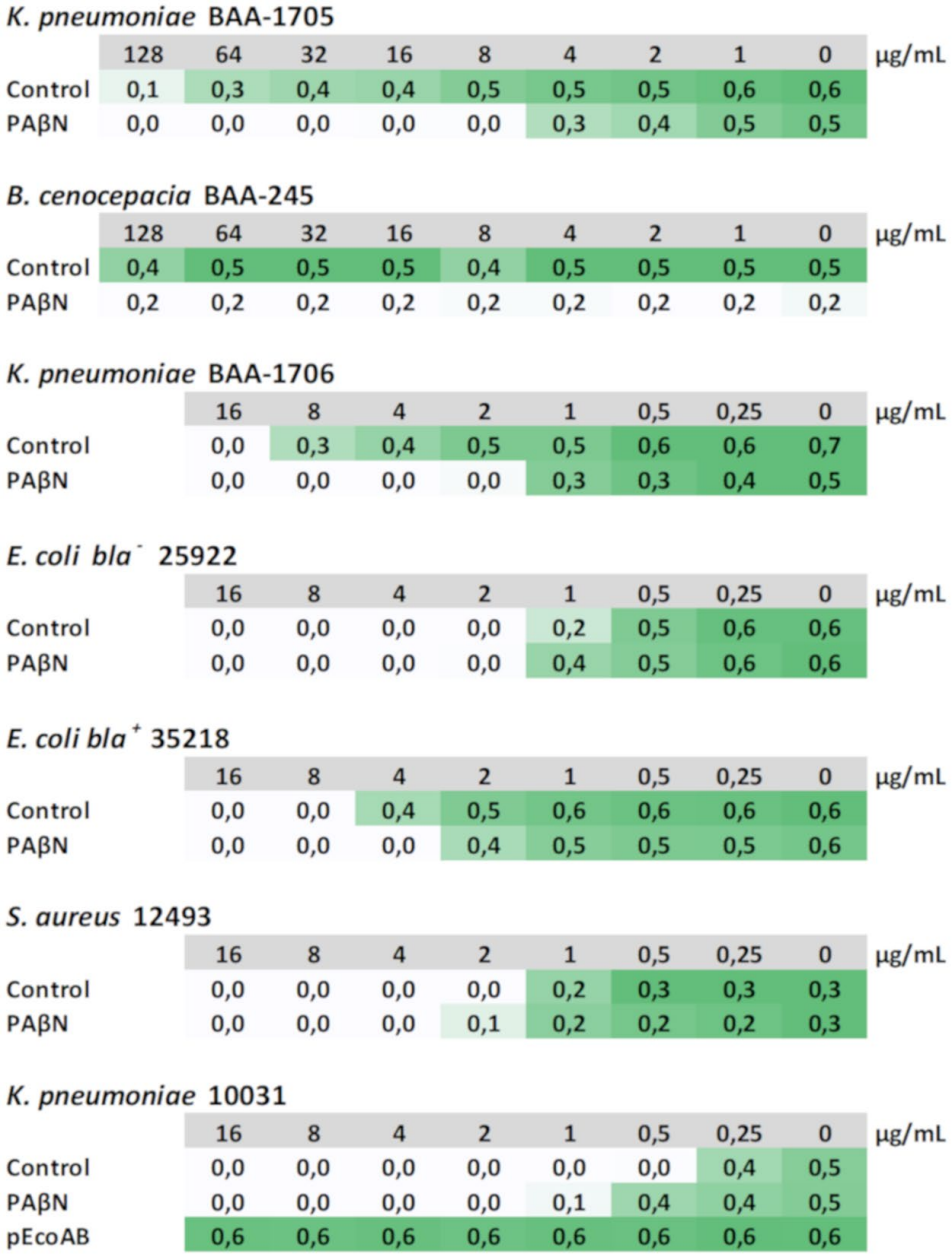
Checkerboard analysis of the effect of the efflux pump inhibitor PAβN on Cpd-6 activity against bacterial pathogens. The colour grade is relative to absorbance values of bacterial growth cultures, from white (minimum or no growth) to dark green (maximum growth). Absorbance values are the means of at least four independent measurements. Cpd-6 concentration representing MIC values in µg/mL are indicated in grey. PAβN was used at 32 µg/mL for all bacterial pathogens, except *B. cenocepacia* where it was used at the maximum tolerable concentration of 16 µg/mL.

To assess the role of RND efflux pumps in Cpd-6 resistance, we used PAβN, a competitive inhibitor of substrate binding of RND family efflux pumps^49^. Our checkerboard assays showed that PAβN at 32 µg/mL, significantly reduced the MIC values of Cpd-6 from more than 128 to 8 µg/mL and from 16 to 2 µg/mL in *K. pneumoniae* BAA-1705 and BAA-1706, respectively (Figure 5). PAβN slightly reduced the MIC of Cpd-6 in *E. coli bla^+^* from 8 to 4 µg/mL but did not change the Cpd-6 MIC in *E. coli bla^-^* or in *S. aureus* (Figure 5), which aligns with the fact that RND efflux pumps are typically absent in Gram-positive bacteria^49^^,^^50^. PAβN at 16 µg/mL slightly inhibited *B. cenocepacia* growth but did not enhance Cpd-6 activity (Figure 5). These results therefore indicate that RND efflux pumps significantly contribute to Cpd-6 susceptibility in *K. pneumoniae.*

### The *AcrAB* efflux pump is crucial for Cpd-6 resistance in *K. pneumoniae*

The data above, including genome analysis and pharmacological modulation with PAβN, strongly indicate that RND family efflux pumps likely play a role in Cpd-6 resistance in *K. pneumonia*. To verify this assumption and confirm that resistance to Cpd-6 in *K. pneumoniae* is dependent on the activity of RND efflux pumps, more specifically on *acrAB* efflux system, checkerboard assays were conducted with *K. pneumoniae* strain ATCC 10031. This strain is hypersensitive to many antibiotics due to a premature stop codon in the *acrB* gene, which encodes the *AcrB* component of the AcrAB efflux pump^51^^,^^52^. As anticipated, the *K. pneumoniae acrB* mutant exhibited increased sensitivity to Cpd-6 (MIC of 0.5 µg/mL) compared to the BAA-1705 and BAA-1706 strains (Figure 5). Importantly, the MIC of Cpd-6 in the *acrB* mutant was not significantly affected by PAβN (Figure 5), indicating that the *AcrAB* pump plays a major role in Cpd-6 resistance in *K. pneumoniae*. To confirm this idea, the 10031 strain was complemented with plasmid *pEcoAB* carrying the *E. coli acrA* and *acrB* genes^29^. As expected, the complemented mutant gained resistance to Cpd-6, as indicated by MIC values (Figure 5).

Taking together, our results show that resistance to Cpd-6 in *K. pneumonia* is largely, if not exclusively, mediated by the *AcrAB* efflux pump system.

### Cpd-6 displays toxicity to human hepatocytes

**Figure 6.**
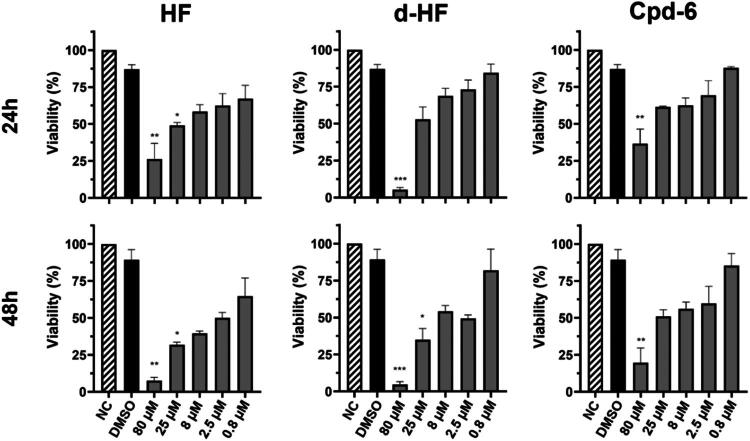
Assessment of cell toxicity in hepatocytes. HepG2 cell viability was measured after 24h and 48h exposure to various concentrations of HF, d-HF, and Cpd-6. Data was obtained using the MTT assay methodology, and experiments were conducted with 4 technical replicates. P values of < 0.033 (*), < 0.002 (**), and < 0.001 (***) indicate statistically significant differences compared to the DMSO control, as determined by the Kruskal-Wallis test.

Since Cpd-6 inhibited HsProRS^cyt^ with higher efficiency than HF and d-HF, we decided to evaluate its toxicity in human hepatocytes (HepG2 cell line), compared to HF and d-HF. HepG2 cells exhibited good tolerance to HF, d-HF and Cpd-6, relative to controls (with or without DMSO) at concentrations equal to or below 25 µM for a period of 24h of growth. When cells were exposed to the drugs for 48h, HF and d-HF showed higher toxicity, while Cpd-6 continued to be relatively tolerated in concentration going up to 25 µM. Importantly, at a final concentration of 80 µM, HF, d-HF and Cpd-6 showed important cytotoxicity as early as 24 h of treatment (Figure 6). These results therefore indicate that Cpd-6 retains a measurable therapeutic window *in vitro*, while underscoring the importance of refining selectivity in future analogues.

## Discussion

Antimicrobial resistance is a serious global health problem that makes bacterial infections harder to treat and, in most cases, potentially life-threatening. The development of new antibiotics is thus crucial to providing alternative or more effective treatment options for MDR bacteria.

Here, we present the biochemical properties of KpProRS and show data that reinforce this enzyme as a promising antimicrobial target in MDR *K. pneumoniae*, as it is in other critical bacterial pathogens, like *E. coli* and *S. aureus*. From an initial set of 24 molecules that are representative of the known ProRS inhibitors described to date, we found HF as an effective KpProRS inhibitor. Given that ProRS from eukaryotes are also inhibited by HF and its derivatives, we employed a structure-based approach to explore the amino acid polymorphisms that exist in the active sites of ProRS between prokaryotic and eukaryotic enzymes to design new ProRS inhibitors with enhanced potency against bacterial ProRS, as most of the commercially available HF analogues and ProRS inhibitors against eukaryotic ProRS failed to efficiently inhibit KpProRS. Most significantly, Cpd-6 exhibited *IC*_50_ values in nanomolar range against KpProRS and inhibited the growth of *S. aureus* MRSA at 2 µg/mL, whereas in combination with PAβN, also inhibited the growth of MDR *K. pneumoniae*. Although this approach allowed for chemical modifications to the HF molecule that significantly increased efficiency against KpProRS, such as with Cpd-6, this molecule also demonstrated equivalent potency against the human enzyme. Additionally, Cpd-6 exhibited moderate toxicity to human hepatocytes, likely because of HsProRS^cyt^ inhibition. These findings emphasise therefore the need to further exploit the structural differences between eukaryotic and prokaryotic ProRS to develop more selective inhibitors for bacterial enzymes. This is particularly crucial because strong inhibition of the human enzyme could cause unwanted side effects in tissues with high protein turnover demands. Nevertheless, despite our efforts are in line with recent reports identifying ProRS from *S. aureus* and *P. aeruginosa* as promising targets for novel antibiotics development^23^^,^^53^, it remains to be determined whether potent ProRS inhibitors with encouraging MIC values can translate into efficacy in animal models.

Most of the new antibiotics approved for clinical use in recent years are beta-lactamase inhibitors, fluoroquinolone and tetracycline derivatives, and aminoglycoside conjugates^20^^,^^22^^,^^54^. Thus, it is essential that new drugs with different mechanisms of action are developed to combat bacteria, particularly Gram-negative MDR pathogens.

Combining antibiotics with different mechanisms of action is also key for improving treatment effectiveness through synergistic interactions, especially considering that resistance development to synergistic combinations does not necessarily lead to cross-resistance to the individual antibiotics used in the combination^55^^,^^56^. However, due to the pharmacological complexity of dual or triple combination treatments and the scarcity of studies to demonstrate their efficacy in animal models, only a few antibiotic combination treatments have been introduced in clinical settings in recent decades. For instance, treatment options for carbapenem-resistant Gram-negative bacteria include combinations of beta-lactam antibiotics and beta-lactamase inhibitors, the novel siderophore cephalosporin cefiderocol, as well as combinations with polymyxins and tigecycline^20^^,^^21^^,^^54^^,^^56^^,^^57^. Therefore, our findings that Cpd-6 combined with PAβN is effective against MDR bacteria hold great promise as a mechanistic demonstration that efflux can dominate cellular susceptibility even when ProRS is strongly inhibited.

Although several natural and synthetic EPIs significantly enhance antibiotic efficacy by targeting bacterial efflux pumps, none have yet been integrated into clinical practice, mainly due to toxicity and undesirable side effects^22^. In this context, it is important to note that PAβN is widely used as a research tool but is not clinically viable due to toxicity and suboptimal pharmacokinetics. Despite PAβN could not be used in combination therapies due to its toxicity in mammalian cells, its safe use in ducks at 40 µg/g of body weight has recently been reported to markedly enhance neomycin sensitivity to *Riemerella anatipestifer*^58^. Nevertheless, the growing knowledge of the three-dimensional structures of efflux pumps is expected to facilitate the design of new EPIs with improved selectivity and reduced toxicity in humans.

In conclusion, our findings demonstrate that the *AcrAB* efflux pump plays a pivotal role in mediating resistance to Cpd-6 in *K. pneumoniae*, thereby further expanding the validity of ProRS enzyme as an antimicrobial target and underscoring the significance of efflux-based resistance mechanisms to halofuginone-derived quinazolinone compounds. While the data supports the potential of targeting efflux pumps to enhance the activity of halofuginone derivatives, the clinical applicability of combining these ProRS inhibitors with efflux pump inhibitors (EPIs) to combat MDR bacteria remains to be established. Future studies should focus on designing selective ProRS inhibitors, while improved RND EPIs with broad applicability alongside other antibiotics might also be developed.

## Supplementary Material

Supporting_Information_anonymous-cl.docx

Supplemental_Table1_revised.xlsx

## Data Availability

The data that supports the findings of this study, if not in the manuscript or supplemental material, are available from the corresponding authors, upon reasonable request.

## References

[1] Gomez MAR, Ibba M. Aminoacyl-tRNA synthetases. RNA. 2020;26(8):910–936. 32303649 10.1261/rna.071720.119 PMC7373986

[2] Hu Y, Guerrero E, Keniry M, Manrrique J, Bullard JM. Identification of chemical compounds that inhibit the function of glutamyl-tRNA synthetase from pseudomonas aeruginosa. J Biomol Screen. 2015;20(9):1160–1170. 26116192 10.1177/1087057115591120 PMC4575845

[3] Williams TL, Yin YW, Carter CW. Selective inhibition of bacterial tryptophanyl-tRNA synthetases by indolmycin is mechanism-based. J Biol Chem. 2016;291(1):255–265. 26555258 10.1074/jbc.M115.690321 PMC4697160

[4] Zhang P, Ma S. Recent development of leucyl-tRNA synthetase inhibitors as antimicrobial agents. Medchemcomm. 2019;10(8):1329–1341. 31534653 10.1039/c9md00139e PMC6727470

[5] Guo J, Chen B, Yu Y, Cheng B, Cheng Y, Ju Y, Gu Q, Xu J, Zhou H. Discovery of novel tRNA-amino acid dual-site inhibitors against threonyl-tRNA synthetase by fragment-based target hopping. Eur J Med Chem. 2020;187:111941. 31821989 10.1016/j.ejmech.2019.111941

[6] Xie SC, Griffin MDW, Winzeler EA, Ribas De Pouplana L, Tilley L. Targeting Aminoacyl tRNA Synthetases for Antimalarial Drug Development. Annu Rev Microbiol. 2023;77(1):111–129. 37018842 10.1146/annurev-micro-032421-121210

[7] Kelly P, Hadi-Nezhad F, Liu DY, Lawrence TJ, Linington RG, Ibba M, Ardell DH. Targeting tRNA-synthetase interactions towards novel therapeutic discovery against eukaryotic pathogens. PLoS Negl Trop Dis. 2020;14(2):e0007983. 32106219 10.1371/journal.pntd.0007983 PMC7046186

[8] Jain V, Yogavel M, Oshima Y, Kikuchi H, Touquet B, Hakimi MA, Sharma A. Structure of prolyl-tRNA synthetase-halofuginone complex provides basis for development of drugs against malaria and toxoplasmosis. Structure. 2015;23(5):819–829. 25817387 10.1016/j.str.2015.02.011

[9] Jain V, Yogavel M, Kikuchi H, Oshima Y, Hariguchi N, Matsumoto M, Goel P, Touquet B, Jumani RS, Tacchini-Cottier F, et al. Targeting Prolyl-tRNA Synthetase to Accelerate Drug Discovery against Malaria, Leishmaniasis, Toxoplasmosis, Cryptosporidiosis, and Coccidiosis. Structure. 2017;25(10):1495–1505.e6. 28867614 10.1016/j.str.2017.07.015

[10] Yogavel M, Bougdour A, Mishra S, Malhotra N, Chhibber-Goel J, Bellini V, Harlos K, Laleu B, Hakimi MA, Sharma A. Targeting prolyl-tRNA synthetase via a series of ATP-mimetics to accelerate drug discovery against toxoplasmosis. PLoS Pathog. 2023;19(2):e1011124. 36854028 10.1371/journal.ppat.1011124 PMC9974123

[11] Keller TL, Zocco D, Sundrud MS, Hendrick M, Edenius M, Yum J, Kim YJ, Lee HK, Cortese JF, Wirth DF, et al. Halofuginone and other febrifugine derivatives inhibit prolyl-tRNA synthetase. Nat Chem Biol. 2012;8(3):311–317. 22327401 10.1038/nchembio.790 PMC3281520

[12] Zhou H, Sun L, Yang XL, Schimmel P. ATP-directed capture of bioactive herbal-based medicine on human tRNA synthetase. Nature. 2013;494(7435):121–124. 23263184 10.1038/nature11774 PMC3569068

[13] Shibata A, Kuno M, Adachi R, Sato Y, Hattori H, Matsuda A, Okuzono Y, Igaki K, Tominari Y, Takagi T, et al. Discovery and pharmacological characterization of a new class of prolyl-tRNA synthetase inhibitor for anti-fibrosis therapy. PLoS One. 2017;12(10):e0186587. 29065190 10.1371/journal.pone.0186587 PMC5655428

[14] Kim SH, Bae S, Song M. Recent development of aminoacyl-trna synthetase inhibitors for human diseases: A future perspective. Biomolecules. 2020;10(12):1625. 33271945 10.3390/biom10121625 PMC7760260

[15] Kurata K, Bott AJ, Tye MA, Yamamoto L, Samur MK, Tai YT, Dunford J, Johansson C, Senbabaoglu F, Philpott M, et al. Prolyl-tRNA synthetase as a novel therapeutic target in multiple myeloma. Blood Cancer J. 2023;13(1):24–890. 36746923 10.1038/s41408-023-00793-y PMC9902474

[16] Yoon I, Kim S, Cho M, You KA, Son J, Lee C, Suh JH, Bae D, Kim JM, Oh S, et al. Control of fibrosis with enhanced safety via asymmetric inhibition of prolyl-tRNA synthetase 1. EMBO Mol Med. 2023;15(7):e16940. 37212275 10.15252/emmm.202216940 PMC10331583

[17] Park MY, Bae S, Heo JA, Park M, Kim YK, Han J, Jang IJ, Yu KS, Oh J. Safety, tolerability, pharmacokinetic/pharmacodynamic characteristics of bersiporocin, a novel prolyl-tRNA synthetase inhibitor, in healthy subjects. Clin Transl Sci. 2023;16(7):1163–1176. 37095713 10.1111/cts.13518 PMC10339703

[18] Crepin T, Yaremchuk A, Tukalo M, Cusack S. Structures of two bacterial prolyl-tRNA synthetases with and without a cis-editing domain. Structure. 2006;14(10):1511–1525. 17027500 10.1016/j.str.2006.08.007

[19] Pena N, Dranow DM, Hu Y, Escamilla Y, Bullard JM. Characterization and structure determination of prolyl-tRNA synthetase from Pseudomonas aeruginosa and development as a screening platform. Protein Sci. 2019;28(4):727–737. 30666738 10.1002/pro.3579 PMC6423717

[20] Gajic I, Tomic N, Lukovic B, Jovicevic M, Kekic D, Petrovic M, Jankovic M, Trudic A, Mitic Culafic D, Milenkovic M, et al. A comprehensive overview of antibacterial agents for combating multidrug-resistant bacteria: the current landscape, development, future opportunities, and challenges. Antibiotics (Basel). 2025;14(3):221. 40149033 10.3390/antibiotics14030221 PMC11939824

[21] Murugaiyan J, Anand Kumar P, Rao GS, Iskandar K, Hawser S, Hays JP, Mohsen Y, Adukkadukkam S, Awuah WA, Jose RAM, et al. Progress in alternative strategies to combat antimicrobial resistance: focus on antibiotics. Antibiotics (Basel). 2022;11(2):200. 35203804 10.3390/antibiotics11020200 PMC8868457

[22] Khan RT, Sharma V, Khan SS, Rasool S. Prevention and potential remedies for antibiotic resistance: current research and future prospects. Front Microbiol. 2024;15:1455759. 39421555 10.3389/fmicb.2024.1455759 PMC11484029

[23] Cheng B, Cai Z, Luo Z, Luo S, Luo Z, Cheng Y, Yu Y, Guo J, Ju Y, Gu Q, et al. Structure-guided design of halofuginone derivatives as ATP-aided inhibitors against bacterial prolyl-tRNA synthetase. J Med Chem. 2022;65(23):15840–15855. 36394909 10.1021/acs.jmedchem.2c01496

[24] Poerio N, Olimpieri T, Henrici De Angelis L, De Santis F, Thaller MC, D’Andrea MM, Fraziano M. Fighting MDR-*Klebsiella pneumoniae* infections by a combined host- and pathogen-directed therapeutic approach. Front Immunol. 2022;13:835417. 35237274 10.3389/fimmu.2022.835417 PMC8884248

[25] Murray CJ, Ikuta KS, Sharara F, Swetschinski L, Robles Aguilar G, Gray A, Han C, Bisignano C, Rao P, Wool E, et al. Global burden of bacterial antimicrobial resistance in 2019: a systematic analysis. The Lancet. 2022;399(10325):629–655. 10.1016/S0140-6736(21)02724-0 PMC8841637 35065702

[26] Beuning PJ, Musier-Forsyth K. Species-specific differences in amino acid editing by Class II Prolyl-tRNA synthetase. J Biol Chem. 2001;276(33):30779–30785. 11408489 10.1074/jbc.M104761200

[27] Mercaldi GF, de Oliveira Andrade M, de Lima Zanella J, Cordeiro AT, Benedetti CE. Molecular basis for diaryldiamine selectivity and competition with tRNA in a type 2 methionyl-tRNA synthetase from a gram-negative bacterium. J Biol Chem. 2021;296:100658. 33857480 10.1016/j.jbc.2021.100658 PMC8165550

[28] Studier FW. Protein production by auto-induction in high density shaking cultures. Protein Expr Purif. 2005;41(1):207–234. 15915565 10.1016/j.pep.2005.01.016

[29] Bharatham N, Bhowmik P, Aoki M, Okada U, Sharma S, Yamashita E, Shanbhag AP, Rajagopal S, Thomas T, Sarma M, et al. Structure and function relationship of OqxB efflux pump from Klebsiella pneumoniae. Nat Commun. 2021;12(1):5400. 34518546 10.1038/s41467-021-25679-0 PMC8437966

[30] Ring BE, Khadka S, Pariseau DA, Mike LA. Genetic manipulation of *Klebsiella pneumoniae*. Curr Protoc. 2023;3(10):e912. 37889096 10.1002/cpz1.912 PMC10617658

[31] Jumper J, Evans R, Pritzel A, Green T, Figurnov M, Ronneberger O, Tunyasuvunakool K, Bates R, Žídek A, Potapenko A, et al. Highly accurate protein structure prediction with AlphaFold. Nature. 2021;596(7873):583–589. 34265844 10.1038/s41586-021-03819-2 PMC8371605

[32] Waterhouse A, Bertoni M, Bienert S, Studer G, Tauriello G, Gumienny R, Heer FT, de Beer TAP, Rempfer C, Bordoli L, et al. SWISS-MODEL: homology modelling of protein structures and complexes. Nucleic Acids Res. 2018;46(W1):W296–W303. 29788355 10.1093/nar/gky427 PMC6030848

[33] Bugnon M, Röhrig UF, Goullieux M, Perez MAS, Daina A, Michielin O, Zoete V. SwissDock 2024: major enhancements for small-molecule docking with attracting cavities and AutoDock Vina. Nucleic Acids Res. 2024;52(W1):W324–W332. 38686803 10.1093/nar/gkae300 PMC11223881

[34] Eberhardt J, Santos-Martins D, Tillack AF, Forli S. AutoDock Vina 1.2.0: new docking methods, expanded force field, and python bindings. J Chem Inf Model. 2021;61(8):3891–3898. 34278794 10.1021/acs.jcim.1c00203 PMC10683950

[35] First EA, Richardson CJ. Spectrophotometric assays for monitoring tRNA aminoacylation and aminoacyl-tRNA hydrolysis reactions. Methods. 2017;113:3–12. 27780756 10.1016/j.ymeth.2016.10.010

[36] Kumar S, Das M, Hadad CM, Musier-Forsyth K. Substrate specificity of bacterial prolyl-tRNA synthetase editing domain is controlled by a tunable hydrophobic pocket. J Biol Chem. 2012;287(5):3175–3184. 22128149 10.1074/jbc.M111.313619 PMC3270972

[37] Cruz E, Vargas-Rodriguez O. The role of tRNA identity elements in aminoacyl-tRNA editing. Front Microbiol. 2024;15:1437528. 39101037 10.3389/fmicb.2024.1437528 PMC11295145

[38] Norton SJ. Purification and properties of the prolyl RNA synthetase of Escherichia coli. Arch Biochem Biophys. 1964;106(C):147–152. 14217147 10.1016/0003-9861(64)90167-5

[39] Johnson JM, Sanford BL, Strom AM, Tadayon SN, Lehman BP, Zirbes AM, Bhattacharyya S, Musier-Forsyth K, Hati S. Multiple pathways promote dynamical coupling between catalytic domains in Escherichia coli prolyl-tRNA synthetase. Biochemistry. 2013;52(25):4399–4412. 23731272 10.1021/bi400079h PMC3749879

[40] Hewitt SN, Dranow DM, Horst BG, Abendroth JA, Forte B, Hallyburton I, Jansen C, Baragaña B, Choi R, Rivas KL, et al. Biochemical and structural characterization of selective allosteric inhibitors of the Plasmodium falciparum drug target, prolyl-tRNA-synthetase. ACS Infect Dis. 2017;3(1):34–44. 27798837 10.1021/acsinfecdis.6b00078 PMC5241706

[41] Mohammadpour D, Memar MY, Leylabadlo HE, Ghotaslou A, Ghotaslou R. Carbapenem-Resistant Klebsiella pneumoniae: A comprehensive review of phenotypic and genotypic methods for detection. Microbe (Netherlands). 2025;6:100246.

[42] Vandamme P, Holmes B, Vancanneyt M, Coenye T, Hoste B, Coopman R, Revets H, Lauwers S, Gillis M, Kersters K, et al. Occurrence of multiple genomovars of Burkholderia cepacia in cystic fibrosis patients and proposal of Burkholderia multivorans sp. nov. Int J Syst Bacteriol. 1997;47(4):1188–1200. 9336927 10.1099/00207713-47-4-1188

[43] Bortolaia V, Kaas RS, Ruppe E, Roberts MC, Schwarz S, Cattoir V, Philippon A, Allesoe RL, Rebelo AR, Florensa AF, et al. ResFinder 4.0 for predictions of phenotypes from genotypes. J Antimicrob Chemother. 2020;75(12):3491–3500. 32780112 10.1093/jac/dkaa345 PMC7662176

[44] Alcock BP, Huynh W, Chalil R, Smith KW, Raphenya AR, Wlodarski MA, Edalatmand A, Petkau A, Syed SA, Tsang KK, et al. CARD 2023: expanded curation, support for machine learning, and resistome prediction at the Comprehensive Antibiotic Resistance Database. Nucleic Acids Res. 2023;51(D1):D690–D699. 36263822 10.1093/nar/gkac920 PMC9825576

[45] Coyne S, Rosenfeld N, Lambert T, Courvalin P, Périchon B. Overexpression of resistance-nodulation-cell division pump AdeFGH confers multidrug resistance in Acinetobacter baumannii. Antimicrob Agents Chemother. 2010;54(10):4389–4393. 20696879 10.1128/AAC.00155-10 PMC2944555

[46] Li J, Zhang H, Ning J, Sajid A, Cheng G, Yuan Z, Hao H. The nature and epidemiology of OqxAB, a multidrug efflux pump. Antimicrob Resist Infect Control. 2019;8(1):44. 30834112 10.1186/s13756-019-0489-3 PMC6387526

[47] Srinivasan VB, Rajamohan G. KpnEF, a new member of the *Klebsiella pneumoniae* cell envelope stress response regulon, is an SMR-type efflux pump involved in broad-spectrum antimicrobial resistance. Antimicrob Agents Chemother. 2013;57(9):4449–4462. 23836167 10.1128/AAC.02284-12 PMC3754300

[48] Srinivasan VB, Singh BB, Priyadarshi N, Chauhan NK, Rajamohan G. Role of novel multidrug efflux pump involved in drug resistance in *Klebsiella pneumoniae*. PLoS One. 2014;9(5):e96288. 24823362 10.1371/journal.pone.0096288 PMC4019481

[49] Compagne N, Vieira Da Cruz A, Müller RT, Hartkoorn RC, Flipo M, Pos KM. Update on the discovery of efflux pump inhibitors against critical priority Gram-negative bacteria. Antibiotics (Basel). 2023;12(1):180. 36671381 10.3390/antibiotics12010180 PMC9854755

[50] Schindler BD, Kaatz GW. Multidrug efflux pumps of Gram-positive bacteria. Drug Resist Updat. 2016;27:1–13. 27449594 10.1016/j.drup.2016.04.003

[51] Murakami S, Nakashima R, Yamashita E, Yamaguchi A. Crystal structure of bacterial multidrug efflux transporter AcrB. Nature. 2002;419(6907):587–593. 12374972 10.1038/nature01050

[52] Onishi M, Mizusawa M, Tsuchiya T, Kuroda T, Ogawa W. Suppression of stop codon UGA in acrB can contribute to antibiotic resistance in Klebsiella pneumoniae ATCC10031. Gene. 2014;534(2):313–319. 24498649

[53] Luo Z, Qiu H, Peng X, Tan Q, Chen B, Gu Q, Liu H, Zhou H. Development of potent inhibitors targeting bacterial prolyl-tRNA synthetase through fluorine scanning-directed activity tuning. Eur J Med Chem. 2025;291:117647. 40253792 10.1016/j.ejmech.2025.117647

[54] Terreni M, Taccani M, Pregnolato M. New antibiotics for multidrug-resistant bacterial strains: Latest research developments and future perspectives. Molecules. 2021;26(9):2671. 34063264 10.3390/molecules26092671 PMC8125338

[55] Lozano-Huntelman NA, Bullivant A, Chacon-Barahona J, Valencia A, Ida N, Zhou A, Kalhori P, Bello G, Xue C, Boyd S, et al. The evolution of resistance to synergistic multi-drug combinations is more complex than evolving resistance to each individual drug component. Evol Appl. 2023;16(12):1901–1920. 38143903 10.1111/eva.13608 PMC10739078

[56] Morales-Durán N, León-Buitimea A, Morones-Ramírez JR. Unraveling resistance mechanisms in combination therapy: a comprehensive review of recent advances and future directions. Heliyon. 2024;10(6):e27984. 38510041 10.1016/j.heliyon.2024.e27984 PMC10950705

[57] Tängdén T. Combination antibiotic therapy for multidrug-resistant Gram-negative bacteria. Ups J Med Sci. 2014;119(2):149–153. 24666223 10.3109/03009734.2014.899279 PMC4034552

[58] Liu S, Liu J, Fu N, Kornmatitsuk B, Yan Z, Luo J. Phenylalanine-arginine β-naphthylamide could enhance neomycin-sensitivity on *Riemerella anatipestifer* in vitro and in vivo. Front Microbiol. 2023;13:985789. 36713163 10.3389/fmicb.2022.985789 PMC9873997

