# Supplementary material for: Inhibition of prolyl-tRNA synthetase and efflux pumps as a dual-targeting strategy against multidrug-resistant bacteria
Source: J Enzyme Inhib Med Chem. 2026 Mar 18;41(1):2640718. doi: 10.1080/14756366.2026.2640718 (PMC13003883; doi:10.1080/14756366.2026.2640718)
Supplement: Supporting_Information_anonymous-cl.docx [file IENZ_A_2640718_SM3118.docx]

**Supporting Information**

**Inhibition of Prolyl-tRNA Synthetase and Efflux Pumps as a Dual-Targeting Strategy Against Multidrug-Resistant Bacteria**

1. **Certificate of Analysis Data for Compounds Acquired via Custom Synthesis Service (BioDuro-Sundia)**
   1. **Cpd-6 (7-bromo-6-fluoro-3-{2-oxo-3-[(2S)-piperidin-2-yl]propyl}quinazolin-4(3H)-one)**


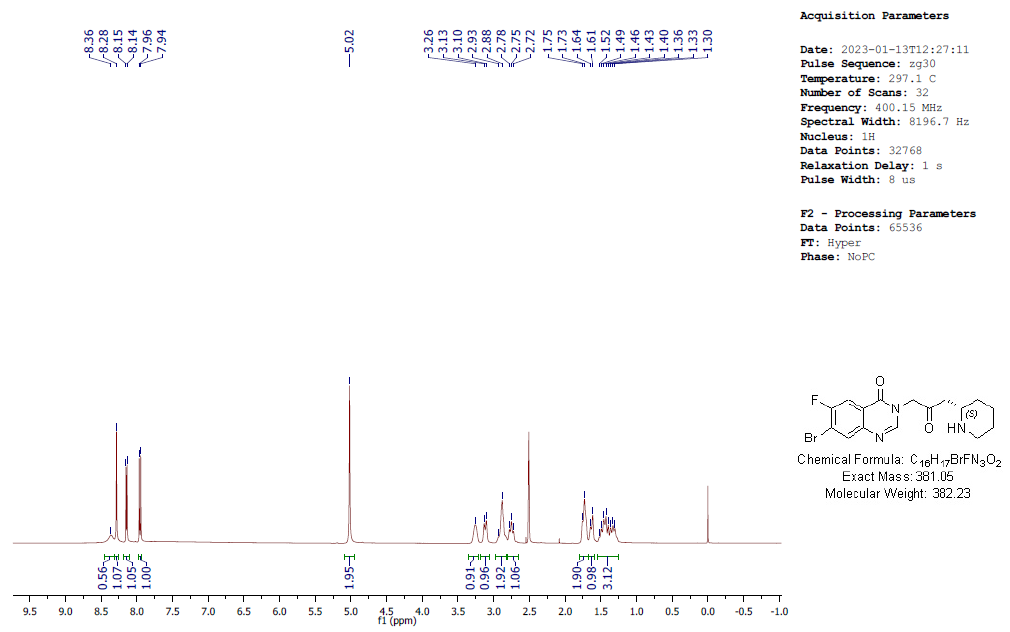


Chemical structure and ^1^H NMR spectrum of **Cpd-6** (400.15 MHz, DMSO-d6).


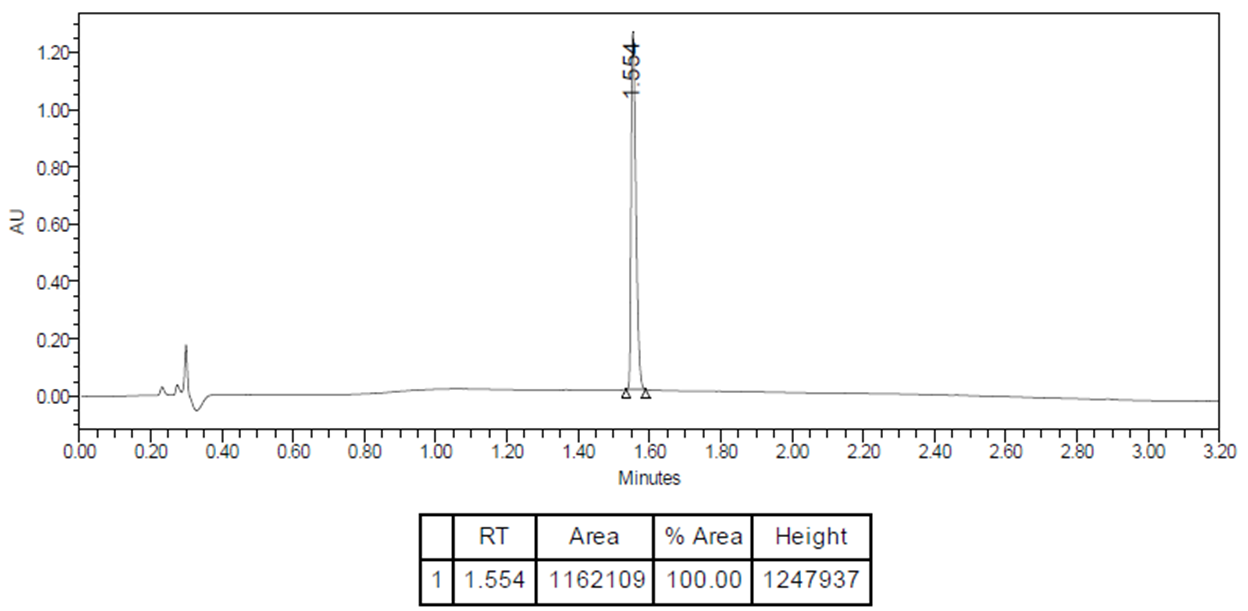


UPLC chromatogram of **Cpd-6** (Waters Acquity UPLC; Acquity BEH C18 column, 2.1×50 mm, 1.7 μm; mobile phases: A = 0.05% TFA in water, B = 0.05% FA in acetonitrile; gradient: 5–95% B over 2.0 min; detection at 214 nm; RT = 1.554 min; purity = 100%).


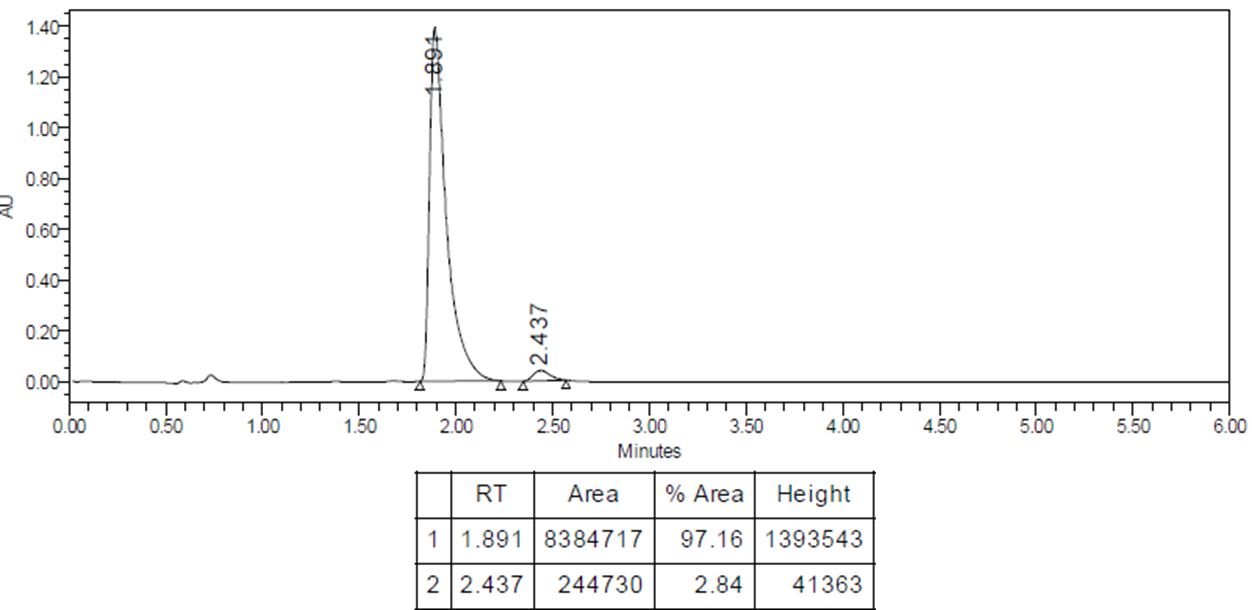


UPCC chromatogram of **Cpd-6** (Waters Acquity UPCC; Daicel CHIRALPAK IA, 3×150 mm, 3 μm; mobile phase: CO₂/MeOH (0.1% DEA) 50:50; flow rate: 1.5 mL/min; column temperature: 37 °C; detection at 214 nm; RT = 1.89 min; purity = 97.2%).


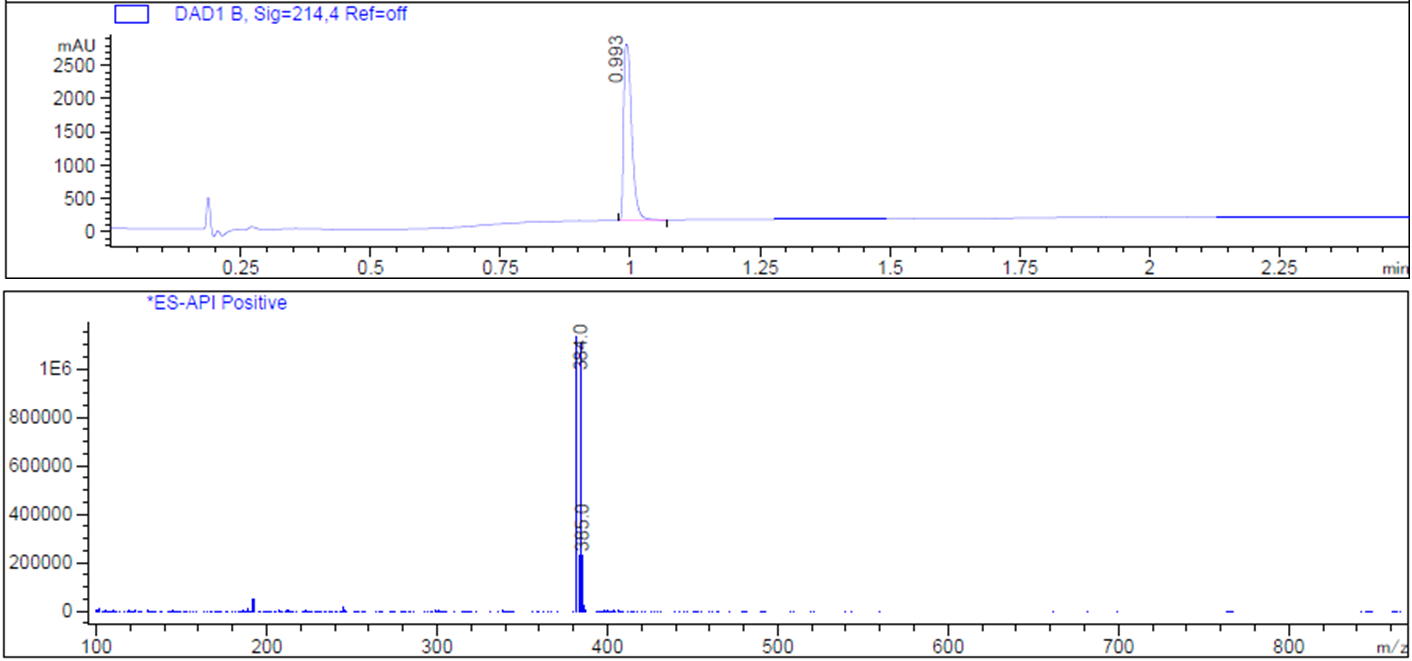


LC–MS analysis of **Cpd-6**. UV chromatogram at 214 nm (top panel) and ESI-MS spectrum (bottom panel) aquired at RT = 1.02 min showing [M+H]+ at m/z 385.0 (calcd 385.1).

- 1. **Cpd-7 (7-bromo-3-{3-[(3S)-1,2-diazinan-3-yl]-2-oxopropyl}-6-fluoroquinazolin-4(3H)-one)**


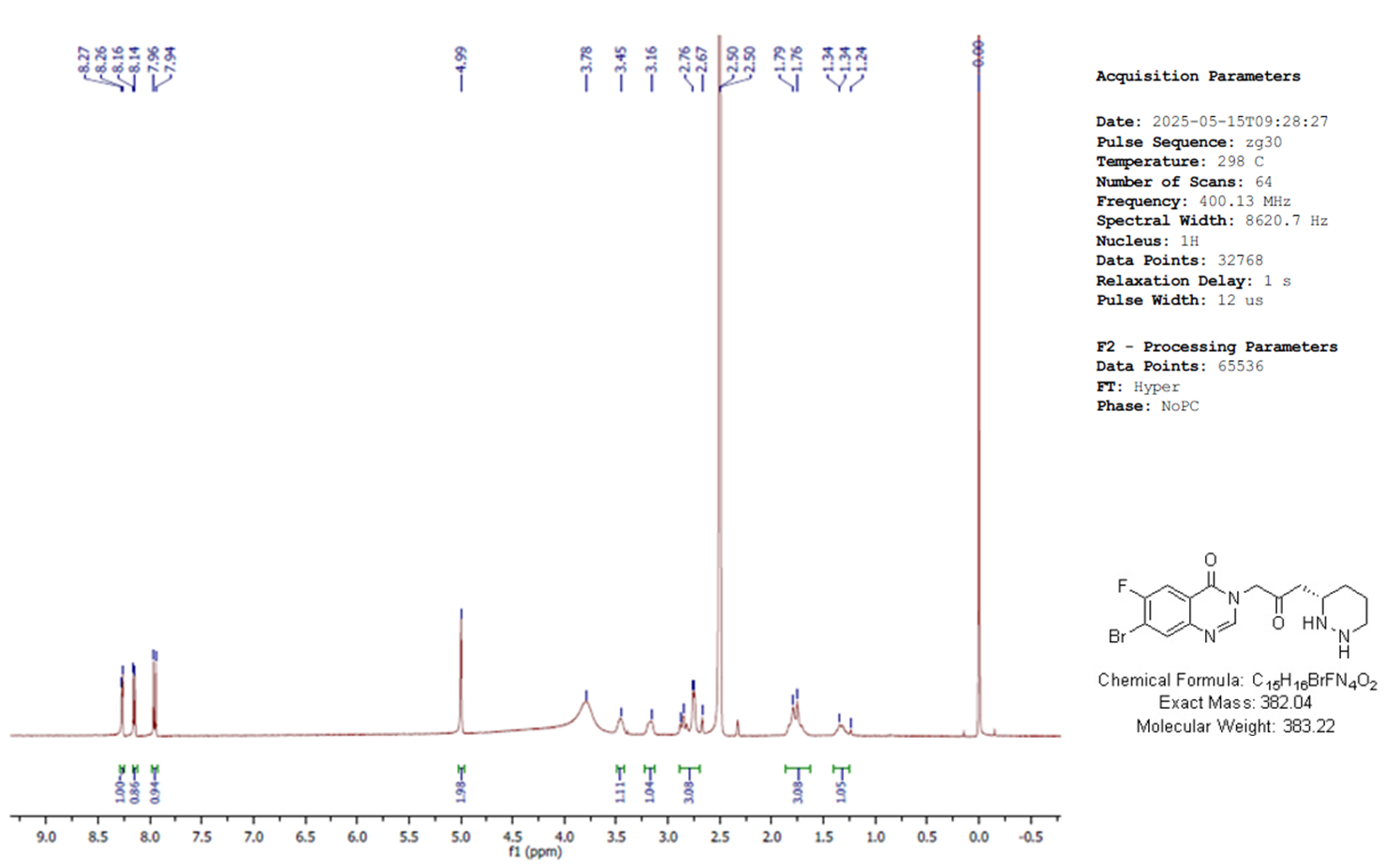

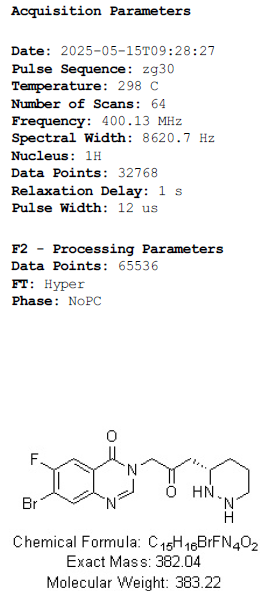


Chemical structure and ^1^H NMR spectrum of **Cpd-7** (400.13 MHz, DMSO-d6).


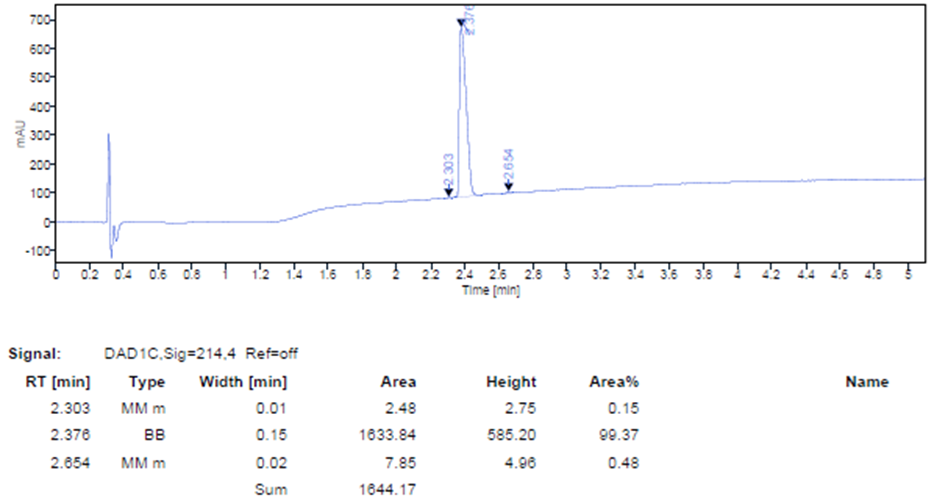


HPLC chromatogram of **Cpd-7** (Agilent 1260 II, Poroshell 120 EC-C18, 3.0×50 mm, 2.7 μm; mobile phases: A = 0.1% FA in water, B = 0.1% FA in acetonitrile; gradient: 5–100% B in 4 min; detection at 214 nm; RT = 2.376 min; purity = 99.4%).


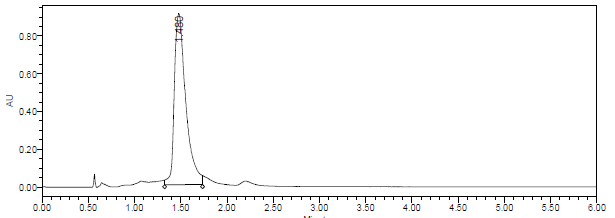


UPCC chromatogram of **Cpd-7** (Waters Acquity UPCC; Daicel CHIRALPAK IB, 4.6×250 mm, 5 μm; mobile phase: CO₂/MeOH (0.1% DEA) 50:50; flow rate: 1.5 mL/min; column temperature: 37 °C; detection at 214 nm; RT = 1.48 min; purity = 100%).


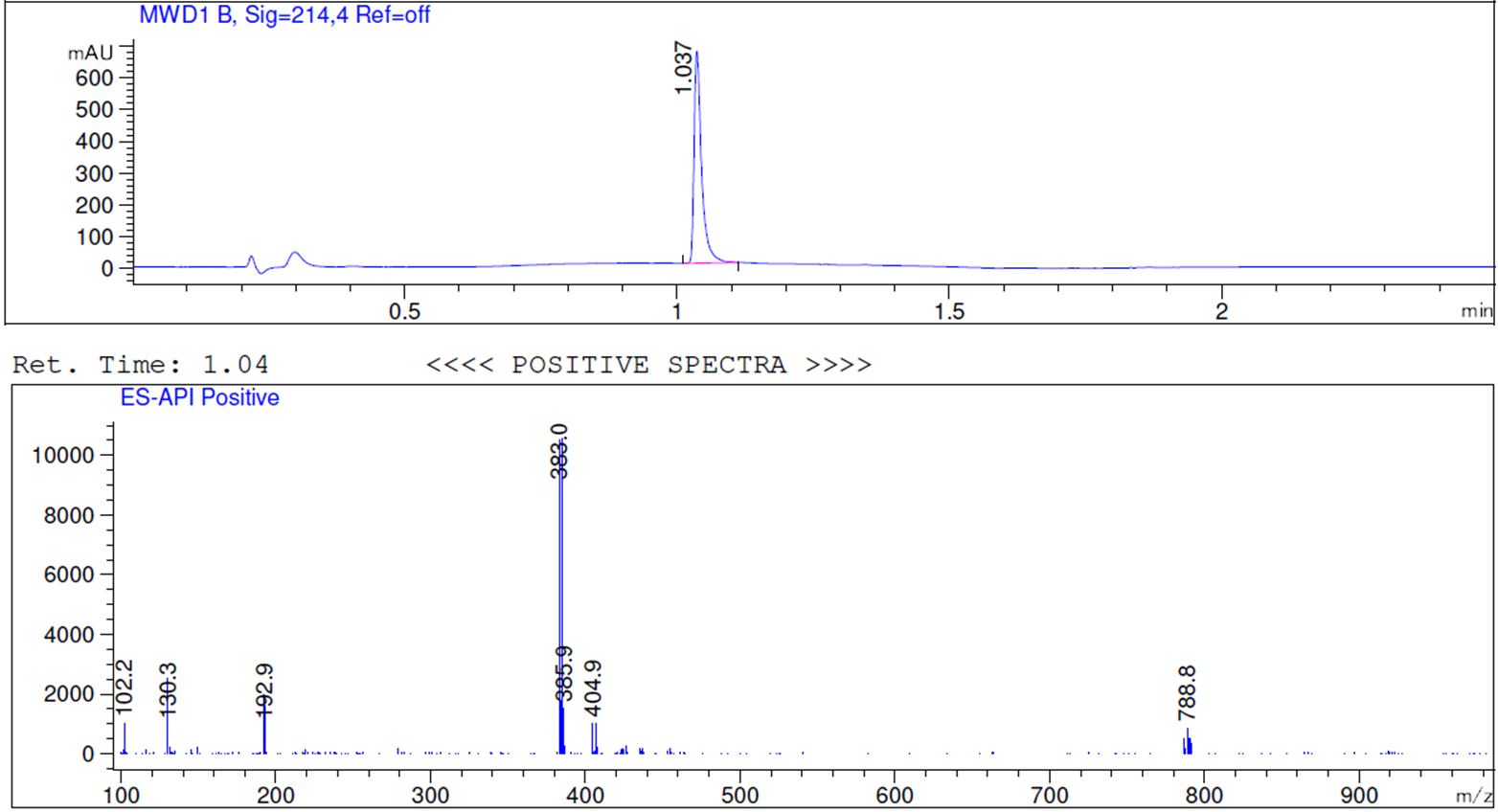


LC–MS analysis of **Cpd-7**. UV chromatogram (top panel) at 214 nm and ESI-MS spectrum (bottom panel) at RT = 1.04 min, showing [M+H]+ at m/z 385.0 (calcd 385.1).

- 1. **BSP-1 (1-(4,5-dichloro-1H-1,3-benzimidazol-2-yl)-3-[(2S)-piperidin-2-yl]propano-2-one)**


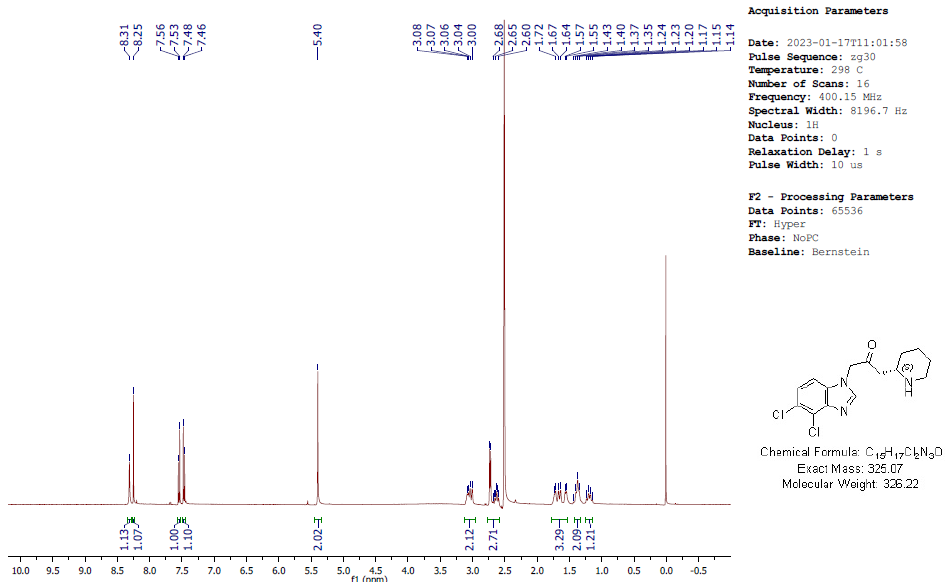


Chemical structure and ^1^H NMR spectrum of **BSP-1** (400.15 MHz, DMSO-d6).


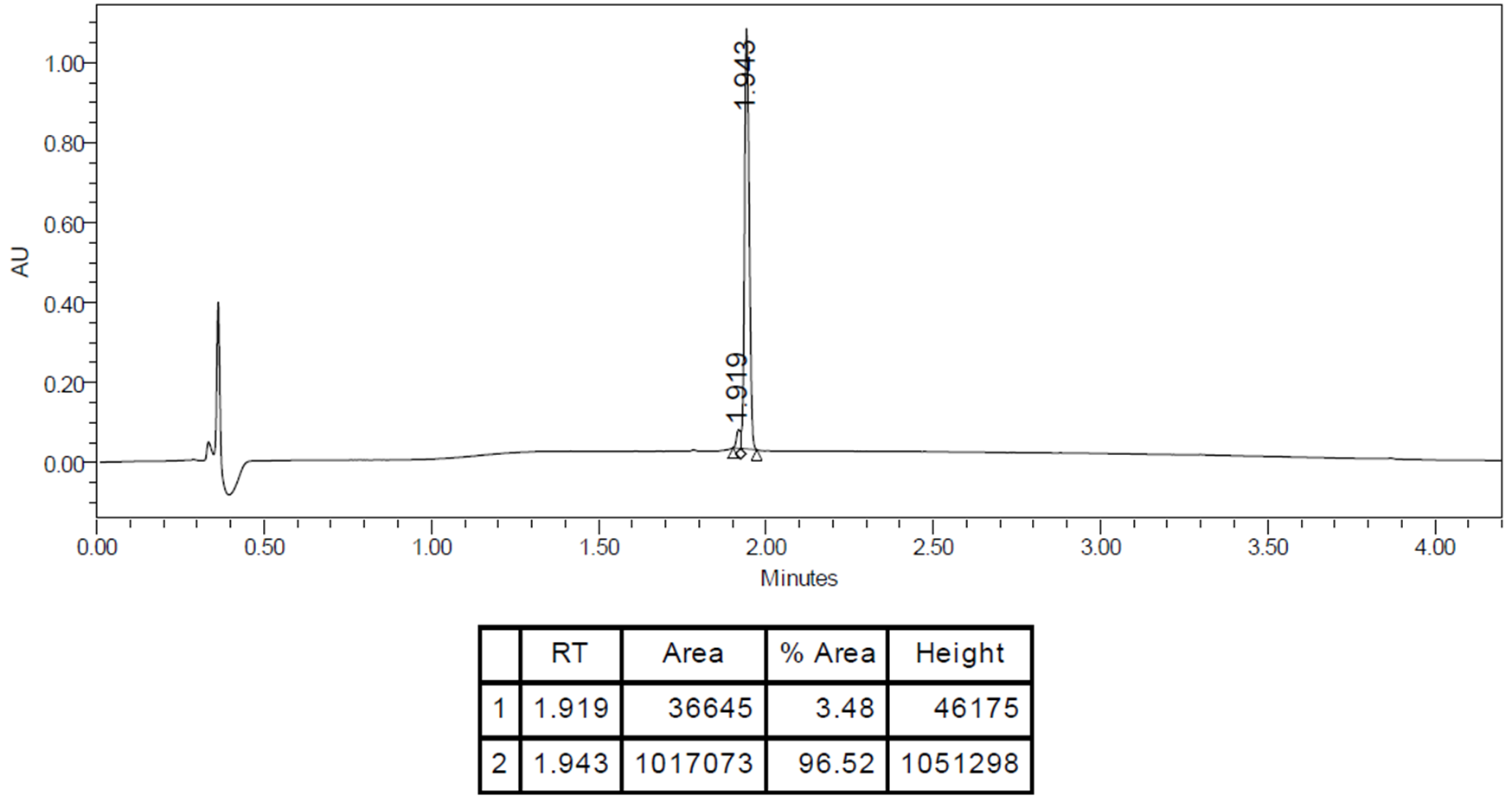


HPLC chromatogram of **BSP-1** (Waters Acquity UPLC, Acquity BEH C18 column, 2.1×50 mm, 1.7 μm; mobile phases: A = water (0.05% TFA), B = acetonitrile (0.05% TFA); gradient: 5-95% B over 2.8 min; flow rate = 0.4 mL/min; detection at 214 nm; RT = 1.943 min; purity = 96.5%).


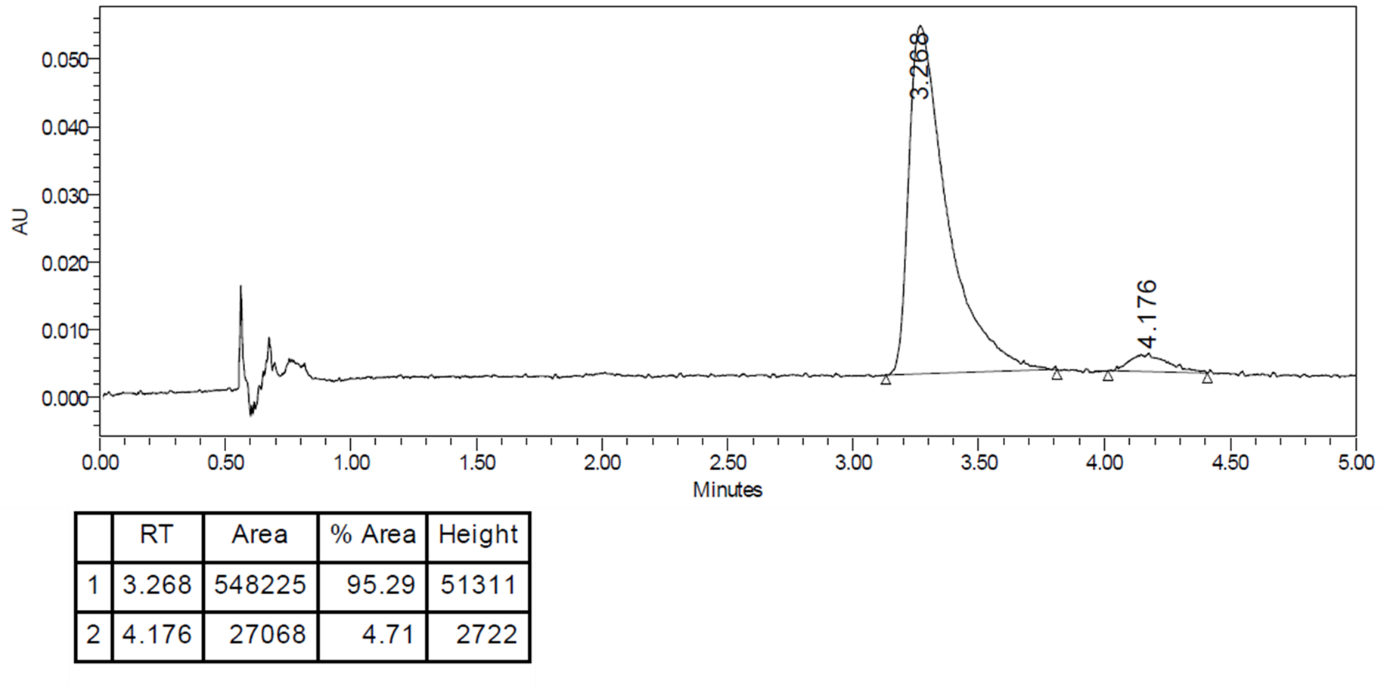


UPCC chromatogram of **BSP-1** (Waters Acquity UPCC; Daicel CHIRALPAK IE, 3.0×150 mm, 3 μm; mobile phase: CO₂/EtOH (0.1% DEA) 60:40; flow rate: 1.5 mL/min; column temperature: 37 °C; detection at 214 nm; RT = 3.268 min; purity = 95.3%).


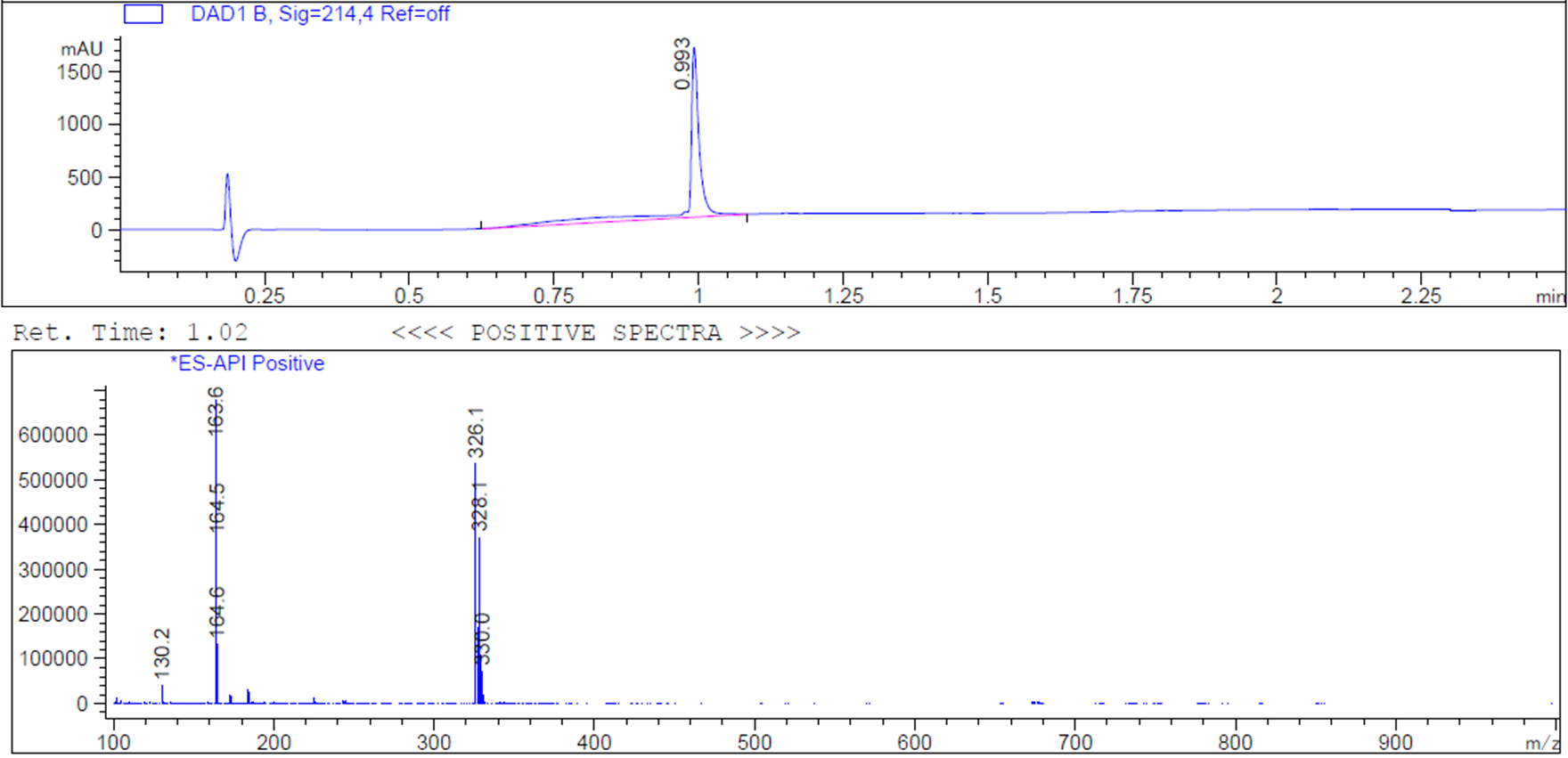


LC–MS analysis of **BSP-1**. UV chromatogram at 214 nm (top panel) and ESI-MS spectrum (bottom panel) at RT = 1.02 min, showing [M+H]+ at m/z 326.1 as dominant peak, along with minor fragment ions.

- 1. **Deoxy-halofuginone (d-HF)**


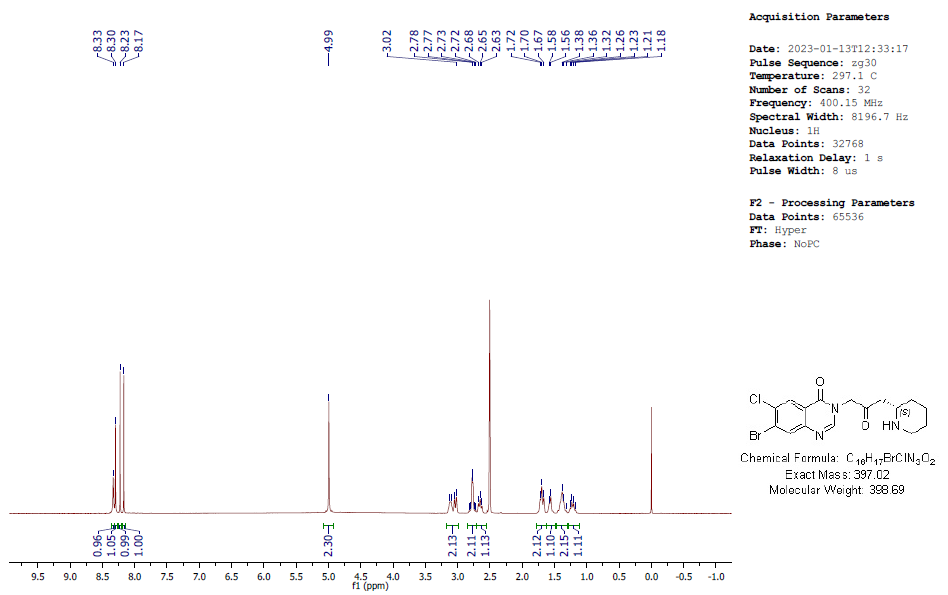


Chemical structure and ^1^H NMR spectrum of **d-HF** (400.15 MHz, DMSO-d6).


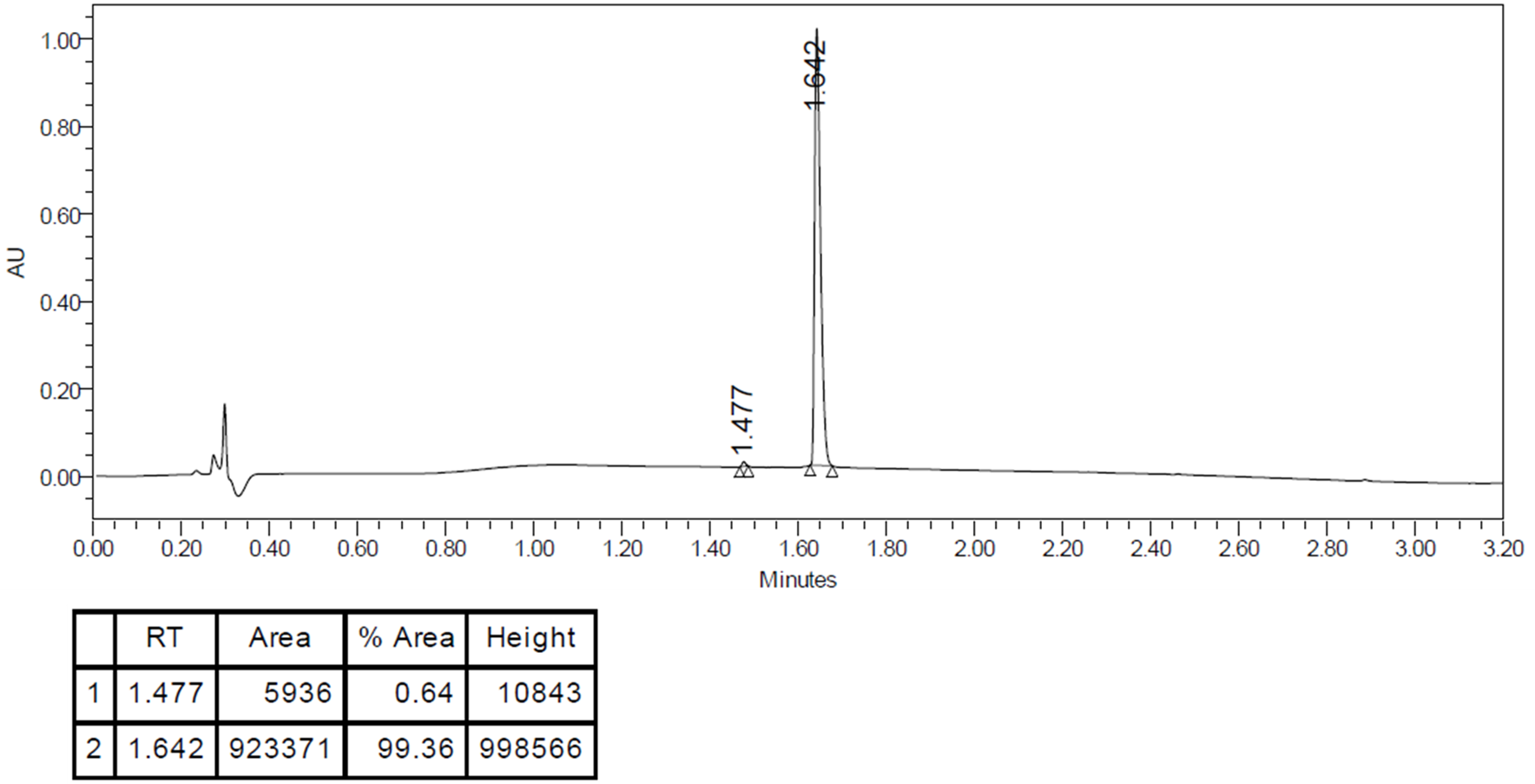


HPLC chromatogram of **d-HF** (Waters Acquity UPLC, Acquity BEH C18 column, 2.1×50 mm, 1.7 μm; mobile phases: A = water (0.05% TFA), B = acetonitrile (0.05% TFA); gradient: 5-95% B over 2.0 min; flow rate = 0.5 mL/min; detection at 214 nm; RT = 1.642 min; purity = 99.4%).


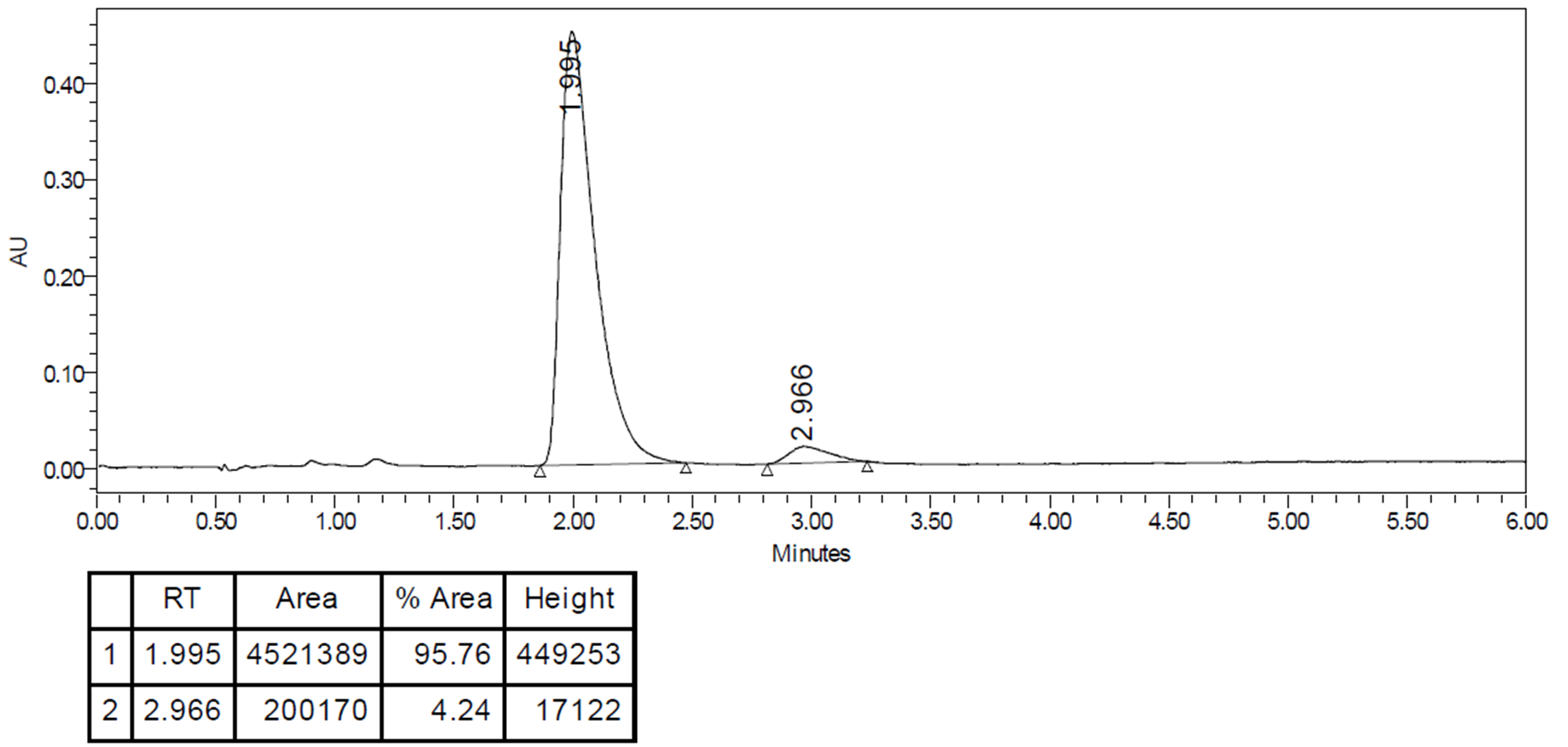


UPCC chromatogram of **d-HF** (Waters Acquity UPCC; Daicel CHIRALPAK IA, 4.6×250 mm, 5 μm; mobile phase: CO₂/MeOH (0.1% DEA) 50:50; flow rate: 1.5 mL/min; column temperature: 37 °C; detection at 214 nm; RT = 1.995 min; purity = 95.8%).


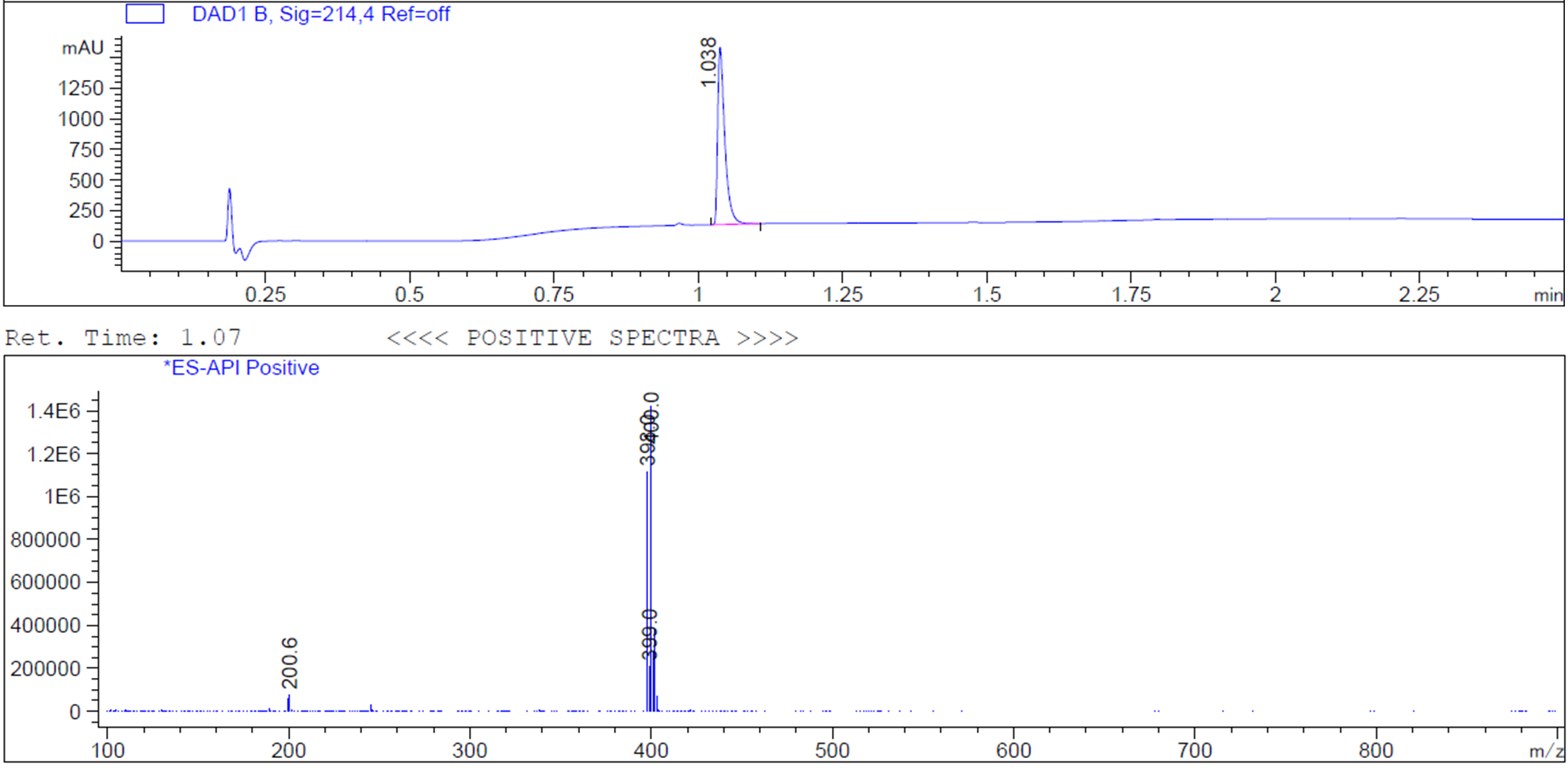


LC–MS analysis of **d-HF**. UV chromatogram at 214 nm (top panel) and ESI-MS spectrum (bottom panel) at RT = 1.07 min, showing [M+H]+ at m/z 400.0 as the dominant peak, along with minor fragment ions.

1. **Chemical synthesis of d-FF and Cpd-5**

Reagents were obtained from suppliers and used without prior purification, unless otherwise specified. The reactions involving moisture-sensitive reagents were performed under a nitrogen stream in oven-dried glassware. Dichloromethane (DCM) and diisopropylethylamine (DIPEA) were distilled over calcium hydride, whereas tetrahydrofuran (THF) was distilled over calcium hydride and then over sodium benzophenone before use. Methanol was treated with magnesium metal, refluxed, distilled, and kept over 4A molecular sieves. Acetone underwent treatment with Drierite®, was distilled, and then stored over 4A molecular sieves.

Chemical reactions were monitored by thin layer adsorption chromatography (TLC) on aluminum plates with 0.25 mm Merck 60® silica gel. Spots were detected under 254 nm UV light irradiation and/or by immersion in solutions of *p*-anilsaldehyde, phosphomolybdic acid, vanillin, Erlich's reagent or Dragendof's reagent. Compounds were purified by flash column chromatography using Aldrich silica gel 60 (70-230 mesh). The diameter and length of the columns, in addition to the silica height used, were calculated based on the mass of the samples and the characteristics of the samples according to the methodology described by Still et al (1978).

Fourier-Transformed Infrared (FTIR) spectra were obtained with a Nicolet Impact 400 spectrophotometer using KBr pellets or NaCl windows. Hydrogen nuclear magnetic resonance (^1^H-NMR) and carbon-13 nuclear magnetic resonance (^13^C-NMR) spectra were obtained in a Bruker WM-250 or Varian Inova 500. Chemical shifts were expressed in parts per million (ppm) and referred to tetramethylsilane or deuterated chloroform signals. When not specified, the solvent used was deuterated chloroform. The multiplicity of proton signals in ^1^H-NMR spectra was indicated according to the convention: s (singlet), sl (broad singlet), d (doublet), dd (double doublet), dt (double triplet), dqt (double quintet), t (triplet), q (quartet), qt (quintet) and m (multiplet). The coupling constants are expressed in Hertz (Hz).

- 1. **Benzyl 2-oxopyrrolidine-1-carboxylate (1)**

To a round-bottom flask containing a solution of 2-pyrrolidone (4.26 g, 50 mmol) in THF (85 mL) under a nitrogen flow at -78 °C, 1.6 M *n*-BuLi (32 mL, 50 mmol) was added dropwise. After stirring for 30 minutes at -78 °C, benzyl chloroformate (9.1 mL, 64 mmol) was added dropwise. Upon completion of the addition, the mixture was stirred at -78 °C for an additional hour. The mixture was cooled to room temperature, diluted with 34 mL water, and extracted three times with 70 mL of ethyl acetate. Combined organic layers were dried over MgSO_4_ and concentrated by rotary evaporation. The product was purified by flash column chromatography (Hex/AcOEt 2:1).

A**ppearance:** colorless oil

**Yield:** 92%

**FTIR** **(NaCl, film):** 1754, 1715, 1706 cm^-1^

**^1^H NMR** **δ:** 2.03 (q, 2H, *J* = 7.5 Hz), 2.54 (t, 2H, *J* = 7.5 Hz), 3.81 (t, 2H, *J* = 7.5 Hz), 5.28 (s, 2H), 7.28-7.49 (m, 5H)

**^13^C NMR δ:** 17.5; 32.8; 46.4; 68.0; 128.2; 128.4; 128.6; 135.3; 151.7; 174.0

- 1. **Benzyl 2-oxopiperidine-1-carboxylate (2)**

Prepared as described above except by using piperidin-2-one as the starting material.

**Appearance:** colorless oil

**Yield:** 89%

**FTIR (NaCl, film):** 1774, 1713 cm^-1^.

**^1^H NMR δ:** 1.75-1.82 (m, 4H); 2.48-2.53 (m, 2H); 3.69-3.78 (m, 2H); 5.26 (s, 2H); 7.27-7.50 (m, 5H).

**^13^C NMR δ:** 20.4; 22.7; 34.9; 46.6; 68.4; 128.1; 128.3; 128.6; 135.4; 154.1; 171.2.

- 1. **Benzyl 2-hydroxypyrrolidine-1-carboxylate (3)**^^[[1]](#footnote-1)^^

A solution of **1** (4.38 g, 20 mmol) in THF (100 mL) was cooled to -78°C, then diisobutylaluminum hydride (DIBAL-H) (1.2 M in toluene, 23.3 mL) was added dropwise. After stirring for 2 h at -78 °C, saturated potassium acetate (40 mL) was added, and the mixture warmed to room temperature. The reaction mixture was transferred to saturated NH_4_Cl/ethyl ether (1:3, v/v; 400 mL), stirred until a gelatinous precipitate formed, and filtered through Celite. The layers were separated, and the aqueous phase was extracted three times with ethyl ether. Combined organic phases were washed twice with saturated NH_4_Cl, dried over K_2_CO_3_, concentrated, and purified by flash chromatography with 1% triethylamine (eluent: Hex:AcOEt 4:1, v/v).

**Appearance:** colorless oil

**Yield:** 93%.

**FTIR (NaCl, film):** 3448, 1786, 1751, 1705, 1300 cm^-1^.

**^1^H** **NMR δ:** [mixture of rotamers] 1.80-2.20 (m, 4H); 2.95 (sl, 0.3H); 3.27-3.44 (m, 1H); 3.52-3.68 (m, 1H); 3.78 (sl, 0.7H); 5.17 (d, 2H, *J* = 8.2 Hz); 5.44-5.59 (m, 1H); 7.28-7.44 (m, 5H).

**^13^C NMR δ:** [mixture of rotamers] 22.0; 22.8; 32.7; 33.6; 45.8; 46.2; 66.9; 67.1; 81.3; 87.1; 127.9; 128.1; 128.3; 128.5; 128.6; 136.5; 155.4.

- 1. **Benzyl 2-hydroxy-piperidine-1-carboxylate (4)**

Prepared as for compound **3**, but using compound **2** as the starting material.

**Appearance:** colorless oil

**Yield:** 90%

**FTIR (NaCl, film):** 3438, 1758, 1751, 1702, 1313 cm^-1^.

**^1^H** **NMR δ:** [mixture of rotamers] 1.44-1.90 (m, 7H); 3.19 (td, 1H); 3.89 (d, 1H); 5.15 (s, 2H); 5.77 (m, 1H); 7.27-7.50 (m, 5H).

**^13^C NMR δ:** [mixture of rotamers] 17.6; 24.7; 30.5; 39.4; 67.2; 75.0; 125.6; 127.9; 128.1; 136.4; 160.2.

- 1. **Benzyl 2-methoxypyrrolidine-1-carboxylate (5)**^^[[2]](#footnote-2)^^

To a solution of **3** (3.98 g, 18.0 mmol) and anhydrous MeOH (18 mL, 444 mmol) in DCM (30 mL), Sc(OTf)_3_ (0.886 g, 0.182 mmol) was added and allowed to stir for 3 h. A saturated NaHCO_3_ solution was subsequently added and the mixture was extracted twice with DCM. The combined organic extracts were washed with brine, dried over Na_2_SO_4_, and concentrated in vacuo. The obtained product was used in the subsequent step without prior purification.

**Appearance:** colorless oil

**Yields:** 100%

**FTIR (NaCl, film):** 2892, 1707, 1358, 1185, 1084 cm^-1^.

**^1^H NMR** **δ: [**mixture of rotamers] 1.68-2.17 (m, 4H); 3.27 (sl, 1H); 3.40 (sl, 3H); 3.47-3.59 (m, 1H); 5.11-5.26 (m, 3H); 7.28-7.40 (m, 5H).

**^13^C NMR δ:** [mixture of rotamers] 21.7; 22.6; 32.0; 32.6; 45.8; 45.9; 55.4; 56.0; 66.9; 67.1; 88.5; 89.1; 127.8; 128.0; 128.5; 136.6; 154.4.

- 1. **Benzyl 2-methoxypiperidine-1-carboxylate (6)**

Prepared as compound **5**, but using compound **4** as the starting material.

**Appearance:** colorless oil

**Yield:** 100%.

**FTIR (NaCl, film):** 2868, 1705, 1169, 1086, 1040 cm^-1^.

**^1^H** **NMR δ:** [mixture of rotamers] 1,.25-2.00 (m, 6H); 2.94 (q, 1H, *J* = 14 Hz); 3.19 (s, 1.5H); 3.25 (s, 1.5H); 3.98 (t, 1H, *J* = 14 Hz); 5.15 (s, 2H); 5,34 (s, 0.5H); 5,43 (s, 0.5H); 7.27-7.60 (m, 5H).

**^13^C NMR δ:** [mixture of rotamers] 18.4; 24.9; 25.1; 30.0; 30.3; 38.7; 39.4; 54.3; 55.6; 67.0; 67.2; 82.0; 117.8; 125.6; 128.0; 128.5; 136.4; 154.2.

- 1. **Benzyl 2-pyrroline-1-carboxylate (7)**

In a round-bottom flask containing a solution of **3** (2.89 g, 12.3 mmol) in 12 mL of DCM, under stirring, at 0°C, DIPEA (5.10 mL, 30.8 mmol) was added and then, in a single portion, trimethylsilyl trifluoromethanesulfonate (TMSOTf) (2.50 mL, 13.5 mmol) was added. After 5 min, the solvent was removed by rotary evaporation and the resulting residue was subjected to a flash chromatographic column doped with 1% triethylamine.

**Appearance:** colorless oil

**Yield:** 89%

**FTIR (NaCl, film):** 1706, 1619, 1448, 1422, 1348, 1128 cm^-1^.

**^1^H** **NMR δ:** [mixture of rotamers] 2.56-2.75 (m, 2H); 3.70-3.86 (m, 2H); 5.04 (sl, 0.6H); 5.09 (sl, 0.4H); 5.17 (s, 2H); 6.55 (sl, 0.6H); 6.63 (sl, 0.4H); 7.28-7.43 (m, 5H).

**^13^C NMR δ:** [mixture of rotamers] 28.6; 29.7; 45.1; 45.2; 66.9; 67.1; 108.6; 108.8; 127.9; 128.0; 128.1; 128.5; 129.0; 129.7; 136.6; 152.1; 1528.

- 1. **Benzyl *trans*-(±)-3-bromo-2-methoxypyrrolidine-1-carboxylate (8)**

Carbamate **7** (1.02 g, 5.0 mmol) and 10 mL of anhydrous methanol were added to a flask and the system was cooled to 0 °C. To this mixture was then added sodium hydroxide (0.24 g, 6.0 mmol), followed by titration with a 2.0 M solution of bromine in methanol. The solvent was removed under vacuum and the resulting residue was added with saturated sodium bicarbonate solution and extracted three times with ethyl acetate. The organic phases were combined, dried with magnesium sulfate, and concentrated by rotary evaporator. Purification was carried out by flash chromatography (Eluent:AcOEt/Hex 1:4,v/v).

**Appearance:** colorless oil

**Yield:** 88%

**FTIR (NaCl, film):** 1711, 1175, 1113, 1077, 697, 630 cm^-1^.

**^1^H** **NMR δ:** [mixture of rotamers] 2.19 (dd,1H, *J* = 12.0 Hz e 7.5 Hz); 2.62 (m, 1H); 3.31 (s, 1.2H); 3.44 (s, 1.8H); 3.57 (t, 1H, *J* = 7.5 Hz); 3.65-3.77 (m, 1H); 4.24 (d, 1H, ^3^*J* = 5.0 Hz); 5.19 (sl, 2H); 5.20-5.29 (m, 0.6H); 5.34 (sl, 0.4 H); 7.27-7.41 (m, 5H).

**^13^C NMR δ:** [mixture of rotamers] 31.5; 32.4; 44.0; 44.2; 49.8; 50.5; 56.0; 56.5; 67.3; 67.4; 94.5; 95.1; 127.8; 127.9; 128.1; 128.5; 136.2; 136.3; 155.0; 155.8.

- 1. **Benzyl *trans*-(±)-3-bromo-2-(2-oxopropyl) pyrrolidine-1-carboxylate (9)**

To a solution of **8** (1.10 g, 3.50 mmol) dissolved in DCM at -78 °C under magnetic stirring, a solution of 1 M titanium tetrachloride in dichloromethane (5.3 mL, 5.3 mmol), previously cooled to -78 °C, was slowly added dropwise. Upon completion of the TiCl_4_ addition, isopropenyl acetate (0.77 mL, 7.0 mmol) was added slowly during 5 min. The reaction mixture was kept at -78 °C for 2 h. Then, it was brought to room temperature, diluted with DCM, and poured into a saturated sodium bicarbonate solution previously cooled to 0°C. The organic and aqueous phase was separated in a separatory funnel, and the aqueous phase was extracted three times with ethyl acetate. The organic phases were combined and washed with brine, dried over MgSO_4_, and the solvent removed in a rotary evaporator. Purification was performed on a flash chromatographic column (Eluent: AcOEt/Hex 3:7,v/v).

**Appearance:** White solid, M.P. 85.5-87.5 ^o^C.

**Yield:** 75%

**FTIR (NaCl, tablet):** 1701, 698, 606 cm^-1^.

**^1^H** **NMR δ:** [mixture of rotamers] 2.03 (sl, 1.2H); 2.18 (m, 1.8H); 2.21-2.27 (m, 1H); 2.82 (d, 0.4H, ^1^*J* = 17.5 Hz); 3.07 (dd, 0.6H, ^1^*J* = 17.5 Hz e ^3^*J* = 2.5 Hz); 3.48-3.62 (m, 1H); 3.63-3.85 (m, 1H); 4.36 (sl, 1H); 4.48 (d, 1H, *J* = 8.0 Hz); 5.14 (sl, 2H); 7.27-7.43 (m, 5H).

**^13^C NMR δ:** [mixture of rotamers] 25.6; 25.7; 30.2; 32.8; 33.7; 44.4; 44.7; 46.5; 47.6; 50.4; 51.2; 63.5; 64.1; 67.0; 67.2; 67.9; 127.8; 127.9; 128.0; 128.5; 136.5; 154.5; 206.0.

**HRMS (ESI /H^+^; m/z)**: calcd. for C_15_H_19_BrNO_3_^+^: 340.0548; found: 340.0595

- 1. **Benzyl 2-(2-oxopropyl)piperidine-1-carboxylate (10)**

Prepared as compound **9** except that compound **6** was used as the starting material.

**Appearance:** colorless viscous oil

**Yield:** 92%.

**FTIR (NaCl, film):** 1694, 1651 cm^-1^.

**^1^H** **NMR δ:** 1.35-1.70 (m, 6H); 2.12 (sl, 3H); 2.67 (dl, 2H, ^3^*J* = 7.5 Hz); 2.84 (t, 1H, *J*= 13.5 Hz); 4.04 (dl, 1H, *J*= 12.0 Hz); 4.78 (m, 1H); 5.13 (sl, 2H); 7.33 (m, 5H).

**^13^C NMR δ:** 18.8; 25.2; 28.3; 30.0; 39.8; 44.3; 47.5; 67.1; 127.8; 127.9; 128.4; 136.7; 155.3; 206.8.

- 1. **Benzyl *trans*-(±)-3-bromo-2-(2-oxo-3-(4-oxoquinazolin-3(4H)-yl)propyl)pyrrolidine-1-carboxylate (11)**

To a solution of methyl ketone **9** (0.680 g, 2.00 mmol) in DCM at 0 °C, TMSOTf (0.48 mL, 2.7 mmol) was added under stirring. After 5 min, DIPEA (0.40 mL, 2.4 mmol) was added, and the temperature was maintained at 0 °C for 15 min. Then, *N-*bromosuccinimide (0.427 g, 2.40 mmol) was added, and the mixture was stirred for 1.5 h at room temperature. The mixture was poured into water and extracted twice with ethyl acetate. The combined organic phases were washed with saturated sodium bicarbonate solution, brine, dried over MgSO_4_, and the solvent was removed by rotary evaporation. The resulting residue was dissolved in 20 mL of dry dimethylformamide (DMF), and then quinazolin-4(3H)-one (0.351 g, 2.40 mmol) and potassium carbonate (0.331 g, 2.40 mmol) were added. The mixture was allowed to stir for 30 min at room temperature and then poured into 100 mL of water. The aqueous phase was extracted three times with 150 mL of ethyl acetate and the combined organic phases were washed with water and brine, dried over MgSO_4_ and the solvent was removed by rotary evaporator. Purification was carried out by flash column chromatography (Eluent: EtOAc/petroleum ether 2:1, v/v).

**Appearance:** amber viscous oil.

**Yield:** 38%

**FTIR (NaCl, film):** 1729, 1680, 1613, 736, 697 cm^-1^.

**^1^H** **NMR δ:** [mixture of rotamers] 2.10-2.31 (m, 1H); 2.31-2.54 (m, 1H); 2.64-3.07 (m, 2H); 3.53-3.66 (m, 1H); 3.66-3.84 (m, 1H); 4.30-4.42 (m, 1H); 4.49-4.58 (m, 1H); 4.74 (d, 1H, *J* = 17.5 Hz); 5.14 (sl, 2H); 7.30-7.41 (m, 5H); 7.45-7.57 (m, 1H); 7.63-7.84 (m, 2H); 7.99 (s, 1H); 8.27 (d, 1H, *J* = 7.8 Hz).

**^13^C NMR δ:** [mixture of rotamers] 25.6; 34.1; 44.3; 44.9; 50.3; 54.4; 64.4; 67.5; 122.1; 126.9; 127.6; 127.9; 128.0; 128.4; 128.5; 128.6; 128.8; 134.8; 136.6; 146.8; 148.5; 155.2; 161.3; 199.8.

**EMAR (ESI /H^+^; m/z)**: calcd. For C_23_H_23_BrN_3_O4^+^: 486.0855; found: 486.0757.

- 1. **Benzyl 2-(2-oxo-3-(4-oxoquinazolin-3(4H)-yl)propyl)piperidine-1-carboxylate (12)**

Prepared as compound **11** except that compound **10** was used as the starting material.

**Appearance:** White solid, M.P.: 127-129 ^o^C.

**Yield:** 61%.

**FTIR (KBr, tablet):** 1730, 1695 cm^-1^.

**^1^H** **NMR δ:** [mixture of rotamers] 1.38-1.76 (m, 6H); 2.69-3.04 (m, 3H); 3.92-4.15 (m, 1H); 4.74-5.27 (m, 5H); 7.27-8.38 (m, 10H).

**^13^C NMR δ:** [mixture of rotamers] 18.4; 18.8; 25.0; 39.8; 41.6; 54.1; 58.4; 67.4; 121.5, 125.9; 127.0; 127.8; 127.9; 128.0; 128.5; 135.0; 136.5; 145.5; 147.6; 160.2; 200.2.

- 1. **3-(2-oxo-3-(piperidin-2-yl)propyl)quinazolin-4(3H)-one (d-FF)**

A solution of **12** (0.150 g, 0.37 mmol) in 10 mL of 6 M aqueous HCl was heated under reflux for 1 h. The pH of the solution was brought to 9 by addition of portions of K_2_CO_3_ and then extracted with chloroform. The organic phase was washed with brine and dried over potassium carbonate and the solvent was removed by rotary evaporator. Purification was carried out by alumina column chromatography (Eluent: EtOAc/petroleum ether 2:1,v/v).

**Appearance:** White solid, M.P.: 138-140 ^o^C.

**Yield:** 69%

**FTIR** **(KBr, tablet):** 3307, 1730, 1667 cm^-1^.

**^1^H** **NMR δ:** 1.15-1.29 (m, 1H); 1.30-1.49 (m, 2H); 1.52-1.72 (m, 2H); 1.73-1.86 (m, 1H); 2.00 (s, 1H); 2.55-2.79 (m, 3H); 2.96-3.16 (m, 2H); 4.71 (d, 1H, ^1^*J* = 17.5 Hz); 4.84 (d, 1H, ^1^*J* = 17.5 Hz); 7.45-7.57 (m, 1H); 7.68-7.83 (m, 2H); 7.88 (s, 1H); 8.27 (d, 1H, *J* = 7.8 Hz).

**^13^C NMR δ:** 24.5; 26.0; 32.8; 46.8; 47.7; 52.8; 54.8; 21.8; 126.7; 127.4; 127.6; 134.5; 146.2; 148.2; 160.9; 202.1.

**HRMS (ESI /H^+^; m/z)**: calcd. for C_16_H_20_N_3_O_2_^+^: 286.1555; found: 286.0891

- 1. ***trans*-(±)-3-Bromo-2-(2-oxo-3-(4-oxoquinazolin-3(4H)-yl)propyl)pyrrolidinium dihydrochloride [Cpd-5.HCl]**

To a solution of compound **11** (0.150 g, 0.313 mmol) in 5 mL of methanol saturated with HCl was added Pd/C 10% (0.30 mg) and the mixture was subjected to 1 atm hydrogen atmosphere for 3 h. It was then filtered through Celite® and ethyl ether was added to the solution to allow the precipitation of *trans*-(+/-)-3-bromo-2-(2-oxo-3-(4-oxoquinazolin-3(4H)-yl)propyl)pyrrolidinium dihydrochloride (**Cpd-5.HCl**).

**Appearance:** White solid, M. P.: 191.0-193.0 ^o^C (decomp).

**Yield:** 81%

**FTIR (KBr, tablet**): 3431, 2919, 2916, 2812, 1740, 1703, 1664, 1389, 971, 769 cm^-1^.

**^1^H** **NMR δ:** 2.38-2.51 (m, 1H); 2.71 (td, 1H, *J* = 12.5 e 7.5 Hz); 3.32 (dd, 1H, *J* = 20.0 e 10.0 Hz); 3.49-3.87 (m, 2H); 4.32 (m, 1H); 4.48 (dd, 1H, *J* = 10.0 and 5.0 Hz); 5.11 (sl, 2H); 7.74 (t, 1H, *J* = 7.5 Hz); 7.81 (d, 1H, *J* = 7.5 Hz); 8.27 (d, 1H, *J* = 7.5Hz); 8.54 (s, 1H).

**^13^C NMR δ:** 33.6; 40.0; 44.1; 46.4; 55.1; 62.5; 120.1; 124.6; 126.5; 128.9; 136.2; 144.1; 148.3; 161.4; 201.6.

**HRMS (ESI /+; m/z)**: calcd. for C_15_H_17_BrN_3_O_2_^+^: 350.0504; found: 350.0495

- 1. **NMR Spectra of compounds of d-FF, Cpd-5, and reaction intermediate**


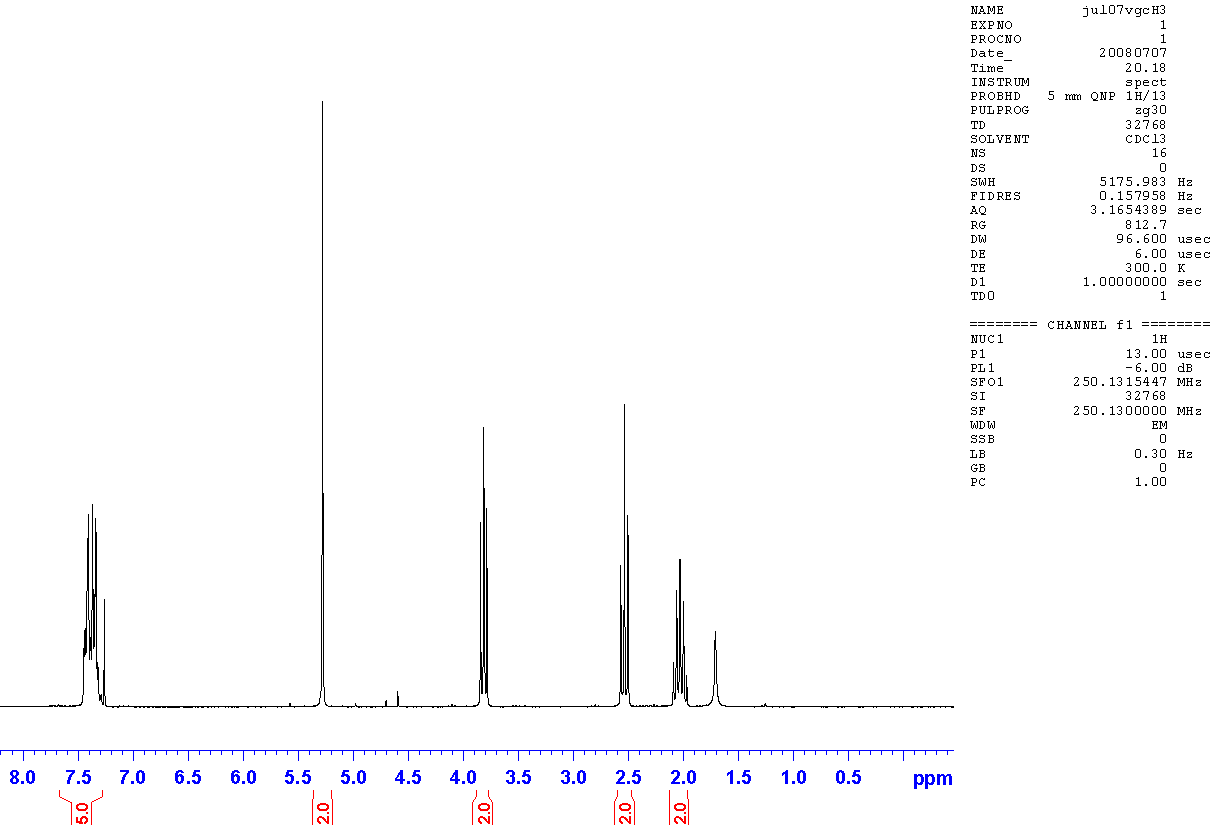


^1^H NMR (CDCl_3_, 250 MHz) of **1**.


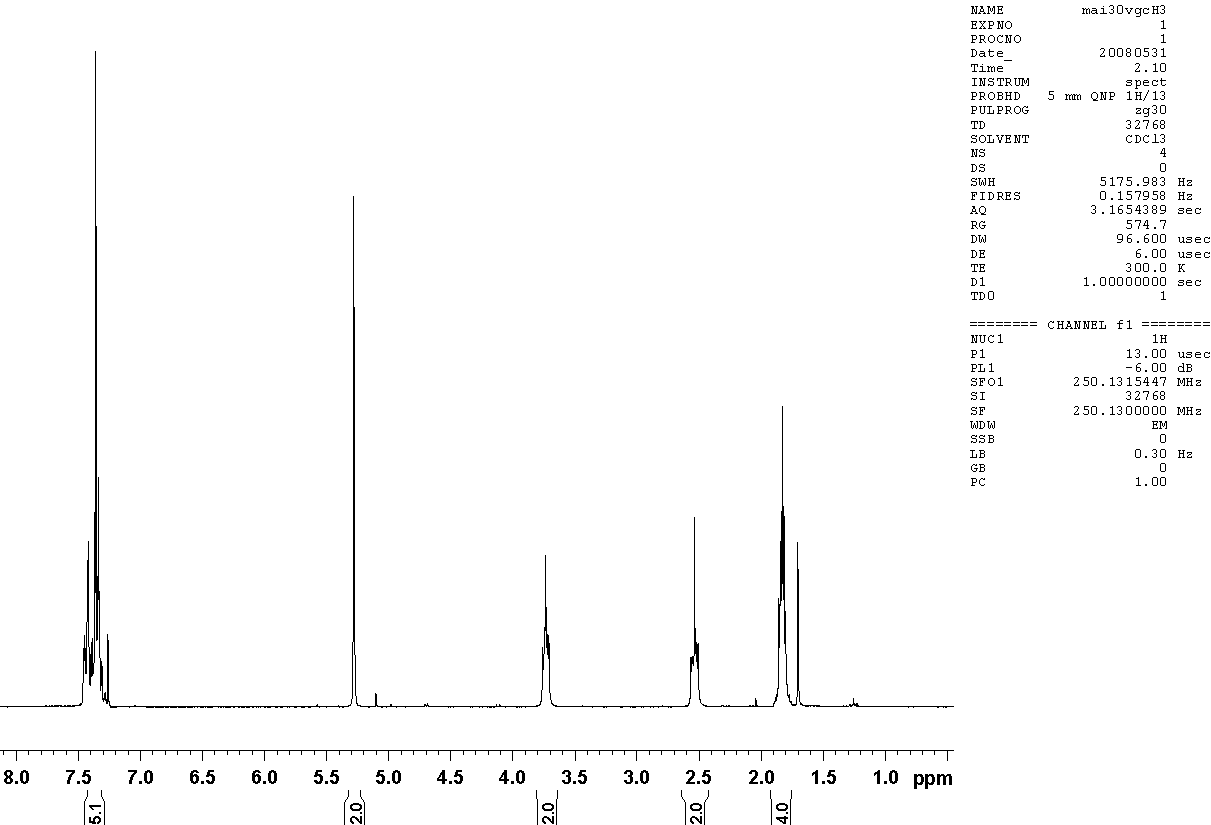


^1^H NMR (CDCl_3_, 250 MHz) of **2**.


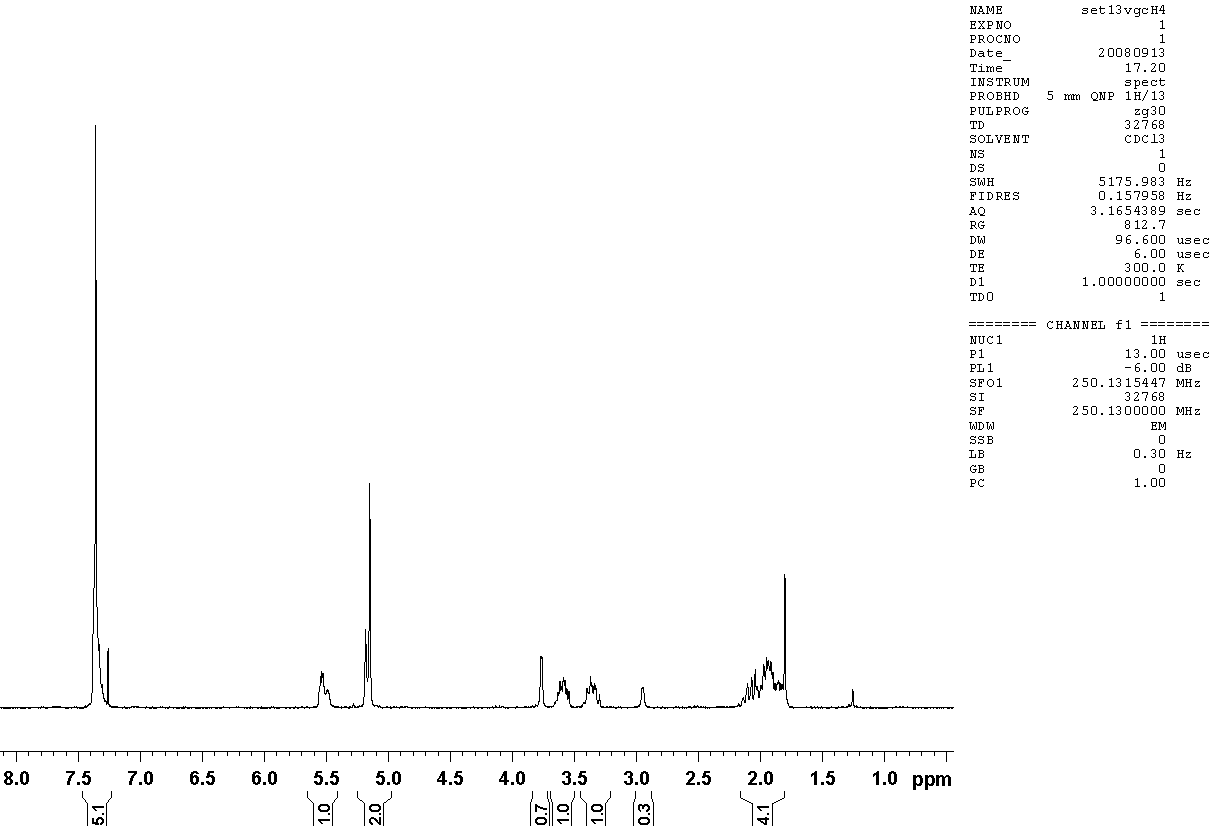


^1^H NMR (CDCl_3_, 250 MHz) of **3**.


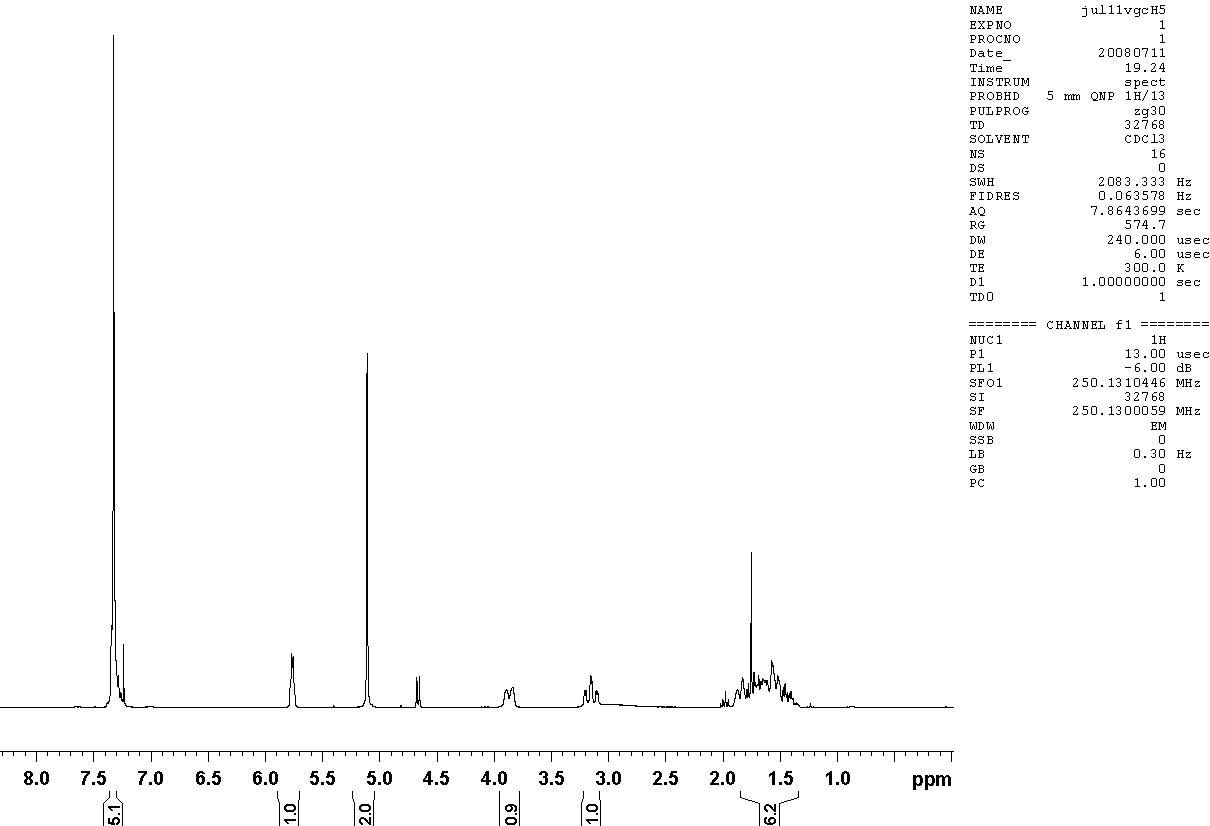


^1^H NMR (CDCl_3_, 250 MHz) of **4**.


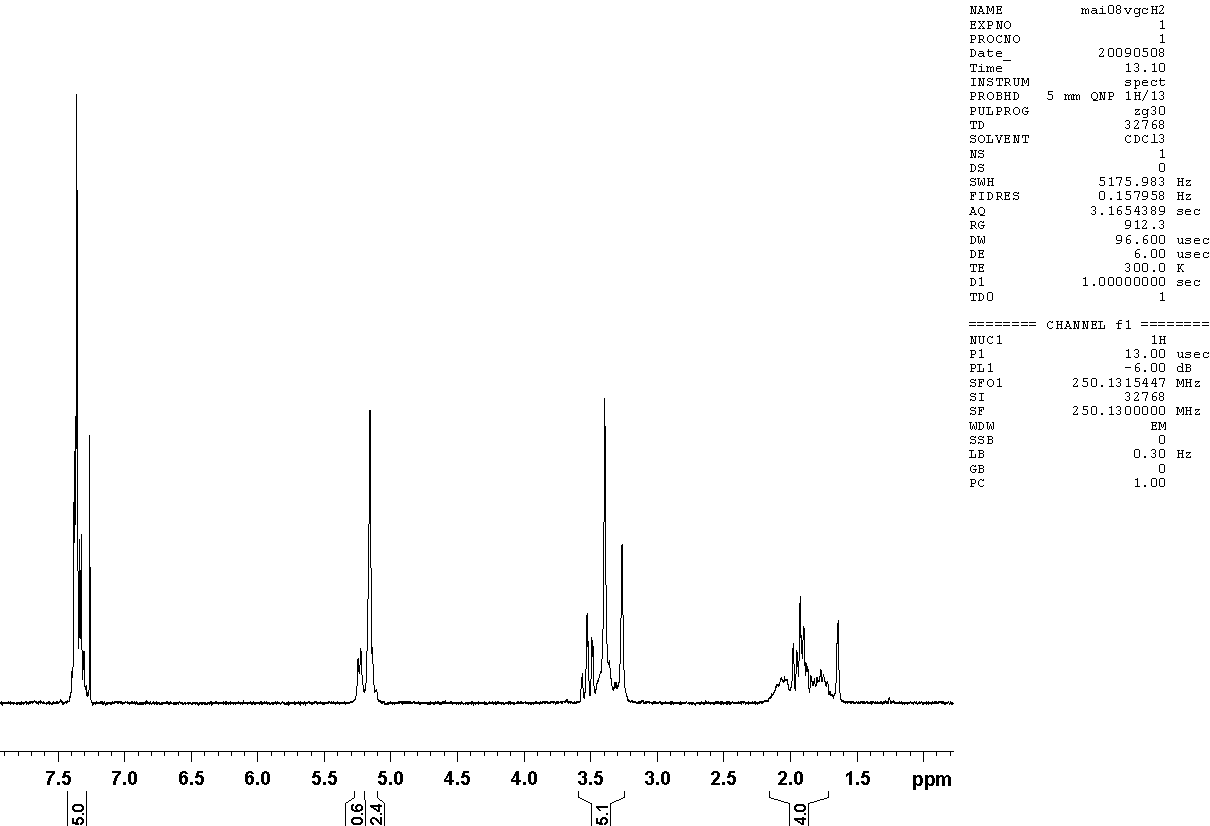


^1^H NMR (CDCl_3_, 250 MHz) of **5**.


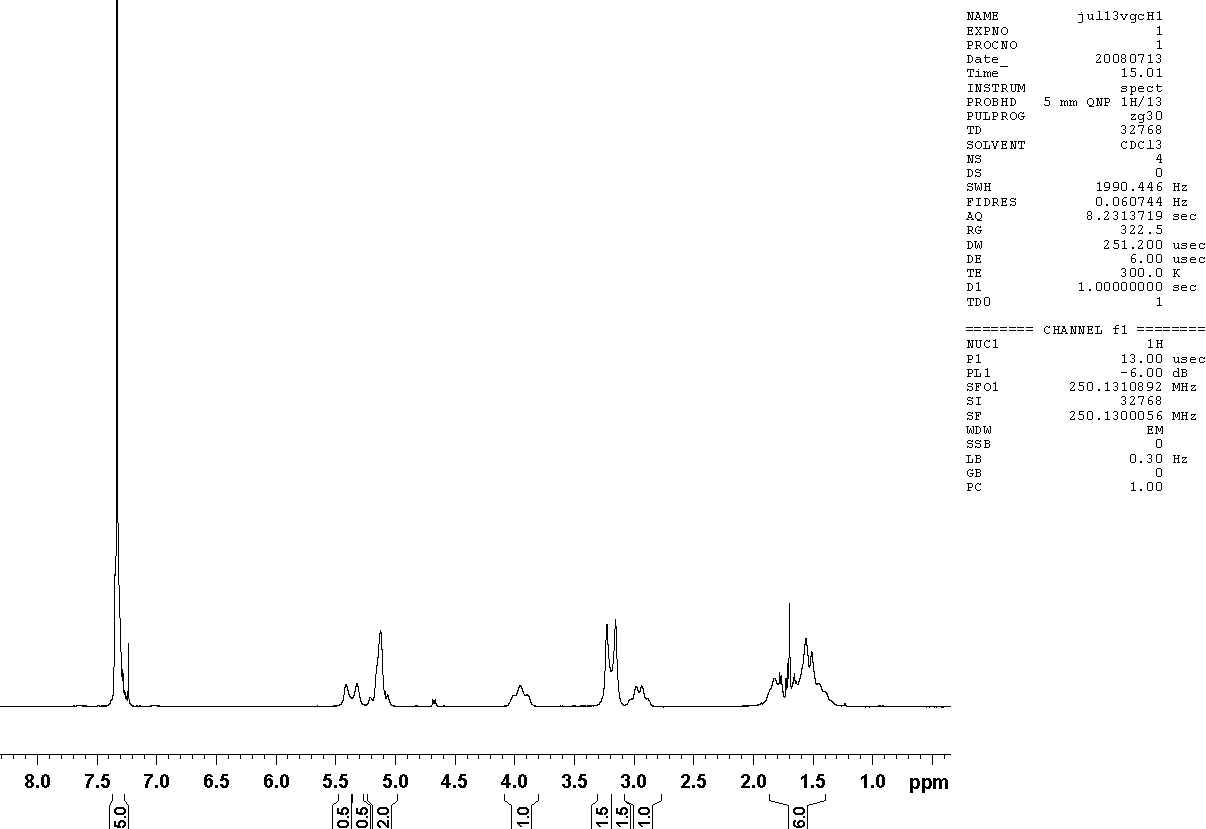


^1^H NMR(CDCl_3_, 250 MHz) of **6**.


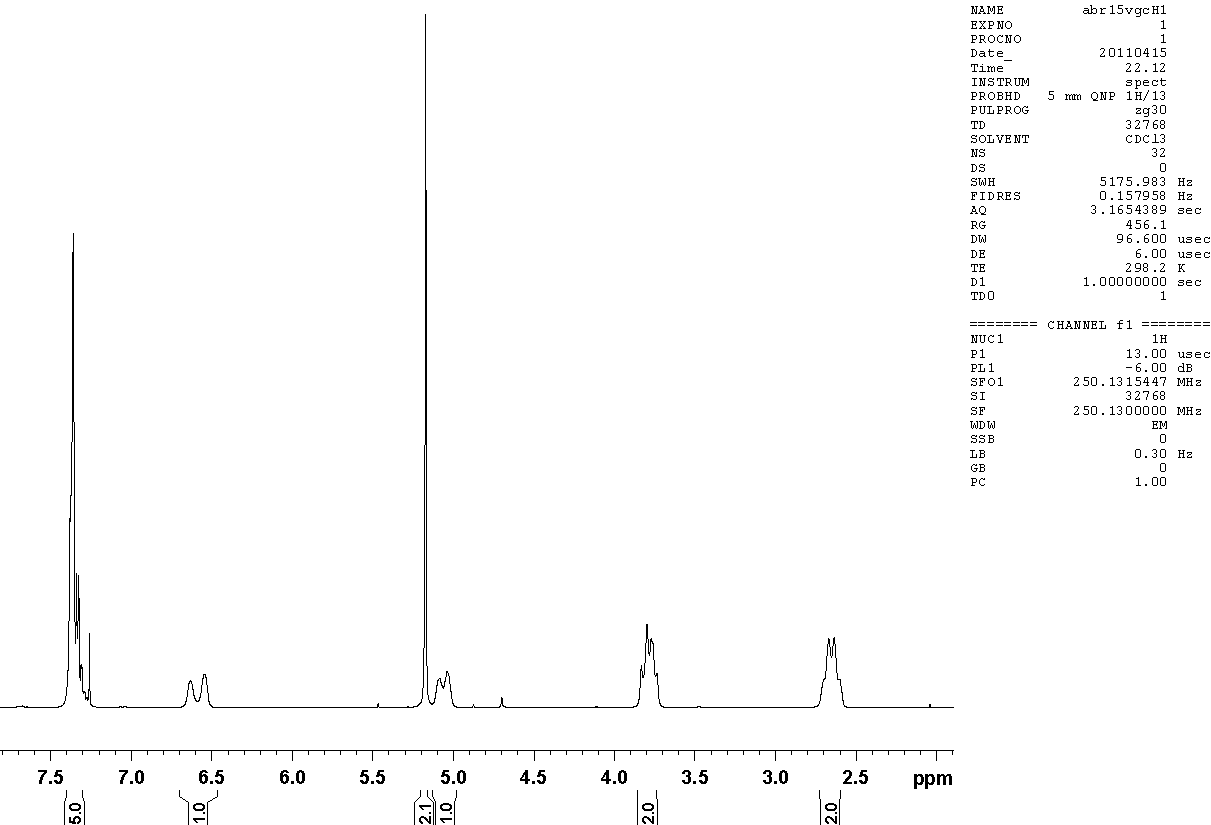


^1^H NMR (CDCl_3_, 250 MHz) of **7**.


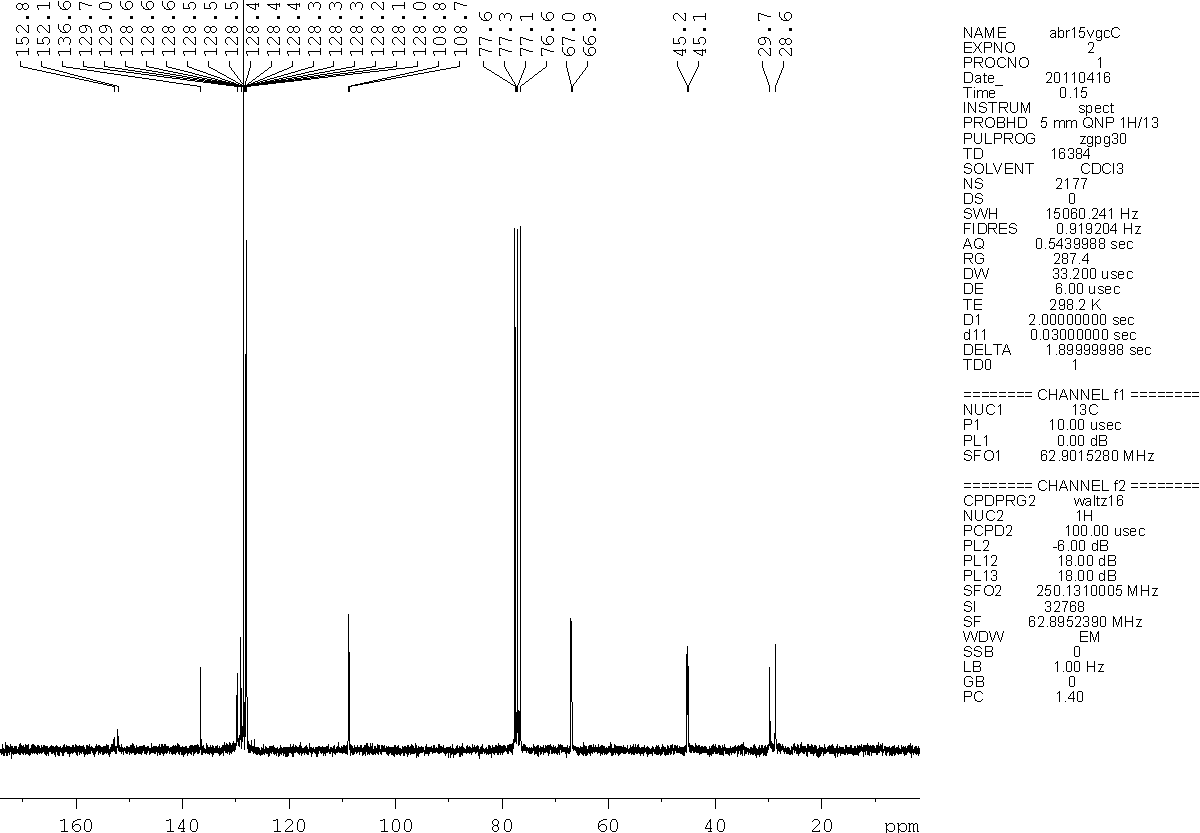


^13^C NMR (CDCl_3_, 62,5 MHz) of **7**.


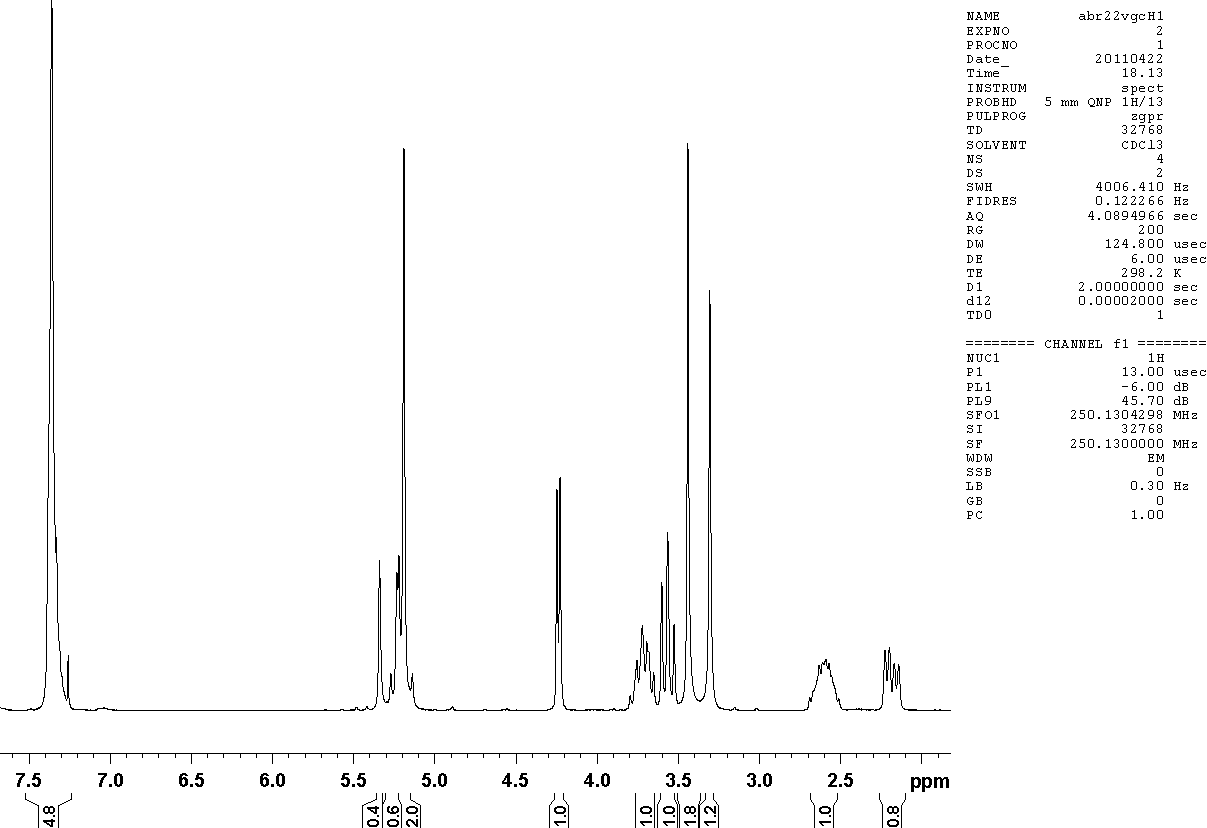


^1^H NMR (CDCl_3_, 250 MHz) of **8.**


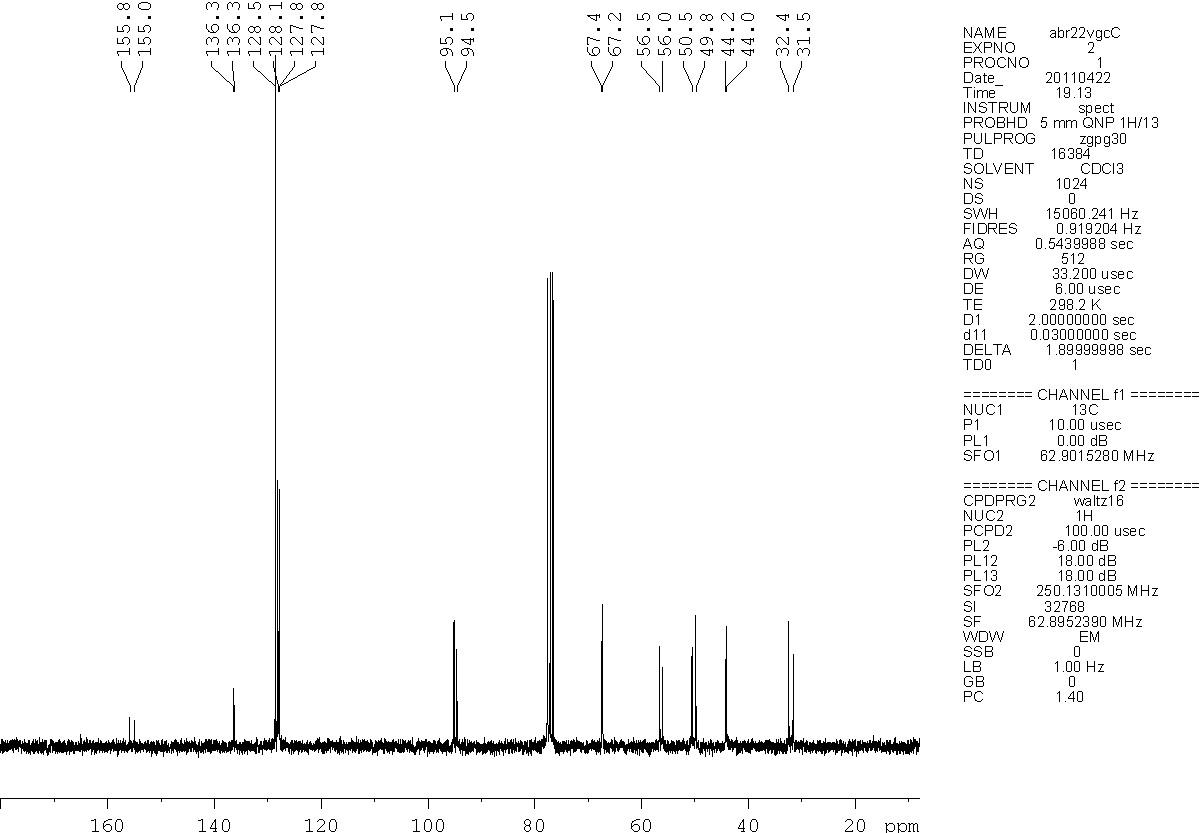


^13^C NMR (CDCl_3_, 62,5 MHz) of **8**.


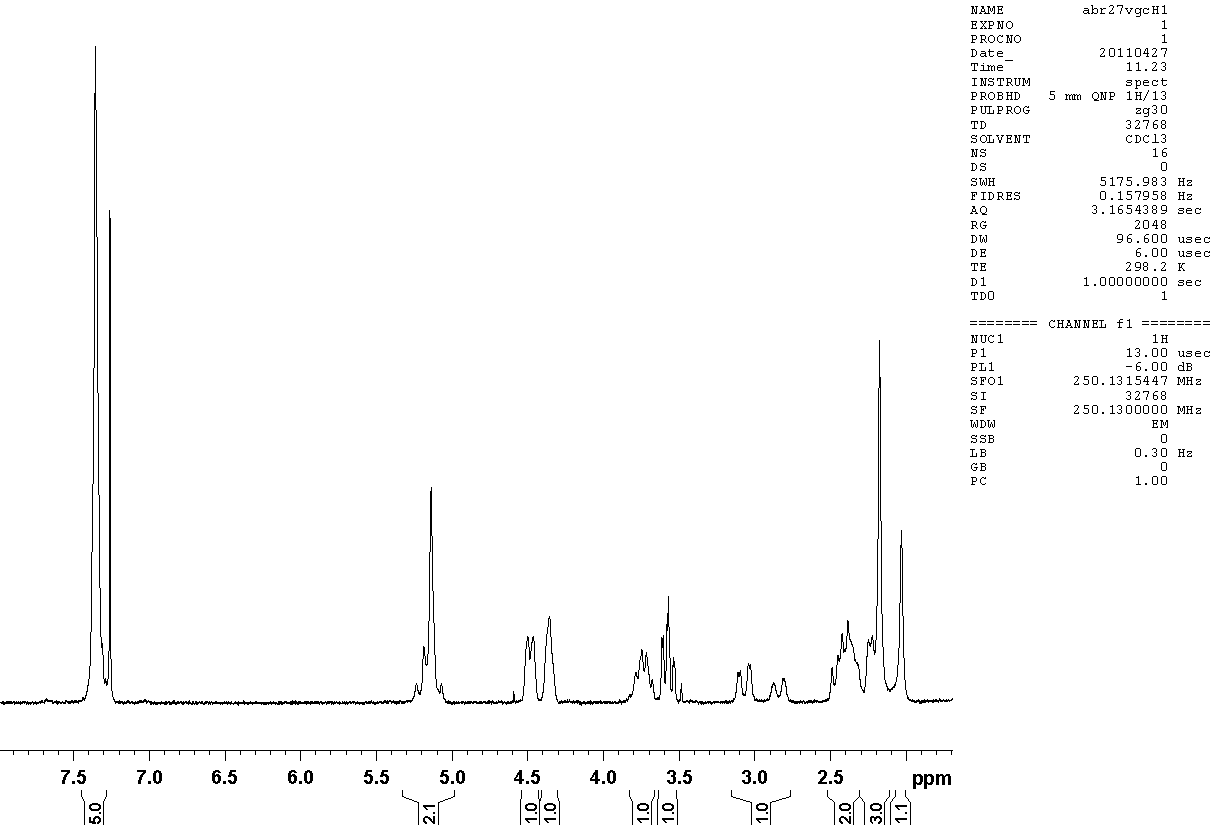


1H NMR (CDCl3, 250 MHz) of **9**.


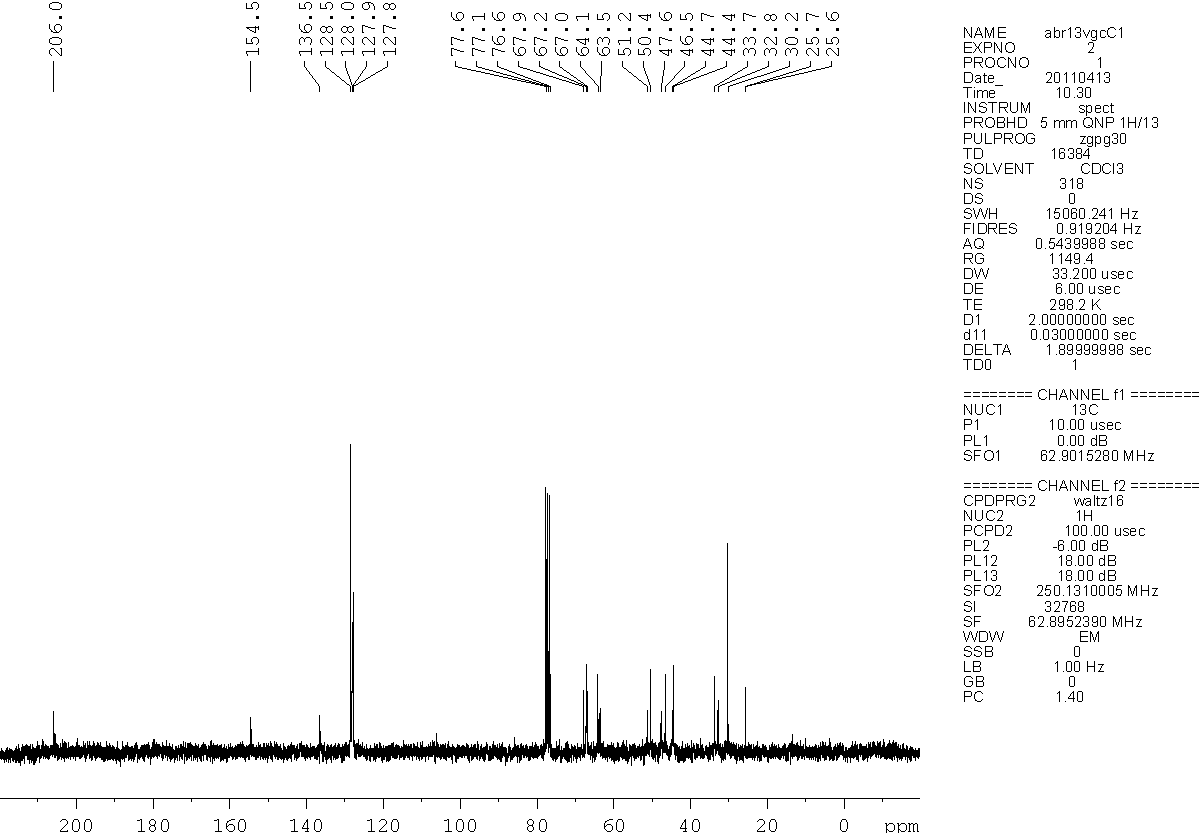


^13^C NMR (CDCl_3_, 62,5 MHz) of **9**.


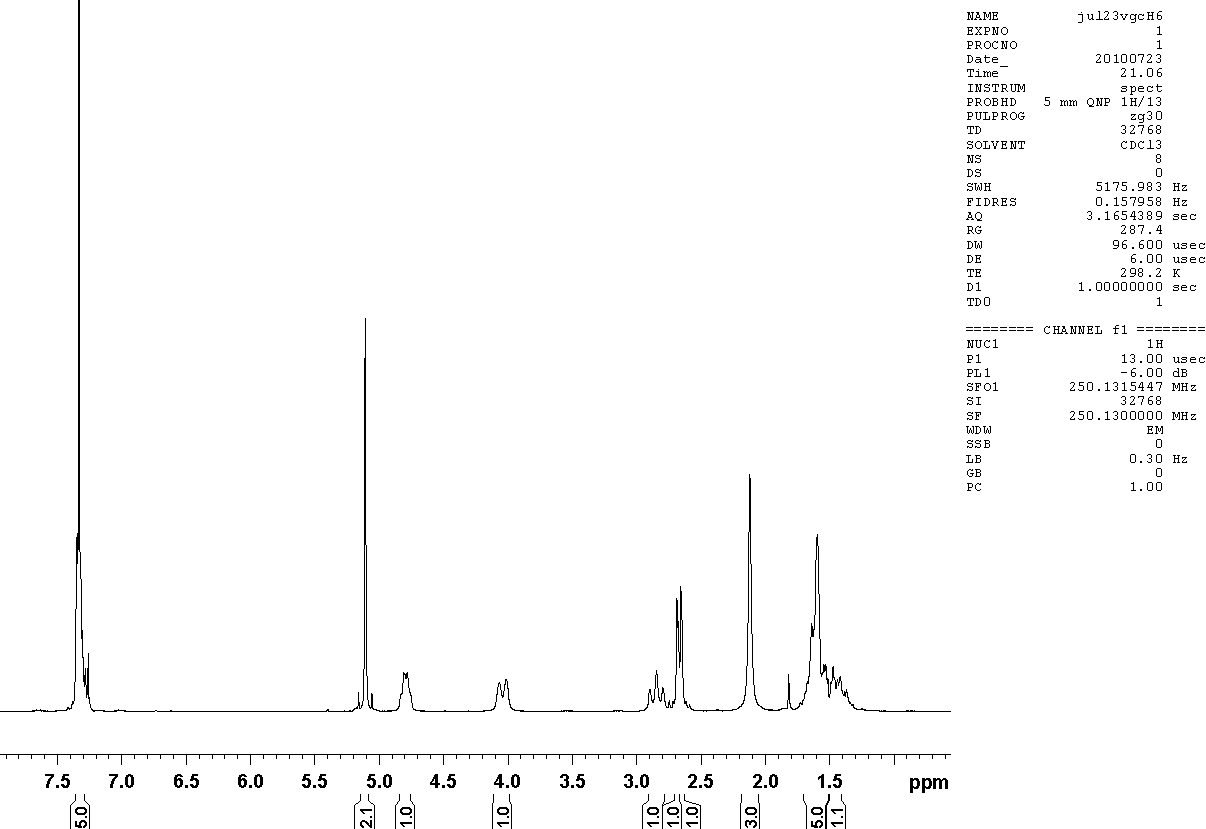


^1^H NMR (CDCl_3_, 250 MHz) of **10**.


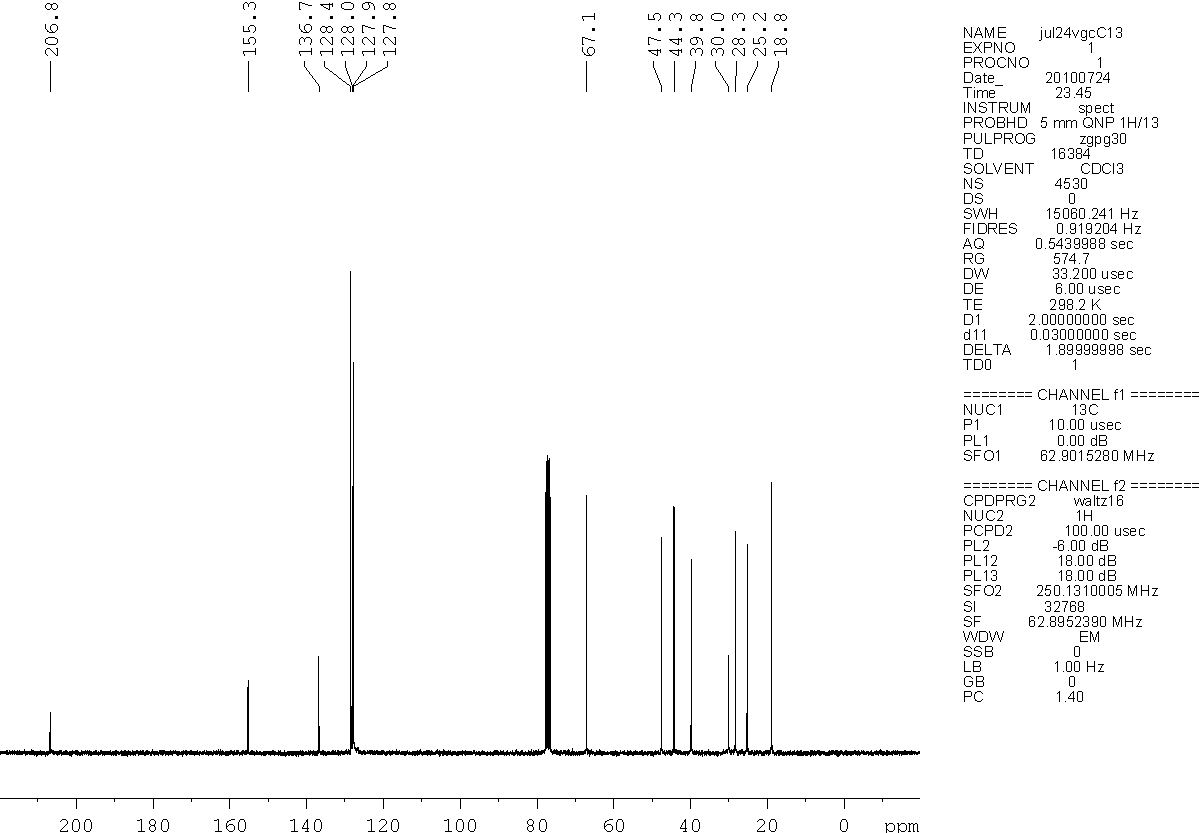


^13^C NMR (CDCl_3_, 62.5 MHz) of **10**


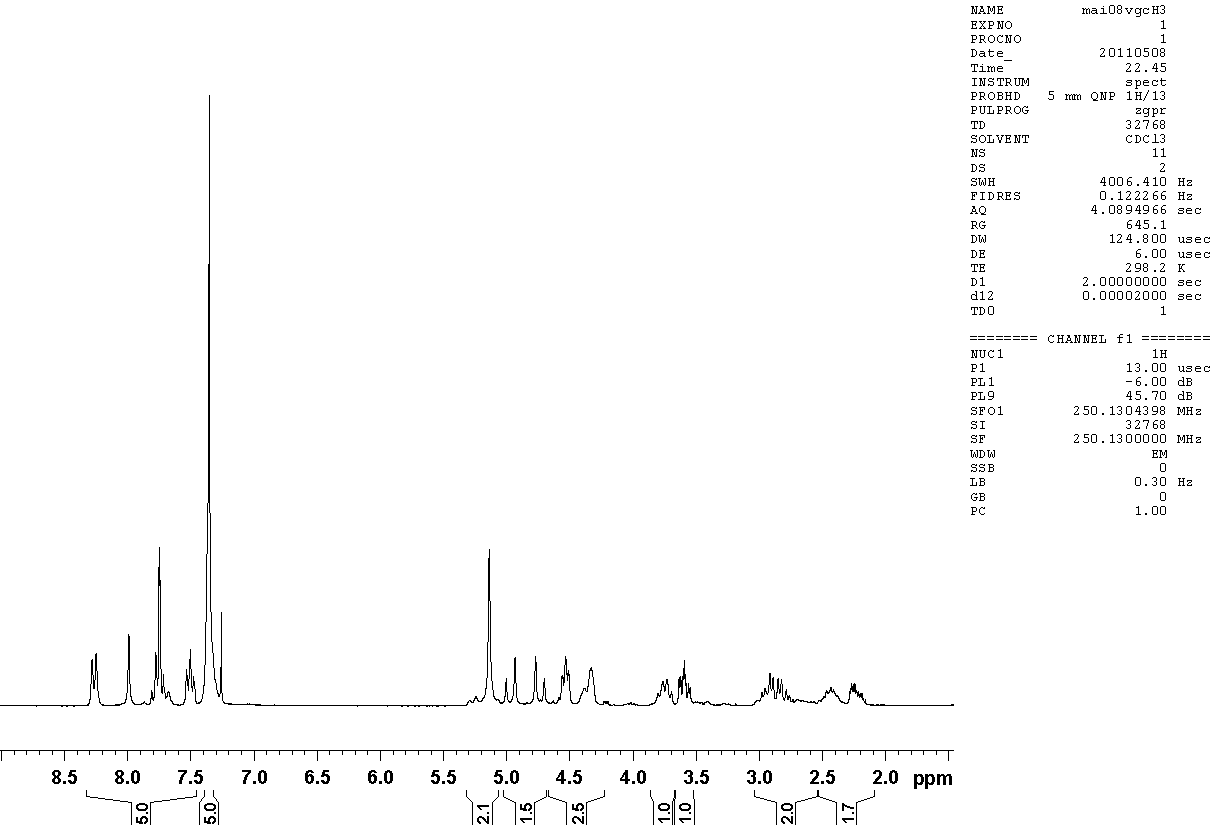


^1^H (CDCl_3_, 250 MHz) of **11**.


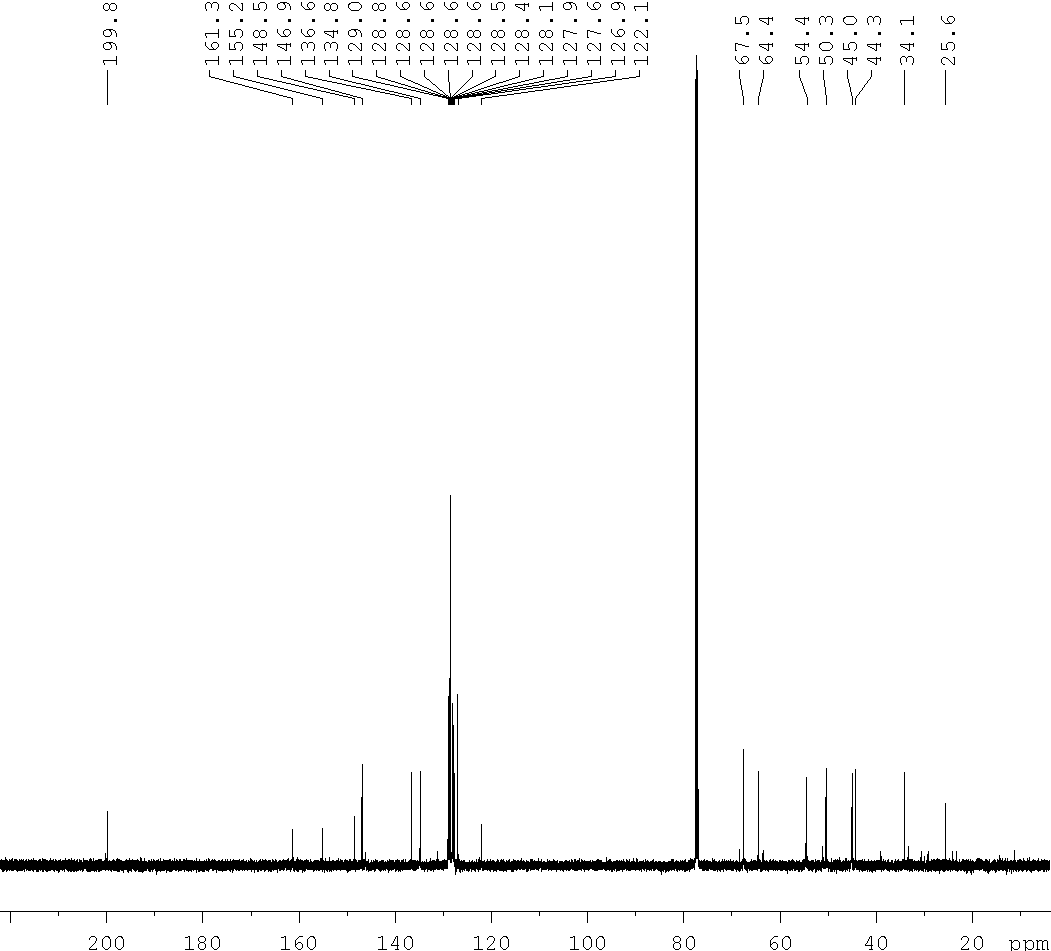


^13^C NMR (CDCl_3_,125 MHz) of **11**.


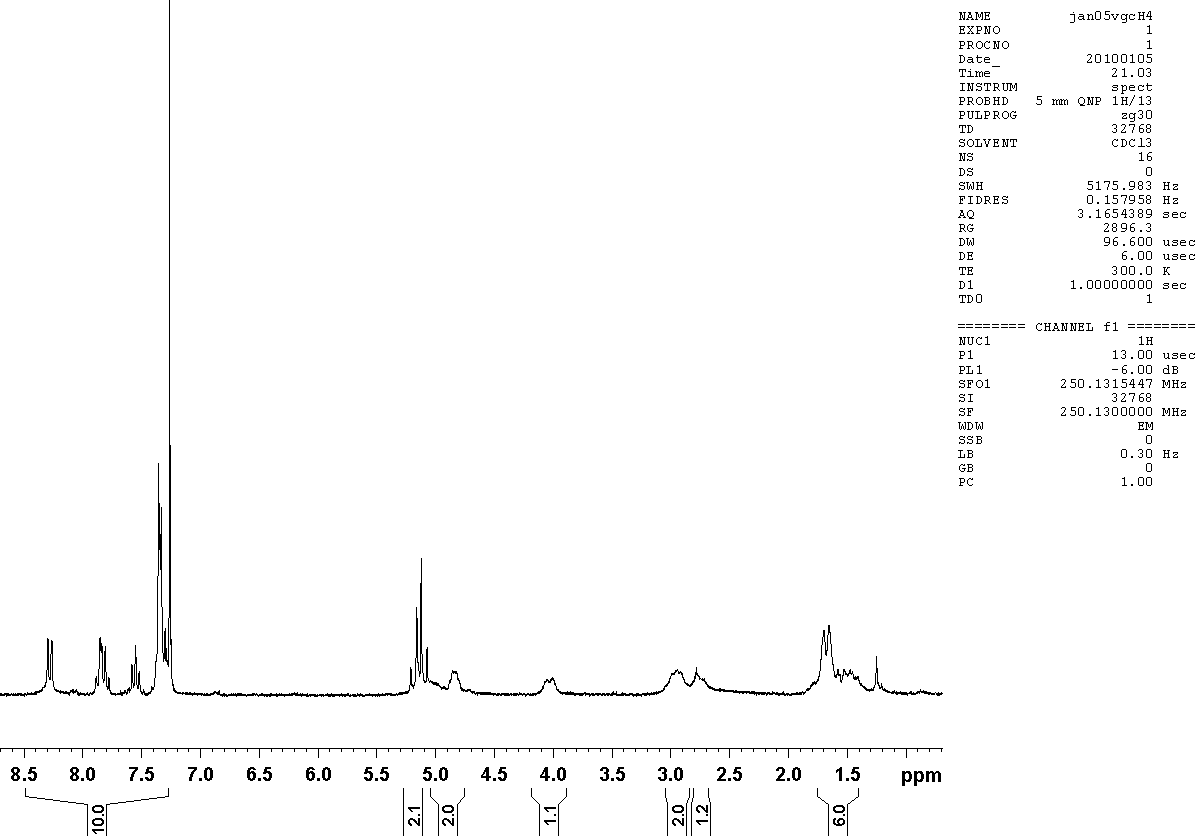


^1^H NMR (CDCl_3_, 250 MHz) of **12**.


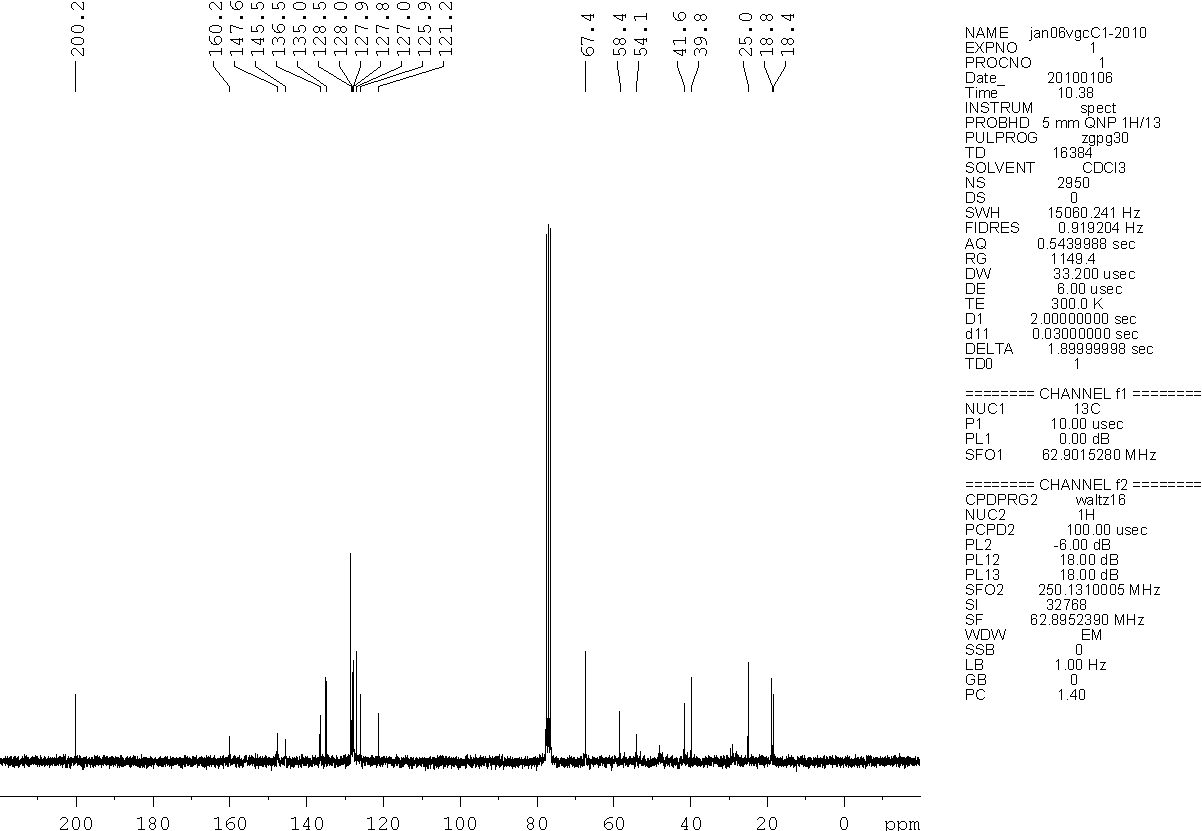


^13^C NMR (CDCl_3_, 62.5 MHz) of **12**.


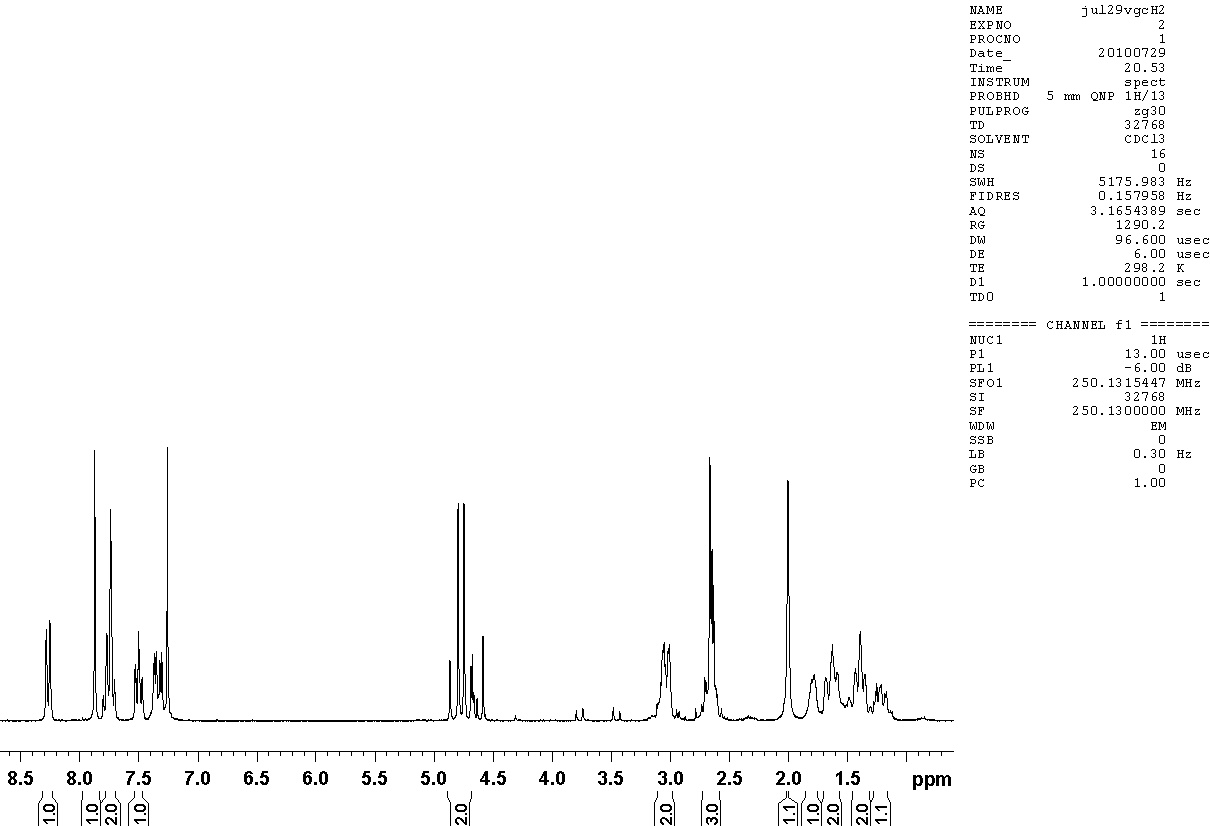


^1^H NMR (CDCl_3_, 250 MHz) of **d-FF.**


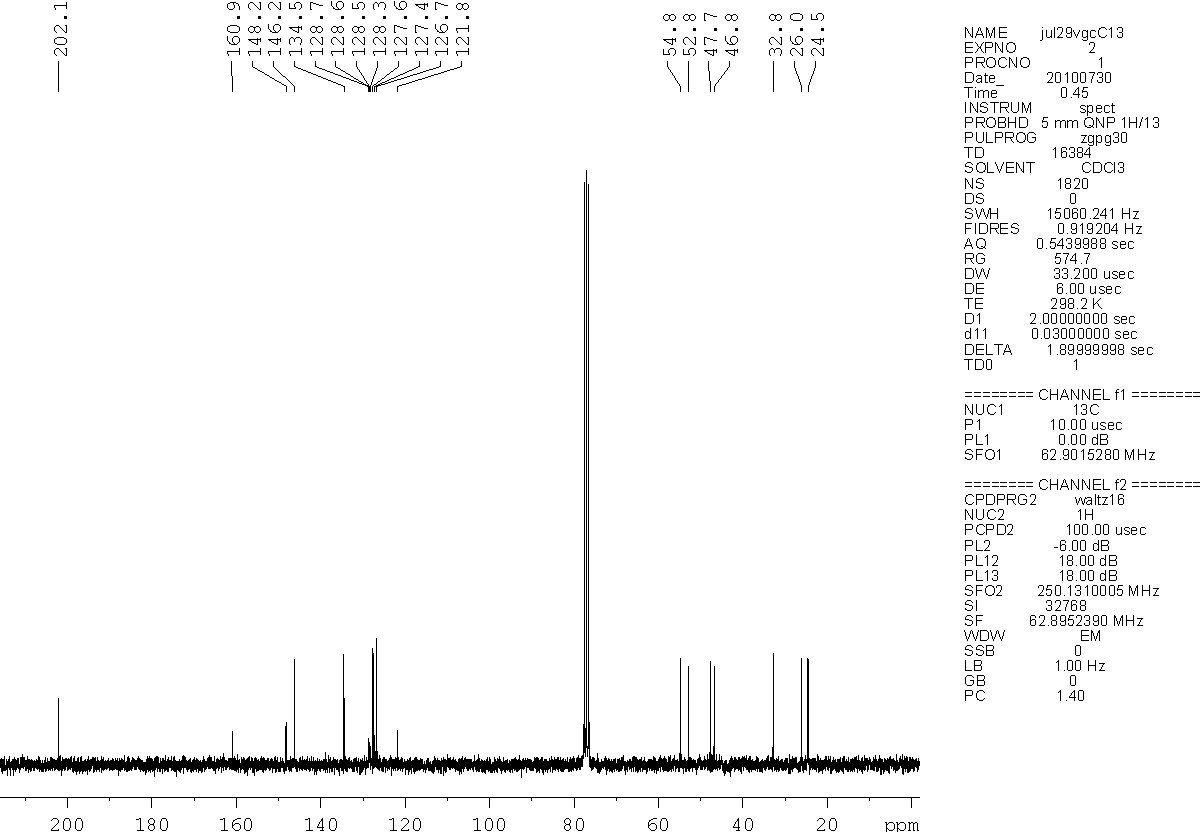


^13^C NMR (CDCl_3_, 62,5 MHz) of **d-FF**


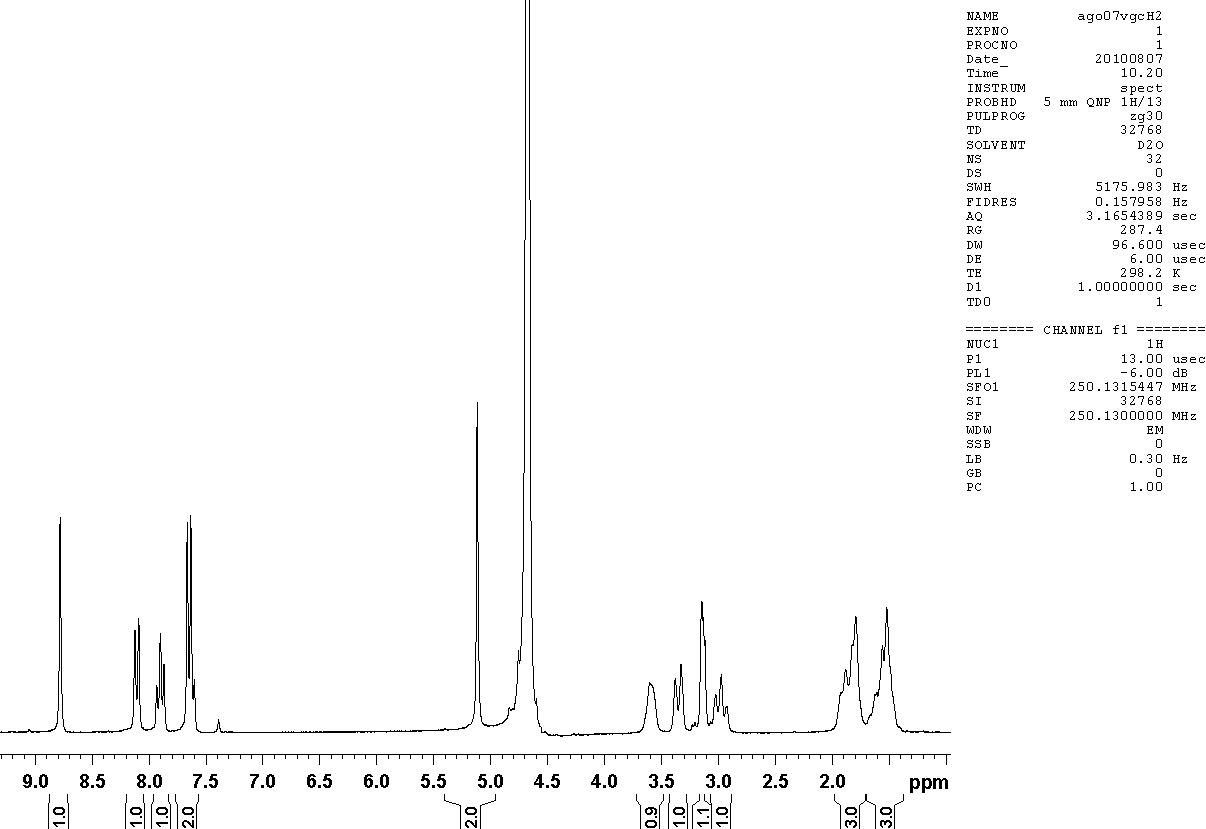


^1^H NMR (D_2_O, 250 MHz) of **d-FF**.HCl.


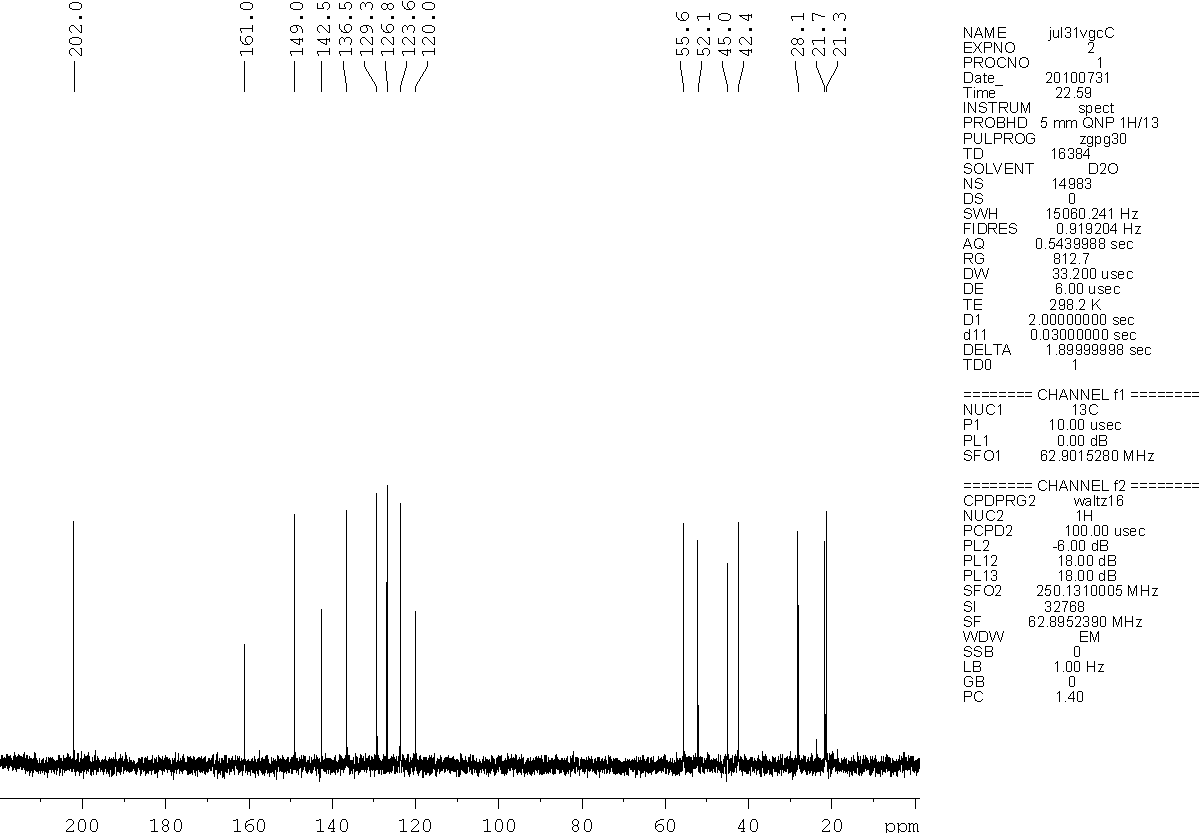


^13^C NMR (D_2_O, 62,5 MHz) of **d-FF**.HCl.


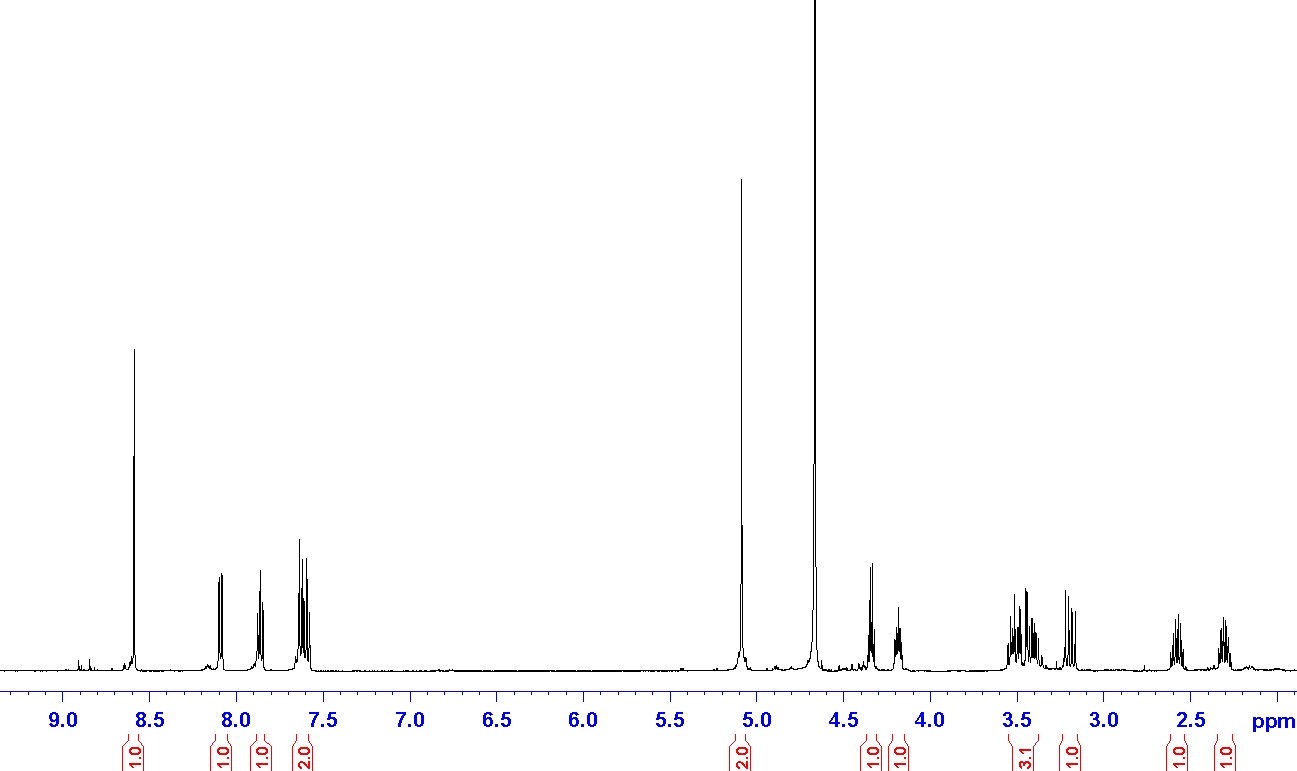


^1^H NMR (D_2_O, 500 MHz) of **Cpd-5**.HCl.


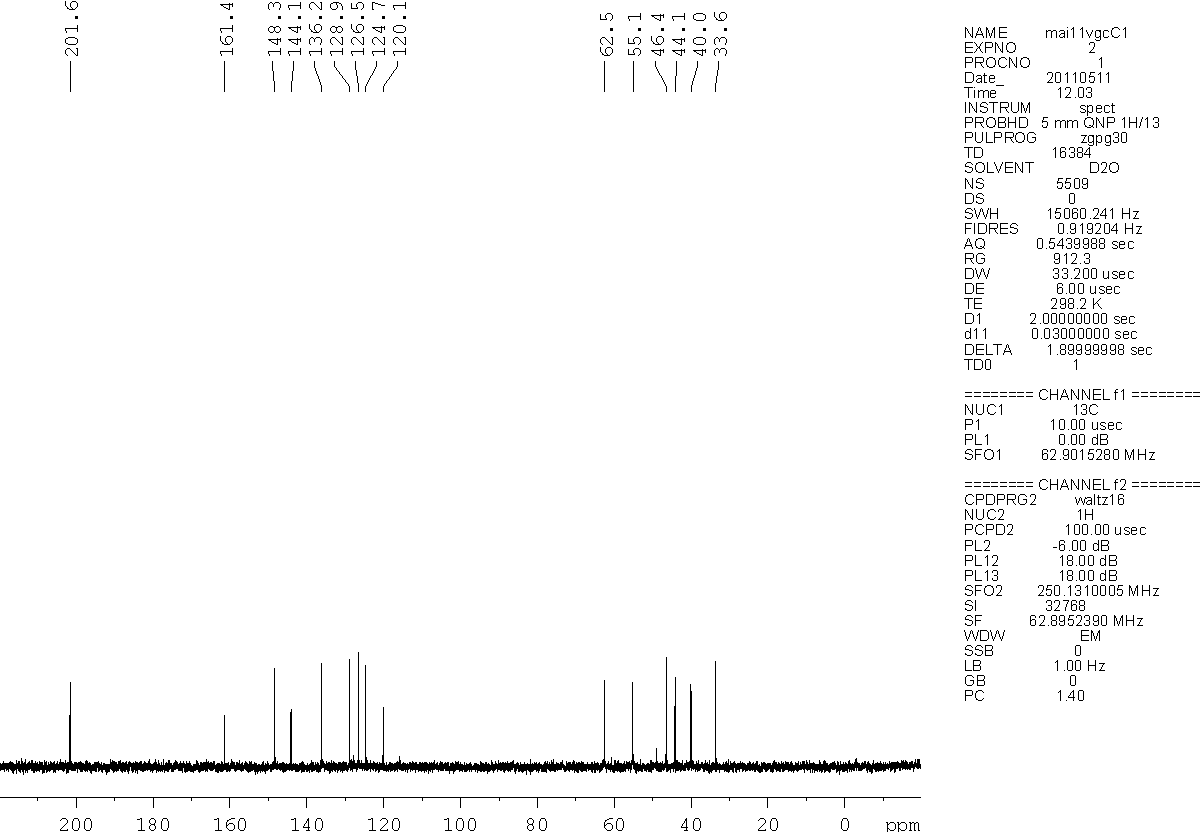


^13^C NMR (D_2_O, 62**.**5 MHz) of **Cpd-5.**HCl

1. Nagasaka, T.; Tamano, H.; Maekawa, T.; Hamaguchi, F. *Heterocycles*  **1987**, *26*, 617. [↑](#footnote-ref-1)
2. Matsumura, Y.; Ikeda, T.; Onomura, O. *Heterocycles* **2006**, *67*, 113. [↑](#footnote-ref-2)
